# Identification of telomere-associated molecules by engineered DNA-binding molecule-mediated chromatin immunoprecipitation (enChIP)

**DOI:** 10.1038/srep03171

**Published:** 2013-11-08

**Authors:** Toshitsugu Fujita, Yoshinori Asano, Junko Ohtsuka, Yoko Takada, Kazunobu Saito, Rieko Ohki, Hodaka Fujii

**Affiliations:** 1 Combined Program on Microbiology and Immunology, Research Institute for Microbial Diseases, Osaka University, 3-1 Yamadaoka, Suita, 565-0871 Osaka, Japan; 2 Division of Refractory Cancer Research, National Cancer Center Research Institute, 5-1-1 Tsukiji, Chuo-ku, Tokyo 104-0045, Japan; 3 Integrative Bioscience and Biomedical Engineering, Graduate School of Science and Engineering, Waseda University, Tokyo, Japan; 4 Division of Immunobiology, Research Institute for Biomedical Sciences, Tokyo University of Science, Chiba, Japan; 5 Core Instrumentation Facility, Immunology Frontier Research Center, Osaka University, 3-1 Yamadaoka, Suita, 565-0871 Osaka, Japan; 6 DNA-chip Development Center for Infectious Diseases, Research Institute for Microbial Diseases, Osaka University, 3-1 Yamadaoka, Suita, 565-0871 Osaka, Japan

## Abstract

Biochemical analysis of molecular interactions in specific genomic regions requires their isolation while retaining molecular interactions *in vivo*. Here, we report isolation of telomeres by engineered DNA-binding molecule-mediated chromatin immunoprecipitation (enChIP) using a transcription activator-like (TAL) protein recognizing telomere repeats. Telomeres recognized by the tagged TAL protein were immunoprecipitated with an antibody against the tag and subjected to identification of telomere-binding molecules. enChIP-mass spectrometry (enChIP-MS) targeting telomeres identified known and novel telomere-binding proteins. The data have been deposited to the ProteomeXchange with identifier PXD000461. In addition, we showed that RNA associated with telomeres could be isolated by enChIP. Identified telomere-binding molecules may play important roles in telomere biology. enChIP using TAL proteins would be a useful tool for biochemical analysis of specific genomic regions of interest.

Molecular complexes in the context of chromatin mediate functions of eukaryotic genome^1^. Identification of chromatin components is essential for elucidation of molecular mechanisms of genome functions. We recently developed the insertional chromatin immunoprecipitation (iChIP) technology to isolate specific genomic regions retaining molecular interactions *in vivo*^2^,^3^. In iChIP, recognition sequences of an exogenous DNA-binding molecule such as LexA are inserted into a target genomic region. Subsequently, the tagged genomic region is affinity-purified and subjected to downstream analyses such as mass spectrometry (MS) (iChIP-MS) to identify proteins and RT-PCR (iChIP-RT-PCR) to identify RNA^3^,^4^. Although iChIP enables us to isolate specific genomic regions of interest and dissect chromatin components, insertion of recognition sequences of LexA or other DNA-binding molecules is a time-consuming step.

To eliminate the step of insertion of recognition sequences of an exogenous DNA-binding molecule from iChIP and make the procedure more straightforward, we recently developed a novel method, engineered DNA-binding molecule-mediated chromatin immunoprecipitation (enChIP), for purification of specific genomic regions^5^. In enChIP, a tagged engineered DNA-binding molecule recognizing an endogenous target DNA sequence is expressed into the cell to be analyzed. Subsequently, the target genomic region is subjected to affinity-purification such as immunoprecipitation with an antibody (Ab) against the tag.

In the present study, we isolated telomeres by enChIP using a transcription activator-like (TAL) protein recognizing telomere repeats. enChIP-mass spectrometry (enChIP-MS) targeting telomeres identified known and novel telomere-binding proteins. In addition, we showed that RNA associated with telomeres could be detected by enChIP combined with RT-PCR (enChIP-RT-PCR). Identified telomere-binding molecules may play important roles in telomere biology. Thus, enChIP using TAL proteins would be a useful tool for biochemical analysis of specific genomic regions of interest.

## Results

### Scheme of enChIP

**Figure 1:**
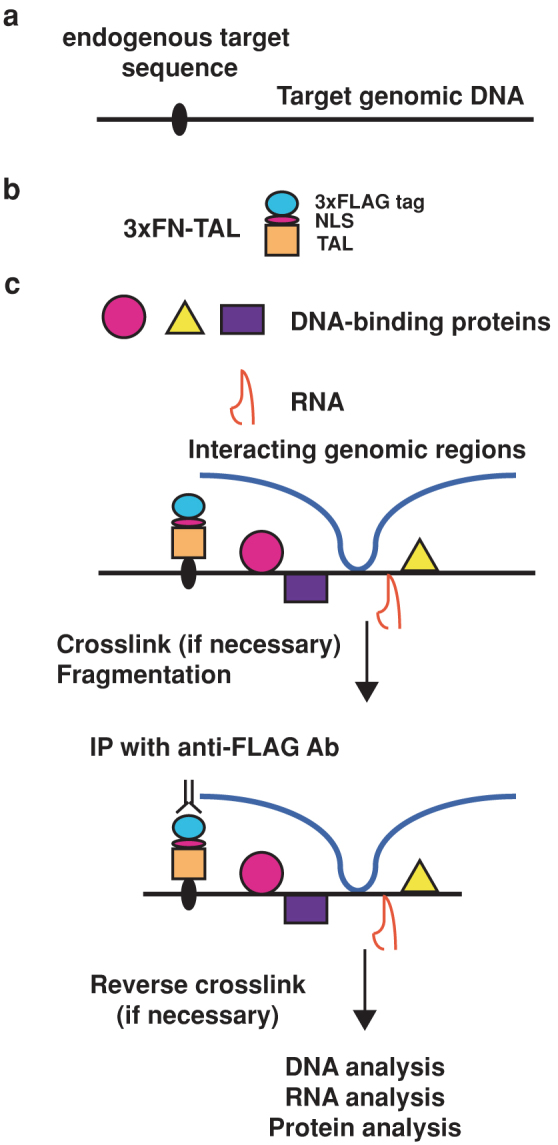
Scheme of engineered DNA-binding molecule-mediated chromatin immunoprecipitation (enChIP) using TAL proteins. The enChIP system consists of a fusion molecule consisting of a tag(s), the nuclear localization signal (NLS), and an engineered DNA-binding molecule interacting with target DNA sequence (a, b). 3xFLAG-tag and a transcription activator-like (TAL) protein are shown as examples of tags and engineered DNA-binding proteins, respectively. (c) The 3xFN-TAL is expressed in appropriate cells. The cells are crosslinked, if necessary, lysed, and fragmented by sonication or other methods. The chromatin complexes are immunoprecipitated with anti-FLAG Ab, and crosslink is reversed when crosslinkers are used. Molecules (DNA, RNA, proteins, and others) associated with the target genomic region are isolated and characterized.

enChIP consists of the following steps^5^ (Fig. 1):

- A DNA-binding molecule/complex (DB) recognizing a target DNA sequence in a genomic region of interest is engineered (Fig. 1a, b). Zinc-finger proteins^6^, TAL proteins^7^, and a catalytically inactive Cas9 (dCas9) plus small guide RNA (gRNA)^8^ can be used as DB. A tag(s) and the nuclear localization signal (NLS) are fused with the engineered DB (Fig. 1b), and expressed into the cell to be analyzed.
- If necessary, the resultant cell is stimulated and crosslinked with formaldehyde or other crosslinkers.
- The cell is lysed, and chromatin is fragmented by sonication or digested with nucleases.
- The chromatin complexes containing the engineered DB are affinity-purified by immunoprecipitaion or other methods.
- After reverse crosslinking, DNA, RNA, proteins, or other molecules are purified and subjected to identification by various methods including next generation sequencing and mass spectrometry (Fig. 1c).

### Isolation of telomeres by enChIP using a TAL protein

**Figure 2:**
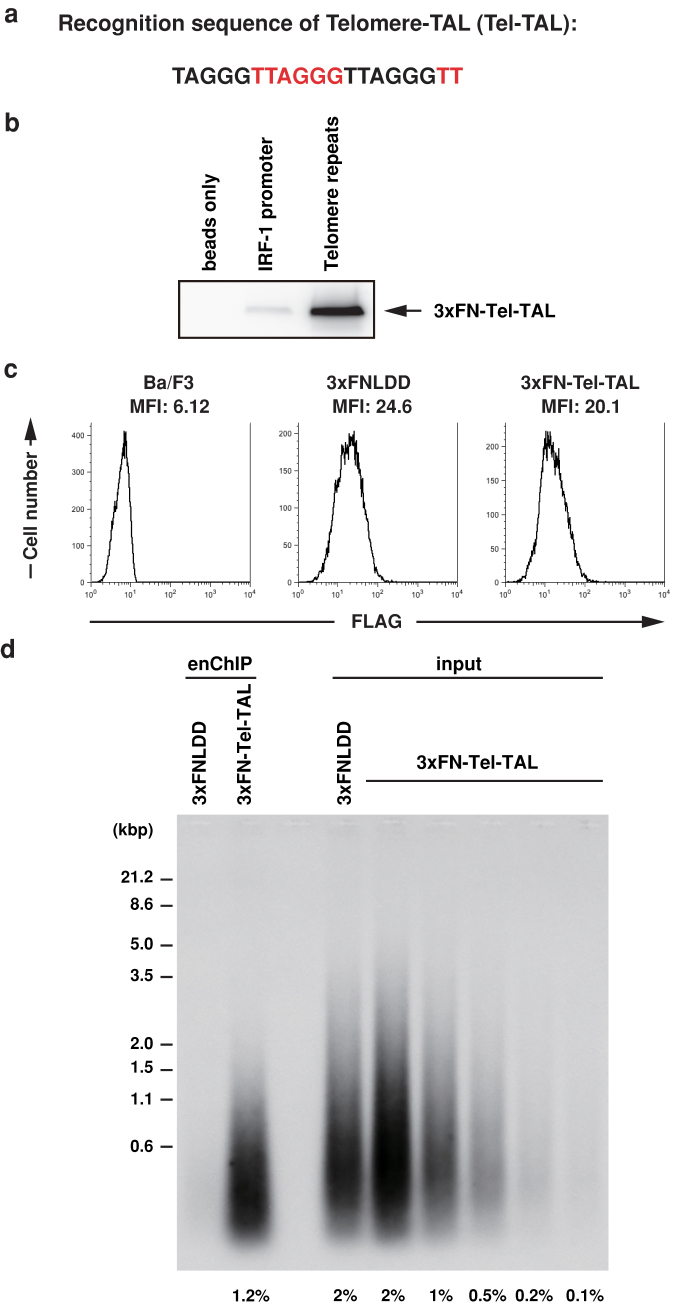
Isolation of telomeres by enChIP with Telomere-TAL. (a) The recognition sequence of Telomere-TAL (Tel-TAL) containing a 19-bp telomere repeat. The telomeric TTAGGG sequence is shown in black and red. (b) Specific binding of 3xFN-Tel-TAL to telomere repeats *in vitro*. DNAP assay was performed with nuclear extracts from 293T cells expressing 3xFN-Tel-TAL and the biotinylated telomere repeats or IRF-1 promoter DNA. Immunoblot analysis was performed with anti-FLAG Ab. The full-length blot with size markers is shown in Supplemental Figure 2. (c) Expression levels of 3xFN-Tel-TAL and control 3xFNLDD proteins detected by flowcytometry using anti-FLAG Ab. (d) Southern blot analysis of chromatin complexes isolated by enChIP using 3xFN-Tel-TAL. Chromatin from 2 × 10^7^ cells was used for enChIP. The full-length blot with size markers is shown in Supplemental Figure 3.

To isolate telomeres, a TAL protein, Telomere-TAL (Tel-TAL), recognizing a 19-bp sequence containing an array of TTAGGG (telomere repeats) (Fig. 2a) was fused with 3xFLAG tag and NLS (3xFN-Tel-TAL) (Fig. 1b, Supplemental Fig. 1). First, we examined binding of 3xFN-Tel-TAL to telomere repeats or irrelevant *interferon regulatory factor-1 (IRF-1)* promoter sequence *in vitro* using DNA-affinity precipitation assay (DNAP)^9^. As shown in Fig. 2b (the full-length blot with size markers is shown in Supplemental Fig. 2), binding of 3xFN-Tel-TAL to telomere repeats was clearly detected, whereas its binding to irrelevant *IRF-1* promoter sequence was marginal, showing that binding of 3xFN-Tel-TAL is specific to telomere repeats *in vitro*. Next, 3xFN-Tel-TAL was expressed in a mouse hematopoietic cell line, Ba/F3. Ba/F3 expresses functional telomerase^10^. Expression of 3xFN-Tel-TAL or a negative control protein consisting of 3xFLAG-tag, NLS, and LexA protein (3xFNLDD)^11^ was confirmed by flowcytometry with anti-FLAG M2 Ab (Fig. 2c). The cells were crosslinked with formaldehyde, and crosslinked chromatin was fragmented by sonication. Subsequently, chromatin complexes containing 3xFN-Tel-TAL or 3xFNLDD were immunoprepicitated with anti-FLAG M2 Ab. Southern blot analysis using a telomere probe showed that telomere DNA was specifically detected in the immunoprecipitants prepared from Ba/F3 expressing 3xFN-Tel-TAL. 1.2% of input genomic DNA was immunoprecipitated with 3xFN-Tel-TAL (Fig. 2d, the full-length blot with size markers is shown in Supplemental Fig. 3). In contrast, irrelevant γ-satellite repeats were not specifically enriched in the immunoprecipitants prepared from Ba/F3 expressing 3xFN-Tel-TAL (Supplemental Fig. 4). These results showed that enChIP with 3xFN-Tel-TAL can isolate telomeres specifically.

### Identification of telomere-binding proteins by enChIP-MS

Next, enChIP-MS was used to perform non-biased search for proteins associated with telomeres. 4 × 10^7^ cells were subjected to enChIP followed by elution with 3xFLAG peptide. The eluate was reverse-crosslinked and subjected to SDS-PAGE. After staining with Coomassie Brilliant Blue, proteins were excised for MS analysis (Supplemental Fig. 5). We detected many telomere-related proteins (Table 1, Supplemental Table 1). They included known telomere-binding proteins, proteins interacting with telomere-binding proteins, and proteins whose mutations affect telomere function. These data clearly showed that it is feasible to identify proteins interacting with endogenous genomic regions by enChIP-MS.

### Co-localization of novel telomere-binding proteins with telomeres

**Figure 3:**
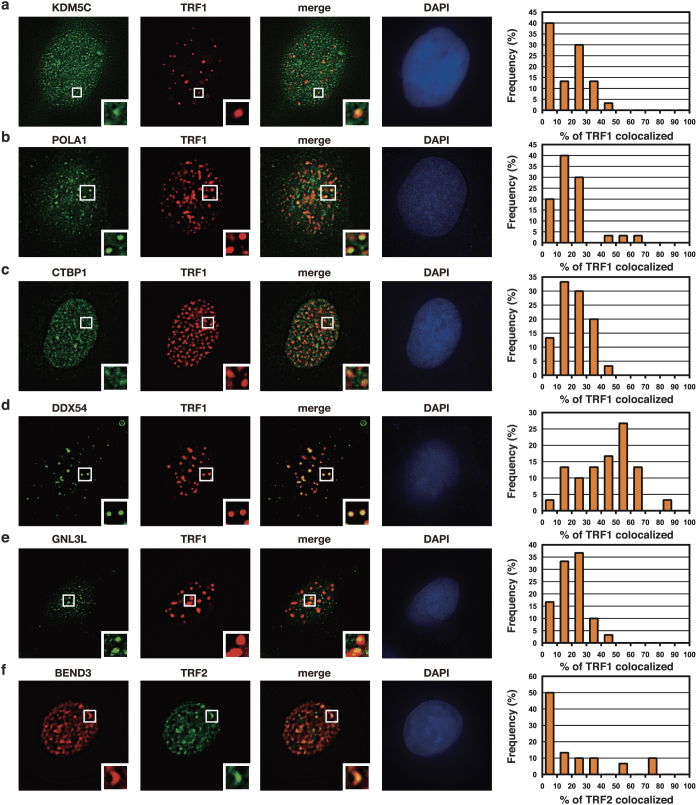
Localization of candidate proteins at telomeres. (a–e) U2OS cells were transfected with the expression vector of DsRed-TRF1. Cells were fixed with 4% paraformaldehyde 24 h post transfection. The fixed cells were sequentially incubated with Abs against the indicated proteins and AlexaFluor 488-conjugated secondary Ab, and subjected to immunofluorescence microscopy. Thirty cells expressing DsRed-TRF1 were randomly picked up and the frequency of signals overlapping TRF1 signals were counted and shown as graphs. For detection of POLA1 and DDX54, cells were permeabilized with PBS containing 0.2% Triton X-100 for 2 min before fixation. (f) U2OS cells were transfected with the expression vector of ECFP-TRF2 together with that of Halo-tagged BEND3. Cells were treated with TMR ligand (5 μM) for 15 min at 37°C 23 h post transfection, fixed with 4% paraformaldehyde, and subjected to immunofluorescence microscopy. Thirty cells expressing both ECFP-TRF2 and Halo-tagged BEND3 were randomly picked up and analyzed as in (a–e).

The above-mentioned list contained many proteins whose localization at mammalian telomeres has not been reported. We examined localization of several of them at telomeres in U2OS cells by imaging analysis. Immunofluorescence microscopy with Abs against endogenous proteins showed that selected candidate proteins (KDM5C, POLA1, CTBP1, DDX54, GNL3L) co-localized with TRF1, a marker protein of telomeres (Fig. 3a–e). Consistently, Halo-tagged KDM5C and CTBP1 also showed co-localization with TRF2, another maker protein of telomeres (Supplemental Fig. 6). In addition, Halo-tagged BEND3 protein co-localized with TRF2 (Fig. 3f). Detected telomere-binding proteins showed a variety of localization patterns with telomeres (Fig. 3). KDM5 is a lysine-specific histone demethylase. It has been shown that its yeast homologue LSD1 localizes in heterochromatin including subtelomeric regions and their mutations cause spreading of telomeric heterochromatin^12^. POLA1 is DNA polymerase α catalytic subunit. It has been shown that its yeast homologue binds to telomere protein Cdc13p^13^. A mouse cell line expressing a temperature sensitive-mutant of POLA1 has been shown to have telomere defects^14^. It has been shown that CTBP (C-terminal binding protein) binds to FoxP2 protein, which is associated with POT1 telomere-binding protein^15^. DDX54 is a DEAD-box RNA helicase, and it has been reported that human DDX54 interacts with estrogen receptors (ERs) and represses transcription of target genes of ERs^16^. Involvement of DDX54 in telomere functions has not been reported. GNL3L (guanine nucleotide-binding protein-like 3-like protein) is also known as nucleostemin and a paralog of the stem cell-enriched GTP binding protein GNL3^17^. It has been shown that GNL3L binds to the telomerase complex and TRF1^18^. BEND3 (BEN domain-containing protein 3) has been shown to localize to heterochromatin associated with HP1^19^.

### Detection of telomerase RNA by enChIP-RT-PCR

**Figure 4:**
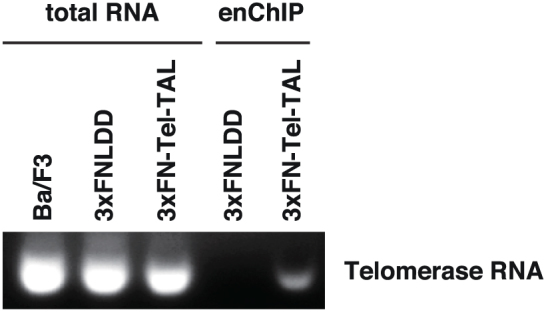
enChIP-RT-PCR detects telomere-associated RNA. After isolation of telomeres by enChIP, RNA purified from chromatin was treated with DNase and subjected to RT-PCR analysis to detect telomerase RNA. The full-length gel image with size markers is shown in Supplemental Figure 7.

Next, we examined whether RNA species interacting with telomeres can also be isolated by enChIP. After isolation of telomeres by enChIP from Ba/F3 cells, RNA purified from chromatin was treated with DNase and subjected to RT-PCR analysis to detect the RNA component of telomerase^20^,^21^. As shown in Fig. 4 (the full-length gel image with size markers is shown in Supplemental Fig. 7), telomerase RNA was clearly detected in chromatin isolated with 3xFN-Tel-TAL but not in the negative control sample. This result clearly showed that enChIP can be used for identification of RNA associated with target genomic regions.

## Discussion

In this study, we developed enChIP using a TAL protein for purification of specific genomic regions retaining molecular interactions *in vivo* for non-biased identification of binding molecules (Fig. 1). We showed that enChIP using 3xFN-Tel-TAL is able to isolate telomeres (Fig. 2). Using enChIP-MS, we detected known and novel proteins interacting with telomeres (Table 1, Supplemental Table 1 and Fig. 3). Detected telomere-binding proteins showed a variety of localization patterns with telomeres (Fig. 3). For example, DDX54 showed high frequency of co-localization with telomeres, suggesting that it functions mainly at telomeres. In contrast, relatively small fractions of KDM5C and POLA1 co-localized with telomeres, suggesting that they function at telomeres as well as other sites in the nucleus. In this regard, U2OS cells used in the imaging analysis lack telomerase activity and maintain telomere DNA via homologous recombination (alternative lengthening of telomeres (ALT))^22^. Therefore, it would be interesting to examine localization of detected proteins in telomerase-positive cells. Nevertheless, enChIP-MS could detect a wide variety of telomere-interacting proteins, which is beneficial for comprehensive understanding of telomere biology. It would also be an interesting future issue to investigate the functions of newly identified telomere-binding proteins in mammalian telomeres in telomerase-positive and negative cells.

Furthermore, telomerase RNA associated with telomeres was detected by enChIP-RT-PCR (Fig. 4). Combination of enChIP with microarray analysis (enChIP-chip) or RNA-Seq analysis (enChIP-RNA-Seq) would enable us to perform non-biased search for RNA species associated with a given genomic region. In fact, we have identified a list of telomere-binding RNA species by enChIP-RNA-Seq analysis (T.F. and H.F., unpublished data). enChIP can also be combined with next generation sequencing (enChIP-Seq) to detect interactions between genomic regions.

In addition to TAL proteins, other engineered DNA-binding molecules such as zinc-finger proteins^6^ and the CRISPR system consisting of dCas9 and gRNA^8^ can be used for enChIP. In fact, in the concurrent work we could successfully isolate a single-copy locus by enChIP using dCas9 plus gRNA and identify associated proteins by MS^5^.

enChIP is a technology related to iChIP we developed recently^2^,^4^. In contrast to iChIP, enChIP does not require insertion of recognition sequences of exogenous DNA-binding proteins such as LexA. Therefore, the isolation procedure of enChIP is much more straightforward than that of iChIP. On the other hand, enChIP cannot distinguish two alleles if the target genomic region is in autosomes, whereas iChIP can differentially isolate genomic regions in a specific allele. Consequently, if the genome function is regulated in an allele-specific manner, eg. in genomic imprinting, iChIP would be the method of choice. Thus, enChIP and iChIP are complimentary approaches.

Recently, Kingston's group developed proteomics of isolated chromatin (PICh) as a novel technique to isolate specific genomic regions retaining molecular interaction^23^. In PICh specific biotinylated nucleic acid probes hybridizing target genomic regions and streptavidin beads are used to isolate the regions. Telomere-associated proteins were identified by PICh. However, it has not been shown whether PICh can be used for identification of RNA species associated with specific genomic regions. Since nucleic acid probes used in PICh can hybridize with not only genomic DNA but also RNA, careful analysis would be required to confirm if the detected RNA is associated with the target genomic regions or the RNA directly hybridizes with the probe. In this regard, RNA detected by enChIP is basically interacting with the target genomic regions. Therefore, detection of RNA associated with specific genomic regions can be more easily done by enChIP.

In summary, we isolated telomeres by enChIP using a TAL protein. enChIP-MS and enChIP-RT-PCR could successfully identified telomere-associated proteins and RNA, respectively. Thus, enChIP using TAL proteins would be a useful tool for biochemical analysis of specific genomic regions of interest.

## Methods

### Cell culture

Ba/F3-derived cells were maintained in RPMI-1640 (Wako) supplemented with 10% fetal calf serum (FCS), 10 mM HEPES (pH 7.2), 1 × non-essential amino acid, 1 mM sodium pyruvate, 5 nM 2-mercaptoethanol, and 1 ng/ml interleukin-3. U2OS-derived cells were maintained in DMEM (Nissui) supplemented with 10% FCS.

### Plasmids

The plasmid encoding the NLS-fused Telomere-TAL protein (Tel-TAL) recognizing a 19-mer telomere repeat (TAGGG*TTAGGG*TTAGGG*TT*) (the Tel-TAL cloning plasmid) was generated by Life Technologies. To construct 3xFN-Tel-TAL/pCMV-7.1, the Tel-TAL cloning plasmid was cleaved with *Not* I, blunted, and subsequently cleaved with *Pme* I to obtain the coding sequence of Tel-TAL. The coding sequence of Tel-TAL was inserted into the p3XFLAG-CMV-7.1 vector (Sigma-Aldrich). To construct 3xFN-Tel-TAL/pMXs-puro and 3xFN-Tel-TAL/pMXs-neo, 3xFN-Tel-TAL/pCMV-7.1 was cleaved with *Sac* I, blunted and subsequently cleaved with *Sma* I to obtain the coding sequence of 3xFN-Tel-TAL. The coding sequence of 3xFN-Tel-TAL was inserted into the pMXs-puro or pMXs-neo retroviral vector, which was constructed by replacing the GFP reporter cassette of pMXs-IG^24^ with puromycin or neomycin resistance gene of pMX-puro or pMX-neo^25^, respectively. Expression vectors of Halo-tagged proteins were purchased from Promega.

### DNAP assay

DNAP assay was performed as described previously^3^. Briefly, nuclear extracts were prepared from 293T cells transfected with 3xFN-Tel-TAL/pMXs-neo with NE-PER Nuclear and Cytoplasmic Extraction Reagents (Thermo Fisher). Nuclear extracts (5 μg) were incubated with the biotinylated telomere repeats (annealed double-strand oligo nucleotides of 5′- Biotin - TTAGGGTTAGGGTTAGGGTTAGGGTTAGGGTTAGGGTTAG -3′ and 5′- CTAACCCTAACCCTAACCCTAACCCTAACCCTAACCCTAA -3′) or control *IRF-1* promoter sequence (annealed double-strand oligo nucleotides of 5′- Biotin - CCGAGTGGGCCAATGGGCGCGCAGGAGCGGCGCGGCGGGG -3′ and 5′- CCCCGCCGCGCCGCTCCTGCGCGCCCATTGGCCCACTCGG -3′) (1 μg) and Poly dI-dC (15 μg) in 500 μl of DNAP Buffer (20 mM HEPES pH 7.5, 80 mM KCl, 1 mM MgCl_2_, 1 mM EDTA, 0.1% Triton X-100, 10% glycerol, 0.5 mM DTT) for 1 h at 4°C. Streptavidin-coated Dynabeads M-280 (30 μl, Invitrogen) was added into the reaction mixture and incubated for 1 h at 4°C. After washing with DNAP Buffer, the precipitants were subjected to SDS-PAGE and immunoblot analysis with anti-FLAG M2 Ab (Sigma-Aldrich).

### Establishment of cell lines stably expressing 3xFN-Tel-TAL

3xFN-Tel-TAL or control 3xFNLDD^11^ was transduced into Ba/F3 cells. Cells expressing 3xFN-Tel-TAL or 3xFNLDD were selected in puromycin-containing media.

### Flowcytometry

Cells were intracellularly stained with fluorescein isothiocyanate (FITC)-conjugated anti-FLAG M2 Ab (Sigma-Aldrich) using the Fixation/Permeabilization and Permeabilization buffer set (eBioscience). Flowcytometric analysis was performed on FACS Calibur (BD Biosciences), and data was analyzed with FlowJo software (TreeStar).

### enChIP-Southern blot analysis

Cells (2 × 10^7^) were fixed with 1% formaldehyde at 37°C for 5 min. The chromatin fraction was extracted and fragmented by sonication (the average length of fragments was about 2 kb) as described previously^3^. The sonicated chromatin in Sonication Buffer with 1% Triton X-100 was pre-cleared with 15 μg of normal mouse IgG (Santa Cruz Biotechnology) conjugated to 150 μl of Dynabeads-Protein G (Invitrogen) and subsequently incubated with 15 μg of anti-FLAG M2 Ab (Sigma-Aldrich) conjugated to 150 μl of Dynabeads-Protein G at 4°C overnight. The Dynabeads were washed twice each with 1 ml of Low Salt Wash Buffer (20 mM Tris pH 8.0, 150 mM NaCl, 2 mM EDTA, 1% TritonX-100, 0.1% SDS), High Salt Wash Buffer (20 mM Tris pH 8.0, 500 mM NaCl, 2 mM EDTA, 1% TritonX-100, 0.1% SDS), LiCl Wash Buffer (10 mM Tris pH 8.0, 250 mM LiCl, 1 mM EDTA, 0.5% IGEPAL-CA630, 0.5% sodium deoxycholate), and TE Buffer (10 mM Tris pH 8.0, 1 mM EDTA). The Dynabeads were suspended in 285 μl of TE and 12 μl of 5 M NaCl and incubated at 65°C overnight for reverse crosslink. After RNase A treatment at 37°C for 1 h, Proteinase K treatment was done at 45°C for 2 h. Subsequently, DNA was purified by phenol/chloroform treatment. Southern blot analysis of telomere DNA was performed with Telo TAGGG Telomere Length Assay (Roche) according to the manufacturer's instructions. Hybridization signal was detected with ImageQuant LAS 4000 mini (Fuji Film) and quantified with Multi Gauge software (Fuji Film).

### enChIP-MS

For the enChIP-MS analysis, the enChIP procedure was performed as described for enChIP-Southern blot analysis with 4 × 10^7^ cells, 30 μg of Abs and 300 μg of Dynabeads-Protein G. TBS Buffer (50 mM Tris pH 7.5, 150 mM NaCl) with 0.1% IGEPAL CA-630 was used instead of TE in the final step of washing procedure. The immunoprecipitants were eluted by incubating with 200 μl of 3xFLAG peptide (Sigma-Aldrich) (500 μg/ml) in TBS with 0.1% IGEPAL CA-630 at 37°C for 20 min. The eluted samples were precipitated by isopropanol. The precipitants were suspended in 40 μl of 2 × Sample buffer, incubated at 100°C for 30 min for reverse-crosslinking and denaturation of proteins, and subjected to SDS-PAGE. The proteins were visualized by Coomassie Brilliant Blue staining. Protein bands were excised and analyzed using a nanoLC-MS/MS system, composed of LTQ Orbitrap Velos (Thermo Fisher Scientific) coupled with nanoLC (Advance, Michrom BioResources) and HTC-PAL autosampler (CTC Analytics) at DNA-chip Development Center for Infectious Diseases (RIMD, Osaka University). Detailed conditions of MS analysis are described in Supplemental Methods.

### Imaging analysis

U2OS cells were transfected with the expression vector of DsRed-TRF1. Cells were fixed with 4% paraformaldehyde 24 h post transfection. The fixed cells were sequentially incubated with Abs against the indicated proteins and AlexaFluor 488 conjugated secondary Ab, and subjected to immunofluorescence microscopic analysis. Thirty cells expressing DsRed-TRF1 were randomly picked up and the frequency of signals overlapping TRF1 signals were counted and shown as graphs. Abs used in this study are: anti-KDM5C (Abcam, ab34717), anti-POLA1 (Sigma-Aldrich, HPA002947), anti-GNL3L (Sigma-Aldrich, HPA036314), anti-DDX54 (Santa Cruz Biotechnology, sc-101021), and anti-CTBP1 (BD Biosciences, 612042). For detection of POLA1 and DDX54, cells were permeabilized with PBS containing 0.2% Triton X-100 for 2 min before fixation. For detection of Halo-tagged proteins, U2OS cells were transfected with the expression vector of ECFP-TRF2 together with those of Halo-tagged proteins. Cells were treated with TMR ligand (5 μM) for 15 min at 37°C 23 h post transfection and then fixed with 4% paraformaldehyde. Thirty cells expressing both ECFP-TRF2 and Halo-tagged proteins were randomly picked up and analyzed as described above.

### enChIP-RT-PCR

For the enChIP-RT-PCR analysis, the enChIP procedure was performed as described for enChIP-Southern blot analysis with 5 U/ml of rRNasin Plus (Promega) in all the buffer solution except for Sonication Buffer in which 40 U/ml of rRNasin Plus was added. The Dynabeads were suspended in 285 μl of TE and 12 μl of 5 M NaCl and incubated at 65°C overnight for reverse crosslink. After Proteinase K treatment at 45°C for 2 h, RNA was purified with Isogen II (Nippon Gene). After treatment with RQ1 RNase-free DNase (Promega), samples were subjected to RT-PCR analysis using TITANIUM One-Step RT-PCR Kit (Clontech). PCR cycles were as follows: heating at 50°C for 1 h followed by 94°C for 5 min; 43 cycles of 94°C for 30 sec, 60°C for 30 sec and 68°C for 1 min; an additional incubation at 68°C for 2 min. The primers used in this experiment were mTR 3-RT (#27234) (5′-CCGGCGCCCCGCGGCTGACAGAG-3′) (0.4 μM), mTR 5-b (#27235) (5′-GCTGTGGGTTCTGGTCTTTTGTTC-3′) (0.9 μM) and mTR 3 (#27236) (5′-GCGGCAGCGGAGTCCTAAG-3′) (0.9 μM) described previously^26^.

## Author Contributions

T.F. and H.F. conceived the enChIP method, designed and performed experiements, and wrote the manuscript; Y.A., J.O. and R.O. performed imaging analysis and prepared Figure 3 and Supplemental Figure 6; Y.T. and K.S. performed mass spectrometry; H.F. directed and supervised the study. All authors reviewed the manuscript.

## Supplementary Material

Supplementary Information Supplementary Info

## Figures and Tables

**Table 1:** Examples of proteins detected in enChIP-MS

Mammalian telomere-binding proteins	PML, RPA, CDK1, PARP1, PCBP1
Telomere-binding proteins in yeast or other organisms	IMP4
Proteins interacting with telomere-binding proteins [associated telomere-binding protein]	DNA polymerase α (POLA1) [Cdc13p], ARMC6 [TRF2], CTBP1 [FoxP2-POT1], exportin-5 [TERT], GNL3L [TRF1], exportin-1 [TERT], 14-3-3 [TERT]
Proteins localizing to heterochromatin	BEND3
Proteins regulating epigenetic marks	KDM5C
Proteins whose mutations affect telomere function	DNA polymerase α (POLA1), HAT1, Nup133, CDK7, DPOE1, PRDX1, TYSY, glutamate-cysteine ligase, glutaredoxin, SMRC1

All of identified proteins and references are shown in Supplemental Table 1.
