# Supplementary material for: Identification of telomere-associated molecules by engineered DNA-binding molecule-mediated chromatin immunoprecipitation (enChIP)
Source: Sci Rep. 2013 Nov 8;3:3171. doi: 10.1038/srep03171 (PMC3821016; doi:10.1038/srep03171)
Supplement: Supplementary Information — Supplementary Info [file srep03171-s1.pdf]

Supplemental Materials

Identification of telomere-associated molecules by engineered DNA-binding molecule-mediated chromatin immunoprecipitation (enChIP)

Toshitsugu Fujita, Yoshinori Asano, Junko Ohtsuka, Yoko Takada, Kazunobu Saito, Rieko Ohki, Hodaka Fujii

Supplemental Methods

Supplemental Figure Legends

Supplemental References

Supplemental Figures 1 - 7

Supplemental Table 1

Supplemental Methods

enChIP-PCR. For detection of γ-satellite sequence, enChIP was performed as described in enChIP-Southern blot analysis except that ChIP DNA Clean & Concentrator (Zyme Research) was used for purification of DNA. The purified DNA was used for PCR with AmpliTaq Gold 360 Master Mix (Applied Biosystems). PCR cycles were as follows: denaturing at 95°C for 10 min; 17 cycles of 95°C for 15 sec, 60°C for 1 min. The primers used in this experiment are 5'-gcgagaaaactgaaaatcac-3' and 5'-tcaagtcgtcaagtggatg-3'[1]. The PCR amplicons were subjected to electrophoresis in 2% agarose gel and visualized in Gel Doc XR (Bio-Rad). The intensity of visualized bands was analyzed using Scion Imaging Software (Scion Corporation).

Identification of proteins by LC-MS/MS. After staining with Coumassie Brilliant Blue, the each lane was cut into 5 slices. Each slice was digested with trypsin, and the obtained peptides were dried and then dissolved in 0.1% trifluoroacetic acid, 2% acetonitrile prior to LC-MS/MS analysis. Peptides were analysed using a nanoLC-MS/MS system, composed of an LTQ Orbitrap Velos (Thermo Fisher Scientific) coupled with a nanoLC (Advance, Michrom BioResources) and an HTC-PAL autosampler (CTC Analytics). Peptide separation was carried out using the C18 reversed phase analytical column (0.1 mm ID x 15 cm, 3 μm resin, L-column Micro; CELI). The mobile phases consisted of 0.1% formic acid and 100% acetonitrile. Peptides were eluted by a gradient of 5–35% acetonitrile for 105 min at a flow rate of 500 nl/min. CID spectra were acquired automatically in the data-dependent scan mode with the dynamic exclusion option. Full MS was obtained by Orbitrap in the range of m/z 300–1,500 with resolution 30,000. The nine most intense precursor ions in the full MS spectra were selected for subsequent MS/MS analysis

in an ion trap with the automated gain control mode. The lock mass function was activated to minimize mass error during analysis.

Database searching. Tandem mass spectra were extracted, then charge state was deconvoluted and deisotoped by Proteome Discoverer version 1.2. All MS/MS samples were analyzed using Mascot (Matrix Science, London, UK; version 2.4.1) and X! Tandem (The GPM, thegpm.org; version CYCLONE (2010.12.01.1)). X! Tandem was set up to search the SwissProt_2012 database (535248 entries) also assuming the digestion enzyme as trypsin. Mascot was set up to search the SwissProt_2013_08 database (selected for Mus musculus, 16625 entries) assuming the digestion enzyme as trypsin. Mascot and X! Tandem were searched with a fragment ion mass tolerance of 0.80 Da and a parent ion tolerance of 10.0 PPM. Iodoacetamide derivative of cysteine was specified in Mascot and X! Tandem as a fixed modification. S-carbamoylmethylcysteine cyclization (N-terminus) of the n-terminus, oxidation of methionine and acetylation of the n-terminus were specified in Mascot and X! Tandem as variable modifications.

Criteria for protein identification. Scaffold (version Scaffold_3.4.5, Proteome Software Inc., Portland, OR) was used to validate MS/MS based peptide and protein identifications. Peptide identifications were accepted if they could be established at greater than 95.0% probability as specified by the Peptide Prophet algorithm[2]. Protein identifications were accepted if they could be established at greater than 99.0% probability and contained at least 2 identified peptides. Protein probabilities were assigned by the Protein Prophet algorithm[3]. These thresholds resulted in a protein false discovery rate (FDR) of < 0.1% as calculated by Scaffold. Proteins that

contained similar peptides and could not be differentiated based on MS/MS analysis alone were grouped to satisfy the principles of parsimony. MS data were converted using PRIDE Converter 2 Tool Suite (version 2.0.19)[4] and deposited in the PRIDE database (http://www.ebi.ac.uk/pride)[5-7] under accession numbers [31251-31256].

Quantification using spectral counting. Label-free quantification of relative protein abundance was performed by spectral counting[8] using all spectra matched to a peptide sequence. Relative protein abundance was calculated on the basis of the unweighted spectral count assigned to each identified protein by Scaffold. The unweighted spectral count includes spectra matched to peptides shared between multiple proteins if there is independent evidence that these proteins are present. To normalize the data, spectral counts were expressed as a percentage of the total number of spectra expressed as a percentage of the total number of spectra observed in the entire sample. sd and p-values were calculated from three biological replicates. p-values were calculated by t-test.

Supplemental Figure Legends

Supplemental Figure 1. Nucleotide and amino acid sequences of 3xFN-Tel-TAL. Sequences of 3xFLAG-tag, V5 epitope-tag and NLS are highlighted.

Supplemental Figure 2. The full-length blot of Figure 2b including molecular size marker.

Supplemental Figure 3. The full-length blot of Figure 2d including molecular size marker.

Supplemental Figure 4. The amounts of γ-satellite repeats in enChIP samples. γ-satellite repeats in enChIP samples were quantified by PCR.

Supplemental Figure 5. SDS-PAGE and CBB staining. The stained regions were divided into 5 parts (2-4 mm height each), excised, and subjected to in-gel tryptic digestion. The digested peptides were analyzed in LC-MS/MS.

Supplemental Figure 6. Localization of candidate proteins at telomeres. U2OS cells were transfected with the expression vector of ECFP-TRF2 together with those of Halo-tagged proteins. Cells were treated with TMR ligand (5 μM) for 15 min at 37°C 23 h post transfection, fixed with 4% paraformaldehyde, and subjected to immunofluorescence microscopy. Thirty cells expressing both ECFP-TRF2 and Halo-tagged proteins were randomly picked up and analyzed as Figure 2.

Supplemental Figure 7. The full-length gel image of Figure 4.

Supplemental References

1. Kishi, Y., Kondo, S. & Gotoh, Y. Transcriptional activation of mouse major satellite regions during neuronal differentiation. *Cell Struct. Funct.* **37**, 101-110 (2012).
2. Keller, A., Nesvizhskii, A.I., Kolker, E. & Aebersold, R. Empirical statistical model to estimate the accuracy of peptide identifications made by MS/MS and database search. *Anal Chem* **74**, 5383-5392 (2002).
3. Nesvizhskii, A.I., Keller, A., Kolker, E. & Aebersold, R. A statistical model for identifying proteins by tandem mass spectrometry. *Anal Chem* **75**, 4646-4658 (2003).
4. Barsnes, H., Vizcaíno, J.A., Eidhammer, I. & Martens, L. PRIDE Converter: Making proteomics data-sharing easy. *Nat. Biotechnol.* **27**, 598-599 (2009).
5. Vizcaíno, J.A. et al. The Proteomics Identifications database: 2010 update. *Nucleic Acids Res.* **38**, D736-D742 (2010).
6. Old, W.M. et al. Comparison of label-free methods for quantifying human proteins by shotgun proteomics. *Mol. Cell. Proteomics* **4**, 1487-1502 (2005).
7. Zybailov, B., Coleman, M.K., Florens, L. & Washburn, M.P. Correlation of relative abundance ratios derived from peptide ion chromatograms and spectrum counting for quantitative proteomic analysis using stable isotope labeling. *Anal. Chem.* **77**, 6218-6224 (2005).
8. Liu, H.B., Sadygov, R.G. & Yates, J.R. A model for random sampling and estimation of relative protein abundance in shotgun proteomics. *Anal. Chem.* **76**, 4193-4201 (2004).

Supplemental Figure 1

```
                                   3xFLAG-tag
   1 M   D   Y   K   D   H   D   G   D   Y   K   D   H   D   I   D   Y   K   D   D   D   D   K   L   A   A   G   R   A   T    30
   1 ATG GAC TAC AAA GAC CAT GAC GGT GAT TAT AAA GAT CAT GAC ATC GAT TAC AAG GAT GAC GAT GAC AAG CTT GCG GCC GGC CGC GCC ACC  90
                 V5 epitope-tag                                              NLS
  31 M   G   K   P   I   P   N   P   L   L   G   L   D   S   T   G   G   M   A   P   K   K   K   R   K   V   D   G   G   V    60
  91 ATG GGA AAA CCT ATT CCT AAT CCT CTG CTG GGC CTG GAT TCT ACC GGA GGC ATG GCC CCT AAG AAA AAG CGG AAG GTG GAC GGC GGA GTG  180

  61 D   L   R   T   L   G   Y   S   Q   Q   Q   Q   E   K   I   K   P   K   V   R   S   T   V   A   Q   H   H   E   A   L    90
 181 GAC CTG AGA ACA CTG GGA TAT TCT CAG CAG CAG CAG GAG AAG ATC AAG CCC AAG GTG AGA TCC ACA GTG GCC CAG CAC CAC GAA GCC CTG  270

  91 V   G   H   G   F   T   H   A   H   I   V   A   L   S   Q   H   P   A   A   L   G   T   V   A   V   K   Y   Q   D   M    120
 271 GTG GGA CAC GGA TTT ACA CAC GCC CAC ATT GTG GCC CTG TCT CAG CAC CCT GCC GCC CTG GGA ACA GTG GCC GTG AAA TAT CAG GAT ATG  360

 121 I   A   A   L   P   E   A   T   H   E   A   I   V   G   V   G   K   Q   W   S   G   A   R   A   L   E   A   L   L   T    150
 361 ATT GCC GCC CTG CCT GAG GCC ACA CAC GAA GCC ATT GTG GGA GTG GGA AAA CAG TGG TCT GGA GCC AGA GCC CTG GAA GCC CTG CTG ACA  450

 151 V   A   G   E   L   R   G   P   P   L   Q   L   D   T   G   Q   L   L   K   I   A   K   R   G   G   V   T   A   V   E    180
 451 GTG GCC GGA GAA CTG AGA GGA CCT CCT CTG CAG CTG GAT ACA GGA CAG CTG CTG AAG ATT GCC AAA AGG GGC GGA GTG ACC GCG GTG GAA  540

 181 A   V   H   A   W   R   N   A   L   T   G   A   P   L   N   L   T   P   E   Q   V   V   A   I   A   S   N   I   G   G    210
 541 GCC GTG CAC GCC TGG AGA AAT GCC CTG ACA GGA GCC CCT CTG AAC CTG ACC CCC GAA CAG GTG GTG GCC ATT GCC AGC AAC ATC GGC GGC  630

 211 K   Q   A   L   E   T   V   Q   R   L   L   P   V   L   C   Q   A   H   G   L   T   P   E   Q   V   V   A   I   A   S    240
 631 AAG CAG GCC CTG GAA ACC GTG CAG AGA CTG CTG CCC GTG CTG TGC CAG GCC CAT GGC CTG ACA CCT GAA CAG GTG GTG GCT ATC GCC TCT  720

 241 N   N   G   G   K   Q   A   L   E   T   V   Q   R   L   L   P   V   L   C   Q   A   H   G   L   T   P   E   Q   V   V    270
 721 AAC AAC GGA GGA AAA CAG GCT CTG GAA ACA GTG CAG CGG CTG CTG CCT GTG CTG TGT CAG GCT CAC GGC TTG ACT CCA GAA CAG GTG GTG  810

 271 A   I   A   S   N   N   G   G   K   Q   A   L   E   T   V   Q   R   L   L   P   V   L   C   Q   A   H   G   L   T   P    300
 811 GCT ATT GCT TCC AAC AAC GGG GGG AAA CAG GCC CTG GAA ACT GTG CAG CGC CTG CTG CCA GTG CTG TGC CAG GCT CAC GGA CTG ACC CCC  900

 301 E   Q   V   V   A   I   A   S   N   N   G   G   K   Q   A   L   E   T   V   Q   R   L   L   P   V   L   C   Q   A   H    330
 901 GAA CAG GTG GTG GCC ATT GCC AGC AAC AAC GGC GGC AAG CAG GCC CTG GAA ACC GTG CAG AGA CTG CTG CCC GTG CTG TGC CAG GCC CAT  990

 331 G   L   T   P   E   Q   V   V   A   I   A   S   N   G   G   G   K   Q   A   L   E   T   V   Q   R   L   L   P   V   L    360
 991 GGC CTG ACA CCT GAA CAG GTG GTG GCT ATC GCC TCT AAC GGC GGA GGA AAA CAG GCT CTG GAA ACA GTG CAG CGG CTG CTG CCT GTG CTG  1080

 361 C   Q   A   H   G   L   T   P   E   Q   V   V   A   I   A   S   N   G   G   G   K   Q   A   L   E   T   V   Q   R   L    390
1081 TGT CAG GCT CAC GGC TTG ACT CCA GAA CAG GTG GTG GCT ATT GCT TCC AAC GGC GGG GGG AAA CAG GCC CTG GAA ACT GTG CAG CGC CTG  1170

 391 L   P   V   L   C   Q   A   H   G   L   T   P   E   Q   V   V   A   I   A   S   N   I   G   G   K   Q   A   L   E   T    420
1171 CTG CCA GTG CTG TGC CAG GCT CAC GGG CTG ACC CCC GAA CAG GTG GTG GCC ATT GCC AGC AAC ATC GGC GGC AAG CAG GCC CTG GAA ACC  1260

 421 V   Q   R   L   L   P   V   L   C   Q   A   H   G   L   T   P   E   Q   V   V   A   I   A   S   N   N   G   G   K   Q    450
1261 GTG CAG AGA CTG CTG CCC GTG CTG TGC CAG GCC CAT GGC CTG ACA CCT GAA CAG GTG GTG GCT ATC GCC TCT AAC AAC GGA GGA AAA CAG  1350

 451 A   L   E   T   V   Q   R   L   L   P   V   L   C   Q   A   H   G   L   T   P   E   Q   V   V   A   I   A   S   N   N    480
1351 GCT CTG GAA ACA GTG CAG CGG CTG CTG CCT GTG CTG TGT CAG GCT CAC GGC TTG ACT CCA GAA CAG GTG GTG GCT ATT GCT TCC AAC AAC  1440

 481 G   G   K   Q   A   L   E   T   V   Q   R   L   L   P   V   L   C   Q   A   H   G   L   T   P   E   Q   V   V   A   I    510
1441 GGG GGG AAA CAG GCC CTG GAA ACT GTG CAG CGC CTG CTG CCA GTG CTG TGC CAG GCT CAC GGC CTC ACT CCC GAA CAG GTG GTG GCC ATT  1530

 511 A   S   N   N   G   G   K   Q   A   L   E   T   V   Q   R   L   L   P   V   L   C   Q   A   H   G   L   T   P   E   Q    540
1531 GCC AGC AAC AAC GGC GGC AAG CAG GCC CTG GAA ACC GTG CAG AGA CTG CTG CCC GTG CTG TGC CAG GCC CAT GGC CTG ACA CCT GAA CAG  1620

 541 V   V   A   I   A   S   N   G   G   G   K   Q   A   L   E   T   V   Q   R   L   L   P   V   L   C   Q   A   H   G   L    570
1621 GTG GTG GCT ATC GCC TCT AAC GGC GGA GGA AAA CAG GCT CTG GAA ACA GTG CAG CGG CTG CTG CCT GTG CTG TGT CAG GCT CAC GGC TTG  1710

 571 T   P   E   Q   V   V   A   I   A   S   N   G   G   G   K   Q   A   L   E   T   V   Q   R   L   L   P   V   L   C   Q    600
1711 ACT CCA GAA CAG GTG GTG GCT ATT GCT TCC AAC GGC GGG GGG AAA CAG GCC CTG GAA ACT GTG CAG CGC CTG CTG CCA GTG CTG TGC CAG  1800

 601 A   H   G   L   T   P   E   Q   V   V   A   I   A   S   N   I   G   G   K   Q   A   L   E   T   V   Q   R   L   L   P    630
1801 GCT CAC GGA CTG ACC CCC GAA CAG GTG GTG GCC ATT GCC AGC AAC ATC GGC GGC AAG CAG GCC CTG GAA ACC GTG CAG AGA CTG CTG CCC  1890

 631 V   L   C   Q   A   H   G   L   T   P   E   Q   V   V   A   I   A   S   N   N   G   G   K   Q   A   L   E   T   V   Q    660
1891 GTG CTG TGC CAG GCC CAT GGC CTG ACA CCT GAA CAG GTG GTG GCT ATC GCC TCT AAC AAC GGA GGA AAA CAA GCA CTC GAG ACA GTG CAG  1980

 661 R   L   L   P   V   L   C   Q   A   H   G   L   T   P   E   Q   V   V   A   I   A   S   N   N   G   G   K   Q   A   L    690
1981 CGG CTG CTG CCT GTG CTG TGT CAG GCT CAC GGC TTG ACT CCA GAA CAG GTG GTG GCT ATT GCT TCC AAC AAC GGG GGG AAA CAG GCC CTG  2070

 691 E   T   V   Q   R   L   L   P   V   L   C   Q   A   H   G   L   T   P   E   Q   V   V   A   I   A   S   N   N   G   G    720
2071 GAA ACT GTG CAG CGC CTG CTG CCA GTG CTG TGC CAG GCT CAC GGG CTG ACC CCC GAA CAG GTG GTG GCC ATT GCC AGC AAC AAC GGC GGC  2160

 721 K   Q   A   L   E   T   V   Q   R   L   L   P   V   L   C   Q   A   H   G   L   T   P   E   Q   V   V   A   I   A   S    750
2161 AAG CAG GCC CTG GAA ACC GTG CAG AGA CTG CTG CCC GTG CTG TGC CAG GCC CAT GGC CTG ACA CCT GAA CAG GTG GTG GCT ATC GCC TCT  2250

 751 N   G   G   G   K   Q   A   L   E   T   V   Q   R   L   L   P   V   L   C   Q   A   H   G   L   T   P   Q   Q   V   V    780
2251 AAC GGC GGA GGA AAA CAG GCT CTG GAA ACA GTG CAG CGG CTG CTG CCT GTG CTG TGT CAG GCT CAC GGC TTG ACT CCA CAG CAG GTC GTG  2340

 781 A   I   A   S   N   G   G   G   R   P   A   L   E   S   I   V   A   Q   L   S   R   P   D   P   A   L   A   A   L   T    810
2341 GCA ATT GCT AGC AAC GGC GGC GGA CGG CCC GCC CTG GAG AGC ATT GTG GCC CAG CTG TCT AGA CCT GAT CCT GCC CTG GCC GCC CTG ACA  2430

 811 N   D   H   L   V   A   L   A   C   L   G   G   R   P   A   L   D   A   V   K   K   G   L   P   H   A   P   A   L   I    840
2431 AAT GAT CAC CTG GTG GCC CTG GCC TGT CTG GGA GGC AGA CCT GCC CTG GAT GCC GTG AAA AAA GGA CTG CCT CAC GCC CCT GCC CTG ATC  2520

 841 K   R   T   N   R   R   I   P   E   R   T   S   H   R   V   A   D   H   A   Q   V   V   R   V   L   G   F   F   Q   C    870
2521 AAG AGA ACA AAT AGA AGA ATC CCC GAG CGG ACC TCT CAC AGA GTG GCC GAT CAC GCC CAG GTG GTG AGA GTG CTG GGA TTT TTT CAG TGT  2610

 871 H   S   H   P   A   Q   A   F   D   D   A   M   T   Q   F   G   M   S   R   H   G   L   L   Q   L   F   R   R   V   G    900
2611 CAC TCT CAC CCT GCC CAG GCC TTT GAT GAT GCC ATG ACA CAG TTT GGC ATG AGC AGA CAC GGA CTG CTG CAG CTG TTT AGA AGA GTG GGA  2700

 901 V   T   E   L   E   A   R   S   G   T   L   P   P   A   S   Q   R   W   D   R   I   L   Q   G   S   R   L   I   H   R    930
2701 GTG ACA GAA CTG GAG GCC AGA TCC GGA ACC CTG CCT CCT GCC TCT CAG AGA TGG GAT AGG ATT CTG CAG GGT TCC CGT TTA ATT CAT CGA  2790

 931 *   931
2791 TAG 2793
```

Supplemental Figure 2

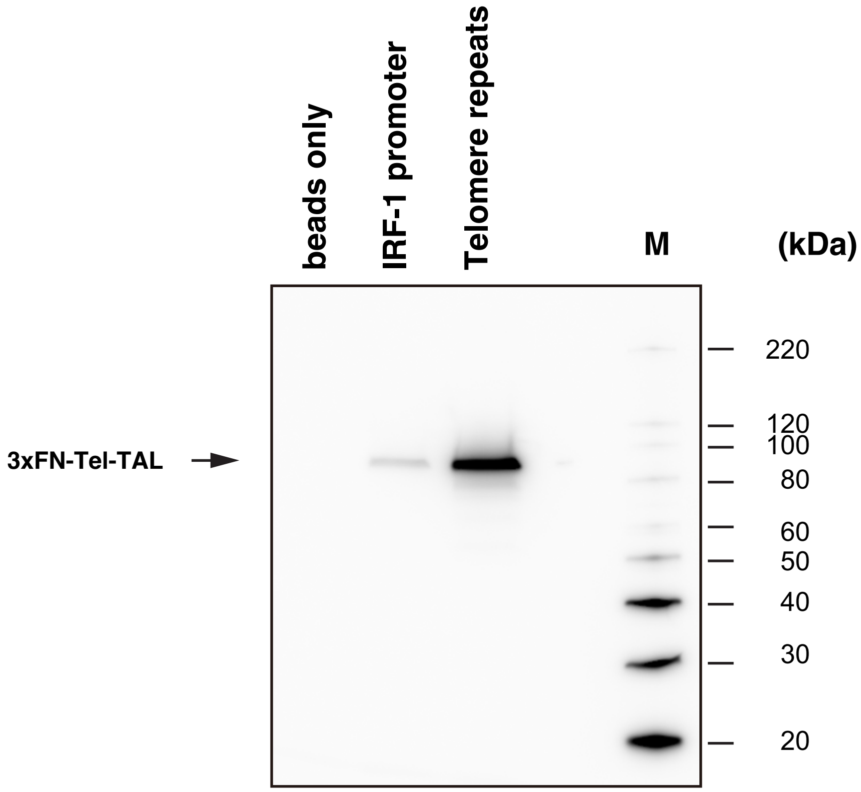

Supplemental Figure 3

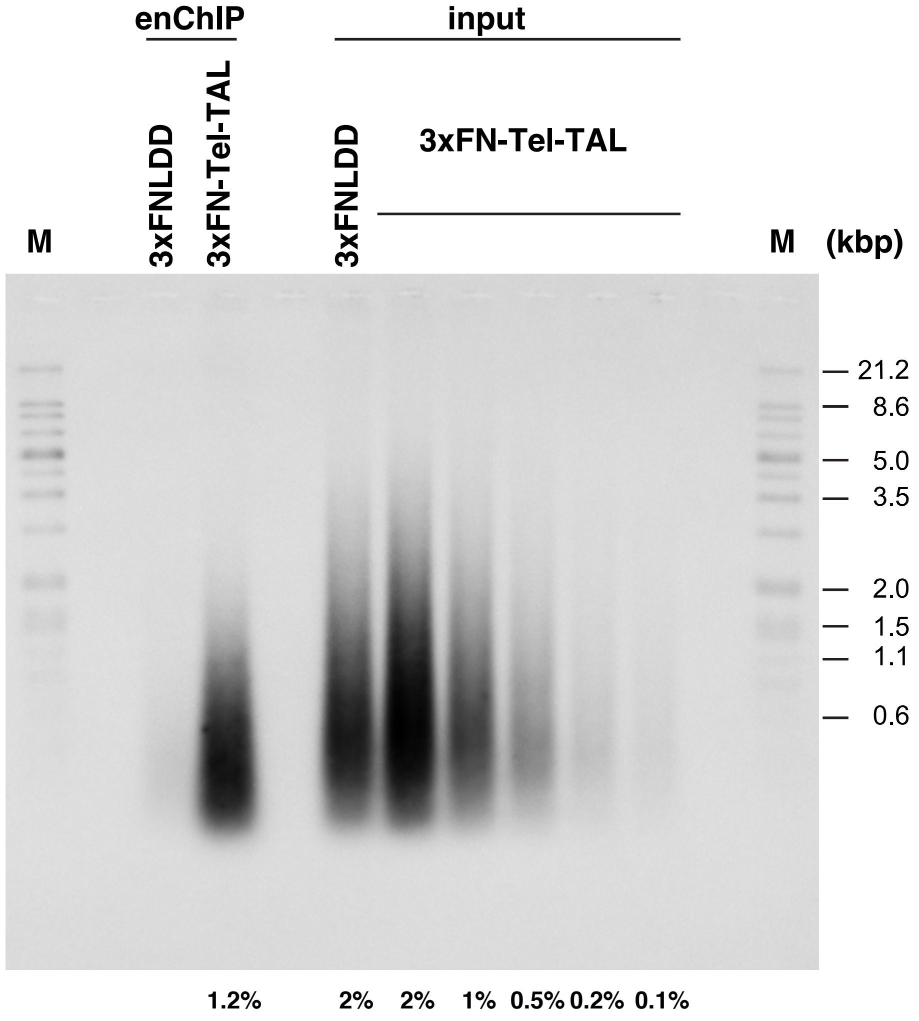

Supplemental Figure 4

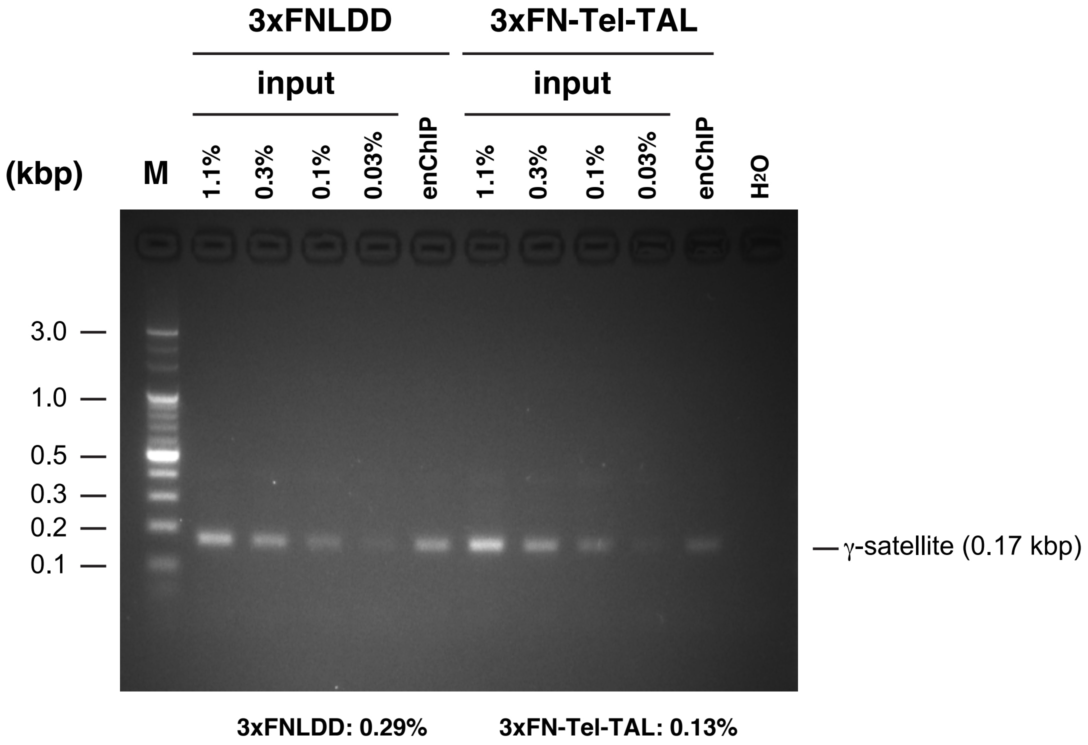

Supplemental Figure 5

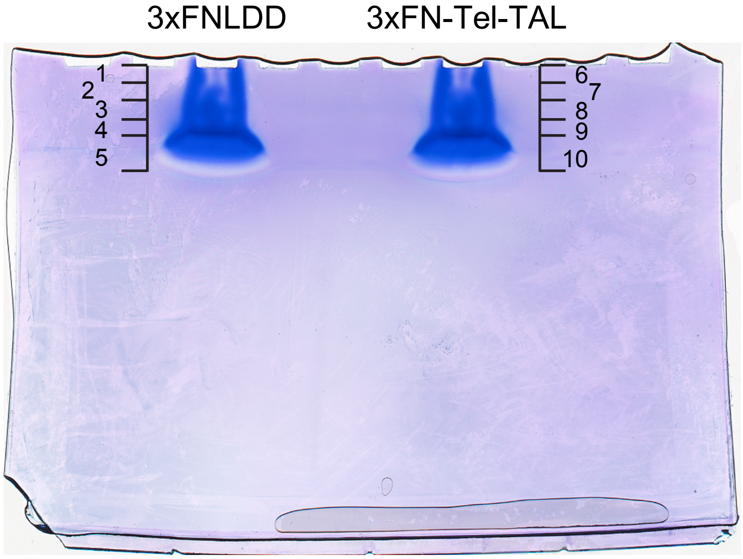

Supplemental Figure 6

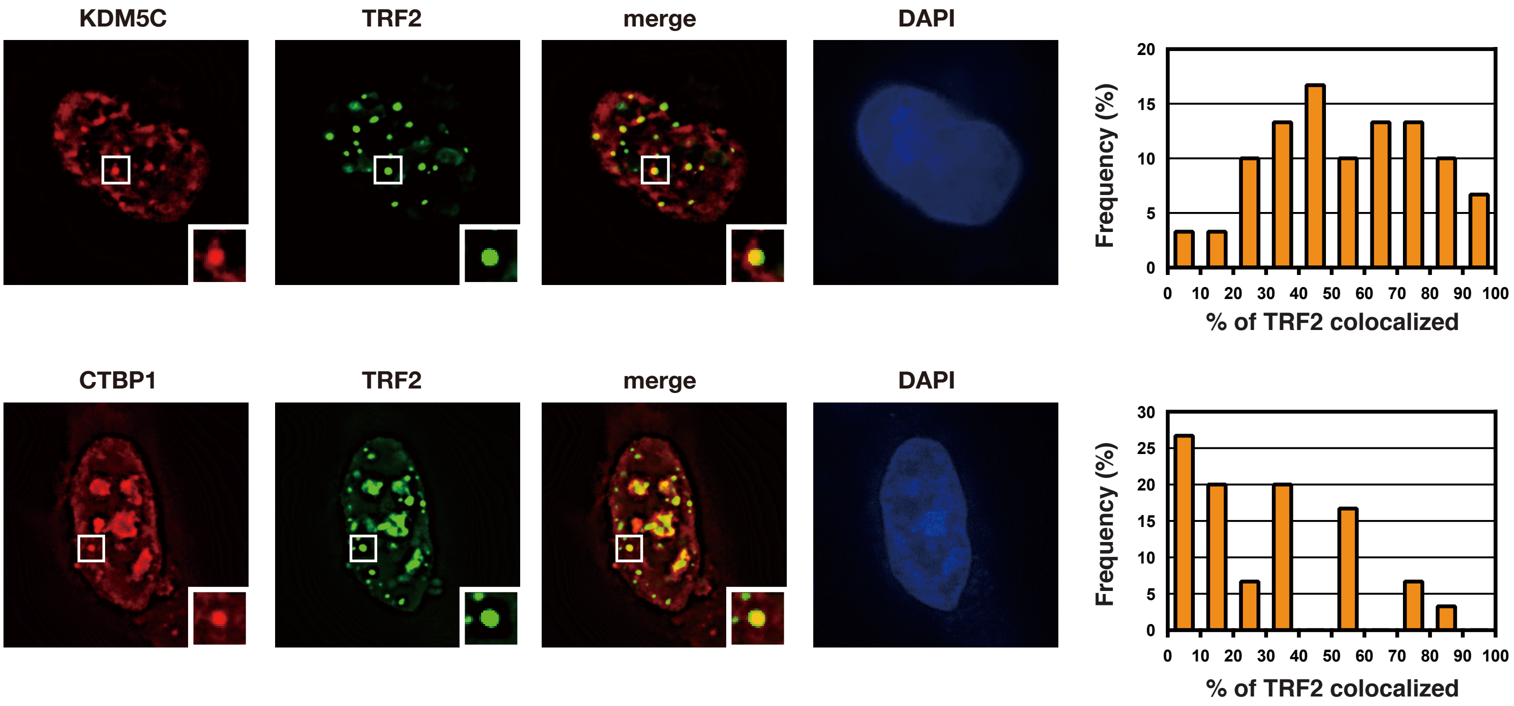

Supplemental Figure 7

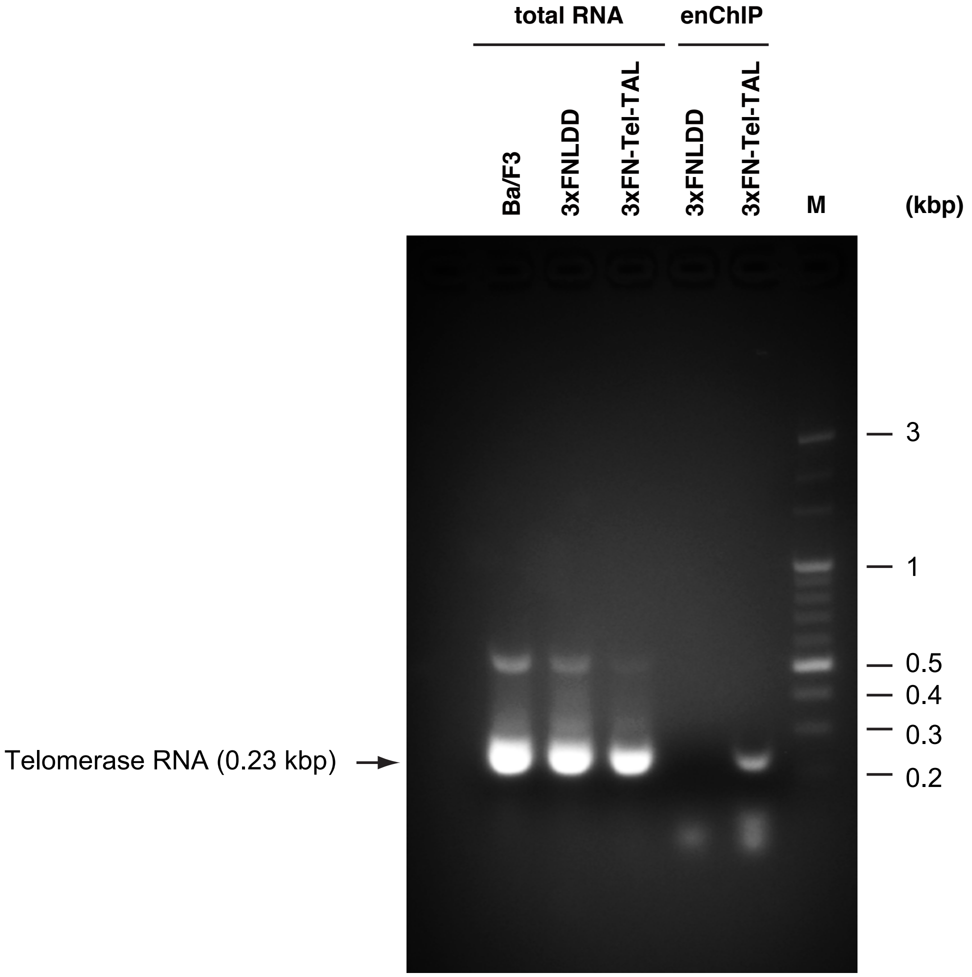

Supplemental Table 1

Red: Mammalian telomere-binding proteins
Blue: Telomere-binding in yeast and other organisms
Orange: Proteins interacting telomere-binding proteins
Purple: Proteins localizing to heterochromatin.
Green: Genetic evidence of involvement in telomere functions

				Normalized spectral abundance factor							
				Average	SD	Average	SD				
				Control	Control	Sample	Sample	ratio			
#	Identified Proteins (774)	Accession Number	Molecular Weight	3xFNLDD	3xFNLDD	3xFVN-Tel-TAL	3xFVN-Tel-TAL	3xFVN-Tel-TAL/3xFNLDD		Refs	T-Test (P-Value)
1	Avirulence protein AvrBs3 OS=Xanthomonas euvesicatoria GN=avrBs3 PE=1 SV=2	AVRB3_XANEU	122 kDa	0	0	36.93	14.1	#DIV/0!	3xFVN-Telomere-TAL		95% (0.011)
2	Zinc finger protein Helios OS=Mus musculus GN=Ikzf2 PE=1 SV=2	IKZF2_MOUSE	59 kDa	0	0	13.73	2.46	#DIV/0!			95% (0.00064)
3	26S proteasome non-ATPase regulatory subunit 5 OS=Mus musculus GN=Psmd5 PE=2 SV=4	PSMD5_MOUSE	56 kDa	0	0	12.98	2.19	#DIV/0!			95% (0.00050)
4	ATP-dependent RNA helicase A OS=Bos taurus GN=DHX9 PE=2 SV=1	DHX9_BOVIN	142 kDa	0	0	11.92	20.64	#DIV/0!			0% (0.37)
5	14-3-3 protein gamma OS=Mus musculus GN=Ywhag PE=1 SV=2	1433G_MOUSE	28 kDa	0	0	8.7	8.17	#DIV/0!	14-3-3 θ and ζ bind to TERT.	Seimiya et al., EMBO J (2000) 11, 2652-2661.	0% (0.14)
6	DnaJ homolog subfamily A member 1 OS=Mus musculus GN=Dnaja1 PE=1 SV=1	DNJA1_MOUSE	45 kDa	0	0	8.46	2.71	#DIV/0!			95% (0.0057)
7	Tyrosine-protein kinase BTK OS=Mus musculus GN=Btk PE=1 SV=4	BTK_MOUSE	76 kDa	0	0	8.45	5.04	#DIV/0!			95% (0.044)
8	Guanine nucleotide-binding protein G(I)/G(S)/G(T) subunit beta-2 OS=Mus musculus GN=Gnb2 PE=1 SV=3	GBB2_MOUSE	37 kDa	0	0	6	5.27	#DIV/0!			0% (0.12)
9	Anaphase-promoting complex subunit 1 OS=Mus musculus GN=Anapc1 PE=1 SV=2	APC1_MOUSE	216 kDa	0	0	5.6	3.13	#DIV/0!			95% (0.036)
10	Zinc finger protein ZFPM1 OS=Mus musculus GN=Zfpm1 PE=1 SV=1	FOG1_MOUSE	106 kDa	0	0	5.42	1.68	#DIV/0!			95% (0.0050)
11	14-3-3 protein eta OS=Mus musculus GN=Ywhah PE=1 SV=2	1433F_MOUSE	28 kDa	0	0	5.34	4.69	#DIV/0!	14-3-3 θ and ζ bind to TERT.	Seimiya et al., EMBO J (2000) 11, 2652-2661.	0% (0.12)
12	Regulator of nonsense transcripts 1 OS=Mus musculus GN=Upf1 PE=1 SV=2	RENT1_MOUSE	124 kDa	0	0	5.16	7.43	#DIV/0!			0% (0.30)
13	Histone deacetylase 2 OS=Mus musculus GN=Hdac2 PE=1 SV=1	HDAC2_MOUSE	55 kDa	0	0	5.04	4.54	#DIV/0!			0% (0.13)
14	Proteasome subunit alpha type-4 OS=Mus musculus GN=Psma4 PE=1 SV=1	PSA4_MOUSE	29 kDa	0	0	4.99	1.64	#DIV/0!			95% (0.0062)
15	Replication factor C subunit 4 OS=Mus musculus GN=Rfc4 PE=1 SV=1	RFC4_MOUSE	40 kDa	0	0	4.54	4.14	#DIV/0!			0% (0.13)
16	Transforming protein RhoA OS=Mus musculus GN=Rhoa PE=1 SV=1	RHOA_MOUSE	22 kDa	0	0	4.24	3.77	#DIV/0!			0% (0.12)
17	Fatty acid-binding protein, epidermal OS=Mus musculus GN=Fabp5 PE=1 SV=3	FABP5_MOUSE	15 kDa	0	0	4.24	3.77	#DIV/0!			0% (0.12)
18	Importin-5 OS=Mus musculus GN=Ipo5 PE=1 SV=3	IPO5_MOUSE	124 kDa	0	0	4.12	1.3	#DIV/0!			95% (0.0054)
19	MDS1 and EVI1 complex locus protein EVI1 OS=Mus musculus GN=Mecom PE=1 SV=1	EVI1_MOUSE	117 kDa	0	0	4.1	1.93	#DIV/0!			95% (0.021)
20	Nitric oxide synthase-interacting protein OS=Mus musculus GN=Nosip PE=2 SV=1	NOSIP_MOUSE	33 kDa	0	0	3.97	1.7	#DIV/0!			95% (0.015)
21	Dihydrofolate reductase OS=Mus musculus GN=Dhfr PE=1 SV=3	DYR_MOUSE	22 kDa	0	0	3.87	3.63	#DIV/0!			0% (0.14)
22	Cell division cycle protein 23 homolog OS=Mus musculus GN=Cdc23 PE=1 SV=2	CDC23_MOUSE	69 kDa	0	0	3.64	1.26	#DIV/0!			95% (0.0074)
23	RNA polymerase II subunit A C-terminal domain phosphatase SSU72 OS=Mus musculus GN=Ssu72 PE=2 SV=1	SSU72_MOUSE	23 kDa	0	0	3.37	1.17	#DIV/0!			95% (0.0075)
24	Transcriptional repressor p66-beta OS=Mus musculus GN=Gatad2b PE=1 SV=1	P66B_MOUSE	65 kDa	0	0	3.35	0.23	#DIV/0!			95% (0.000015)
25	Transcription initiation factor IIB OS=Mus musculus GN=Gtf2b PE=1 SV=1	TF2B_MOUSE	35 kDa	0	0	3.34	1.03	#DIV/0!			95% (0.0050)
26	Histidine triad nucleotide-binding protein 1 OS=Mus musculus GN=Hint1 PE=1 SV=3	HINT1_MOUSE	14 kDa	0	0	3.2	3.16	#DIV/0!			0% (0.15)
27	Tubulin-folding cofactor B OS=Mus musculus GN=Tbcb PE=1 SV=2	TBCB_MOUSE	27 kDa	0	0	3.2	3.16	#DIV/0!			0% (0.15)
28	Myotubularin-related protein 3 OS=Homo sapiens GN=MTMR3 PE=1 SV=3	MTMR3_HUMAN	134 kDa	0	0	3.15	5.46	#DIV/0!			0% (0.37)
29	Alpha-soluble NSF attachment protein OS=Mus musculus GN=Napa PE=1 SV=1	SNAA_MOUSE	33 kDa	0	0	3.07	1.19	#DIV/0!			95% (0.011)
30	Eukaryotic translation initiation factor 4H OS=Mus musculus GN=Eif4h PE=1 SV=3	IF4H_MOUSE	27 kDa	0	0	3.03	2.79	#DIV/0!			0% (0.13)
31	Transportin-1 OS=Mus musculus GN=Tnpo1 PE=1 SV=2	TNPO1_MOUSE	102 kDa	0	0	3	3.17	#DIV/0!			0% (0.18)
32	DNA polymerase alpha subunit B OS=Mus musculus GN=Pola2 PE=1 SV=2	DPOA2_MOUSE	66 kDa	0	0	2.94	1.36	#DIV/0!	DPOLA binds to telomere protein Cdc13p in budding yeast.<br>A ts-mutant mouse cell lines show telomere defects.	Qi, Genes Dev. (2000) 14, 1777-1788.<br>Hsu, Nucleic Acids Res. (2004) 32, 511-521.<br>Nakamura, EMBO J. (2005) 25, 11073-11088.	95% (0.020)
33	Replication protein A 32 kDa subunit OS=Mus musculus GN=Rpa2 PE=1 SV=1	RFA2_MOUSE	30 kDa	0	0	2.9	2.73	#DIV/0!			0% (0.14)
34	Nuclear factor NF-kappa-B p105 subunit OS=Mus musculus GN=Nfkb1 PE=1 SV=2	NFKB1_MOUSE	106 kDa	0	0	2.9	2.73	#DIV/0!			0% (0.14)
35	Protein CBFA2T3 OS=Mus musculus GN=Cbfa2t3 PE=1 SV=2	MTG16_MOUSE	68 kDa	0	0	2.63	0.84	#DIV/0!			95% (0.0056)
36	Signal transducer and activator of transcription 5A OS=Mus musculus GN=Stat5a PE=1 SV=1	STA5A_MOUSE	91 kDa	0	0	2.6	2.33	#DIV/0!			0% (0.13)
37	Adenylyltransferase and sulfurtransferase MOCS3 OS=Mus musculus GN=Mocs3 PE=2 SV=1	MOCS3_MOUSE	49 kDa	0	0	2.6	2.33	#DIV/0!			0% (0.13)
38	C-terminal-binding protein 2 OS=Mus musculus GN=Ctbp2 PE=1 SV=2	CTBP2_MOUSE	49 kDa	0	0	2.54	2.72	#DIV/0!			0% (0.18)
39	Guanine nucleotide-binding protein-like 3-like protein OS=Mus musculus GN=Gnl3l PE=2 SV=1	GNL3L_MOUSE	65 kDa	0	0	2.5	2.22	#DIV/0!	GNL3L binds to TERT and TRF1.	Fu, D, Mol. Cell (2007) 28, 773-785.<br>Zhu, J. Cell Biol. (2009) 185, 827-839.	0% (0.12)
40	Myotubularin-related protein 3 OS=Mus musculus GN=Mtmr3 PE=1 SV=2	MTMR3_MOUSE	134 kDa	0	0	2.45	4.25	#DIV/0!			0% (0.37)
41	14-3-3 protein theta OS=Mus musculus GN=Ywhaq PE=1 SV=1	1433T_MOUSE	28 kDa	0	0	2.4	4.16	#DIV/0!	14-3-3 θ and ζ bind to TERT.	Seimiya et al., EMBO J (2000) 11, 2652-2661.	0% (0.37)
42	Protein Wiz OS=Mus musculus GN=Wiz PE=1 SV=2	WIZ_MOUSE	184 kDa	0	0	2.4	0.79	#DIV/0!			95% (0.0063)
43	E3 ubiquitin-protein ligase NEDD4 OS=Mus musculus GN=Nedd4 PE=1 SV=3	NEDD4_MOUSE	103 kDa	0	0	2.3	2	#DIV/0!			0% (0.12)
44	WD repeat-containing protein 18 OS=Mus musculus GN=Wdr18 PE=2 SV=1	WDR18_MOUSE	47 kDa	0	0	2.24	2.26	#DIV/0!			0% (0.16)
45	Polynucleotide 5'-hydroxyl-kinase NOL9 OS=Mus musculus GN=Nol9 PE=2 SV=1	NOL9_MOUSE	81 kDa	0	0	2.2	3.81	#DIV/0!			0% (0.37)
46	WD repeat-containing protein 82 OS=Mus musculus GN=Wdr82 PE=1 SV=1	WDR82_MOUSE	35 kDa	0	0	2.15	1.86	#DIV/0!			0% (0.12)
47	Sorcin OS=Mus musculus GN=Sri PE=1 SV=1	SORCN_MOUSE	22 kDa	0	0	2.1	3.64	#DIV/0!			0% (0.37)
48	Rab GDP dissociation inhibitor beta OS=Mus musculus GN=Gdi2 PE=1 SV=1	GDIB_MOUSE	51 kDa	0	0	1.83	3.18	#DIV/0!			0% (0.37)
49	N-alpha-acetyltransferase 38, NatC auxiliary subunit OS=Mus musculus GN=Naa38 PE=2 SV=3	NAA38_MOUSE (+3	10 kDa	0	0	1.83	3.18	#DIV/0!			0% (0.37)
50	Ras-related C3 botulinum toxin substrate 2 OS=Mus musculus GN=Rac2 PE=2 SV=1	RAC2_MOUSE	21 kDa	0	0	1.8	3.12	#DIV/0!			0% (0.37)
51	Ras-related protein Rap-1A OS=Mus musculus GN=Rap1a PE=2 SV=1	RAP1A_MOUSE (+1	21 kDa	0	0	1.8	3.12	#DIV/0!			0% (0.37)
52	60S ribosomal protein L7-like 1 OS=Mus musculus GN=Rpl7l1 PE=2 SV=1	RL7L_MOUSE	29 kDa	0	0	1.8	1.67	#DIV/0!			0% (0.14)
53	Peptidyl-prolyl cis-trans isomerase-like 2 OS=Mus musculus GN=Ppil2 PE=2 SV=2	PPIL2_MOUSE	59 kDa	0	0	1.75	3.04	#DIV/0!			0% (0.37)
54	Proteasome subunit alpha type-1 OS=Mus musculus GN=Psma1 PE=1 SV=1	PSA1_MOUSE	30 kDa	0	0	1.7	1.65	#DIV/0!			0% (0.15)
55	Guanine nucleotide-binding protein G(i) subunit alpha-2 OS=Mus musculus GN=Gnai2 PE=1 SV=5	GNAI2_MOUSE	40 kDa	0	0	1.7	1.65	#DIV/0!			0% (0.15)
56	Osteoclast-stimulating factor 1 OS=Mus musculus GN=Ostf1 PE=1 SV=2	OSTF1_MOUSE	24 kDa	0	0	1.65	1.58	#DIV/0!			0% (0.14)
57	60S ribosomal protein L27a OS=Mus musculus GN=Rpl27a PE=2 SV=5	RL27A_MOUSE	17 kDa	0	0	1.65	1.58	#DIV/0!			0% (0.14)
58	Mitogen-activated protein kinase 14 OS=Mus musculus GN=Mapk14 PE=1 SV=3	MK14_MOUSE	41 kDa	0	0	1.63	1.44	#DIV/0!			0% (0.12)
59	Hornerin OS=Homo sapiens GN=HRNR PE=1 SV=2	HORN_HUMAN	282 kDa	0	0	1.5	2.6	#DIV/0!			0% (0.37)
60	Mitogen-activated protein kinase 1 OS=Mus musculus GN=Mapk1 PE=1 SV=3	MK01_MOUSE	41 kDa	0	0	1.5	2.6	#DIV/0!			0% (0.37)
61	Actin-related protein 2/3 complex subunit 4 OS=Mus musculus GN=Arpc4 PE=1 SV=3	ARPC4_MOUSE	20 kDa	0	0	1.5	2.6	#DIV/0!			0% (0.37)
62	Craniofacial development protein 1 OS=Mus musculus GN=Cfdp1 PE=1 SV=1	CFDP1_MOUSE	33 kDa	0	0	1.5	2.6	#DIV/0!			0% (0.37)
63	Tyrosine-protein kinase CSK OS=Mus musculus GN=Csk PE=1 SV=2	CSK_MOUSE	51 kDa	0	0	1.5	2.6	#DIV/0!			0% (0.37)
64	ADP-ribosylation factor 4 OS=Mus musculus GN=Arf4 PE=2 SV=2	ARF4_MOUSE	20 kDa	0	0	1.5	2.6	#DIV/0!			0% (0.37)
65	ATP-dependent RNA helicase DDX54 OS=Mus musculus GN=Ddx54 PE=1 SV=1	DDX54_MOUSE	98 kDa	0	0	1.4	2.43	#DIV/0!			0% (0.37)
66	WD repeat-containing protein 5 OS=Mus musculus GN=Wdr5 PE=1 SV=1	WDR5_MOUSE	37 kDa	0	0	1.4	2.43	#DIV/0!			0% (0.37)
67	Mediator of RNA polymerase II transcription subunit 12 OS=Mus musculus GN=Med12 PE=2 SV=1	MED12_MOUSE	245 kDa	0	0	1.4	2.43	#DIV/0!			0% (0.37)
68	KRR1 small subunit processome component homolog OS=Mus musculus GN=Krr1 PE=2 SV=1	KRR1_MOUSE	44 kDa	0	0	1.33	1.17	#DIV/0!			0% (0.12)
69	Myb/SANT-like DNA-binding domain-containing protein 4 OS=Mus musculus GN=Msantd4 PE=2 SV=1	MSD4_MOUSE	0 kDa	0	0	1.33	1.17	#DIV/0!			0% (0.12)
70	Moesin OS=Mus musculus GN=Msn PE=1 SV=3	MOES_MOUSE	68 kDa	0	0	1.3	1.14	#DIV/0!			0% (0.12)
71	Cell division cycle protein 27 homolog OS=Mus musculus GN=Cdc27 PE=2 SV=1	CDC27_MOUSE	92 kDa	0	0	1.3	1.14	#DIV/0!			0% (0.12)
72	Actin-binding protein anillin OS=Mus musculus GN=Anln PE=1 SV=2	ANLN_MOUSE	123 kDa	0	0	1.2	2.08	#DIV/0!			0% (0.37)
73	CST complex subunit CTC1 OS=Mus musculus GN=Ctc1 PE=1 SV=2	CTC1_MOUSE	134 kDa	0	0	1.2	2.08	#DIV/0!			0% (0.37)
74	Adseverin OS=Mus musculus GN=Scin PE=1 SV=3	ADSV_MOUSE	80 kDa	0	0	1.2	2.08	#DIV/0!			0% (0.37)
75	Cysteine and glycine-rich protein 1 OS=Mus musculus GN=Csrp1 PE=1 SV=3	CSRP1_MOUSE	21 kDa	0	0	1.2	2.08	#DIV/0!			0% (0.37)
76	40S ribosomal protein S21 OS=Mus musculus GN=Rps21 PE=2 SV=1	RS21_MOUSE	9 kDa	0	0	1.2	2.08	#DIV/0!			0% (0.37)
77	NudC domain-containing protein 2 OS=Mus musculus GN=Nudcd2 PE=1 SV=1	NUDC2_MOUSE	18 kDa	0	0	1.2	2.08	#DIV/0!			0% (0.37)
78	Fas apoptotic inhibitory molecule 1 OS=Mus musculus GN=Faim PE=1 SV=1	FAIM1_MOUSE	20 kDa	0	0	1.2	2.08	#DIV/0!			0% (0.37)
79	Eukaryotic translation initiation factor 4E OS=Mus musculus GN=Eif4e PE=1 SV=1	IF4E_MOUSE	25 kDa	0	0	1.2	2.08	#DIV/0!			0% (0.37)
80	Nucleoporin SEH1 OS=Mus musculus GN=Seh1l PE=2 SV=1	SEH1_MOUSE	40 kDa	0	0	1.1	1.91	#DIV/0!			0% (0.37)
81	Nuclear migration protein nudC OS=Mus musculus GN=Nudc PE=1 SV=1	NUDC_MOUSE	38 kDa	0	0	1.1	1.91	#DIV/0!			0% (0.37)
82	Ubiquitin-conjugating enzyme E2 N OS=Mus musculus GN=Ube2n PE=1 SV=1	UBE2N_MOUSE	17 kDa	0	0	1.1	1.91	#DIV/0!			0% (0.37)
83	Calcyclin-binding protein OS=Mus musculus GN=Cacybp PE=1 SV=1	CYBP_MOUSE	27 kDa	0	0	1.05	1.82	#DIV/0!			0% (0.37)
84	Protein quaking OS=Mus musculus GN=Qki PE=1 SV=1	QKI_MOUSE	38 kDa	0	0	1.05	1.82	#DIV/0!			0% (0.37)
85	Probable RNA-binding protein 19 OS=Mus musculus GN=Rbm19 PE=1 SV=1	RBM19_MOUSE	106 kDa	0	0	1.05	1.82	#DIV/0!			0% (0.37)
86	Serine/arginine-rich splicing factor 9 OS=Mus musculus GN=Srsf9 PE=1 SV=1	SRSF9_MOUSE	26 kDa	0	0	1.05	1.82	#DIV/0!			0% (0.37)
87	CLU_MOUSE	CLU_MOUSE	0 kDa	0	0	1.05	1.82	#DIV/0!			0% (0.37)
88	Histone acetyltransferase type B catalytic subunit OS=Mus musculus GN=Hat1 PE=2 SV=1	HAT1_MOUSE	49 kDa	0	0	1.05	1.82	#DIV/0!	HAT1 is required for telomeric silencing in S. pombe	Tong, Eukaryotic Cell (2005) 11, 1095-1103.	0% (0.37)
89	RNA-binding protein with multiple splicing 2 OS=Mus musculus GN=Rbpms2 PE=1 SV=1	RBPS2_MOUSE	22 kDa	0	0	1.05	1.82	#DIV/0!			0% (0.37)
90	Protein polybromo-1 OS=Mus musculus GN=Pbrm1 PE=1 SV=4	PB1_MOUSE	187 kDa	0	0	0.9	1.56	#DIV/0!			0% (0.37)
91	Tyrosine-protein phosphatase non-receptor type 2 OS=Mus musculus GN=Ptpn2 PE=2 SV=2	PTN2_MOUSE	47 kDa	0	0	0.9	1.56	#DIV/0!			0% (0.37)
92	Clathrin heavy chain 1 OS=Mus musculus GN=Cltc PE=1 SV=3	CLH_MOUSE (+1)	192 kDa	0	0	0.9	1.56	#DIV/0!			0% (0.37)
93	Homeobox protein cut-like 1 OS=Mus musculus GN=Cux1 PE=1 SV=3	CUX1_MOUSE	166 kDa	0	0	0.9	1.56	#DIV/0!			0% (0.37)
94	Clathrin interactor 1 OS=Mus musculus GN=Clint1 PE=1 SV=2	EPN4_MOUSE	69 kDa	0	0	0.9	1.56	#DIV/0!			0% (0.37)
95	Glyoxylate reductase/hydroxypyruvate reductase OS=Mus musculus GN=Grhpr PE=1 SV=1	GRHPR_MOUSE	35 kDa	0	0	0.9	1.56	#DIV/0!			0% (0.37)
96	Neurobeachin-like protein 2 OS=Mus musculus GN=Nbeal2 PE=2 SV=2	NBEL2_MOUSE	302 kDa	0	0	0.9	1.56	#DIV/0!			0% (0.37)
97	40S ribosomal protein S12 OS=Mus musculus GN=Rps12 PE=1 SV=2	RS12_MOUSE	15 kDa	0	0	0.9	1.56	#DIV/0!			0% (0.37)
98	Transaldolase OS=Mus musculus GN=Taldo1 PE=1 SV=2	TALDO_MOUSE	37 kDa	0	0	0.9	1.56	#DIV/0!			0% (0.37)
99	WD repeat-containing protein 1 OS=Mus musculus GN=Wdr1 PE=1 SV=3	WDR1_MOUSE	66 kDa	0	0	0.9	1.56	#DIV/0!			0% (0.37)
100	Nucleosome assembly protein 1-like 1 OS=Mus musculus GN=Nap1l1 PE=1 SV=2	NP1L1_MOUSE	45 kDa	0	0	0.73	1.27	#DIV/0!			0% (0.37)
101	Lysine-specific demethylase 3B OS=Mus musculus GN=Kdm3b PE=1 SV=2	KDM3B_MOUSE	171 kDa	0	0	0.73	1.27	#DIV/0!			0% (0.37)
102	Nuclear receptor corepressor 1 OS=Mus musculus GN=Ncor1 PE=1 SV=1	NCOR1_MOUSE	271 kDa	0	0	0.73	1.27	#DIV/0!			0% (0.37)
103	Growth factor receptor-bound protein 2 OS=Mus musculus GN=Grb2 PE=1 SV=1	GRB2_MOUSE	25 kDa	0	0	0.73	1.27	#DIV/0!			0% (0.37)
104	Eukaryotic peptide chain release factor GTP-binding subunit ERF3A OS=Mus musculus GN=Gspt1 PE=1 SV=1	ERF3A_MOUSE	56 kDa	0	0	0.73	1.27	#DIV/0!			0% (0.37)
105	Citron Rho-interacting kinase OS=Mus musculus GN=Cit PE=1 SV=3	CTRO_MOUSE	235 kDa	0	0	0.73	1.27	#DIV/0!			0% (0.37)
106	Asparagine synthetase [glutamine-hydrolyzing] OS=Mus musculus GN=Asns PE=2 SV=3	ASNS_MOUSE	64 kDa	0	0	0.73	1.27	#DIV/0!			0% (0.37)
107	Cytosolic acyl coenzyme A thioester hydrolase OS=Mus musculus GN=Acot7 PE=1 SV=2	BACH_MOUSE	43 kDa	0	0	0.73	1.27	#DIV/0!			0% (0.37)
108	DNA-dependent protein kinase catalytic subunit OS=Mus musculus GN=Prkdc PE=1 SV=3	PRKDC_MOUSE	471 kDa	0	0	0.73	1.27	#DIV/0!			0% (0.37)
109	Pre-mRNA-splicing factor RBM22 OS=Mus musculus GN=Rbm22 PE=2 SV=1	RBM22_MOUSE	47 kDa	0	0	0.73	1.27	#DIV/0!			0% (0.37)
110	Replication factor C subunit 5 OS=Mus musculus GN=Rfc5 PE=2 SV=1	RFC5_MOUSE	38 kDa	0	0	0.73	1.27	#DIV/0!			0% (0.37)
111	Splicing factor 3A subunit 3 OS=Mus musculus GN=Sf3a3 PE=2 SV=2	SF3A3_MOUSE	59 kDa	0	0	0.73	1.27	#DIV/0!			0% (0.37)
112	General transcription factor IIH subunit 2 OS=Mus musculus GN=Gtf2h2 PE=1 SV=1	TF2H2_MOUSE	45 kDa	0	0	0.73	1.27	#DIV/0!			0% (0.37)
113	U3 small nucleolar RNA-associated protein 6 homolog OS=Mus musculus GN=Utp6 PE=2 SV=1	UTP6_MOUSE	70 kDa	0	0	0.7	1.21	#DIV/0!			0% (0.37)
114	Nucleolar protein 6 OS=Mus musculus GN=Nol6 PE=2 SV=2	NOL6_MOUSE	129 kDa	0	0	0.7	1.21	#DIV/0!			0% (0.37)
115	Armadillo repeat-containing protein 6 OS=Mus musculus GN=Armc6 PE=2 SV=1	ARMC6_MOUSE	51 kDa	0	0	0.7	1.21	#DIV/0!	ARM6 binds to TRF2.	Giannone, PLoS One (2010) 5, e12407.	0% (0.37)
116	BEN domain-containing protein 3 OS=Mus musculus GN=Bend3 PE=2 SV=1	BEND3_MOUSE	94 kDa	0	0	0.7	1.21	#DIV/0!	BEND3 localizes to heterochromatin associated with HP1.	Sathyan, J. Cell Sci. (2011) 124, 3149-3163.	0% (0.37)
117	CDKN2A-interacting protein OS=Mus musculus GN=Cdkn2aip PE=2 SV=1	CARF_MOUSE	60 kDa	0	0	0.7	1.21	#DIV/0!			0% (0.37)
118	Cyclin-dependent kinase 7 OS=Mus musculus GN=Cdk7 PE=1 SV=2	CDK7_MOUSE	39 kDa	0	0	0.7	1.21	#DIV/0!	Loss of Cdk7 in the epidermis causes stem cell depletion and telo	Ganuza, EMBO J. (2012) 31, 2498-2510.	0% (0.37)
119	UPF0568 protein C14orf166 homolog OS=Mus musculus PE=2 SV=1	CN166_MOUSE	28 kDa	0	0	0.7	1.21	#DIV/0!			0% (0.37)
120	C-terminal-binding protein 1 OS=Mus musculus GN=Ctbp1 PE=1 SV=2	CTBP1_MOUSE	48 kDa	0	0	0.7	1.21	#DIV/0!	CtBP binds to FoxP2, which is associated with POT1.	Tanabe, Biochem. Biophys. Res. Commun. (2011) 410, 593-596.	0% (0.37)
121	High mobility group protein B3 OS=Mus musculus GN=Hmgb3 PE=2 SV=3	HMGB3_MOUSE	23 kDa	0	0	0.7	1.21	#DIV/0!			0% (0.37)
122	Importin-7 OS=Mus musculus GN=Ipo7 PE=1 SV=2	IPO7_MOUSE	119 kDa	0	0	0.7	1.21	#DIV/0!			0% (0.37)
123	Protein MAK16 homolog OS=Mus musculus GN=Mak16 PE=2 SV=1	MAK16_MOUSE	35 kDa	0	0	0.7	1.21	#DIV/0!			0% (0.37)
124	NF-kappa-B-repressing factor OS=Mus musculus GN=Nkrf PE=2 SV=3	NKRF_MOUSE	78 kDa	0	0	0.7	1.21	#DIV/0!			0% (0.37)
125	NmrA-like family domain-containing protein 1 OS=Mus musculus GN=Nmral1 PE=1 SV=1	NMRL1_MOUSE	34 kDa	0	0	0.7	1.21	#DIV/0!			0% (0.37)
126	Poly(A)-specific ribonuclease PARN OS=Mus musculus GN=Parn PE=1 SV=1	PARN_MOUSE	72 kDa	0	0	0.7	1.21	#DIV/0!			0% (0.37)
127	Core-binding factor subunit beta OS=Mus musculus GN=Cbfb PE=1 SV=1	PEBB_MOUSE	22 kDa	0	0	0.7	1.21	#DIV/0!			0% (0.37)
128	26S proteasome non-ATPase regulatory subunit 10 OS=Mus musculus GN=Psmd10 PE=1 SV=2	PSD10_MOUSE	25 kDa	0	0	0.7	1.21	#DIV/0!			0% (0.37)
129	SURP and G-patch domain-containing protein 1 OS=Mus musculus GN=Sugp1 PE=1 SV=1	SUGP1_MOUSE	73 kDa	0	0	0.7	1.21	#DIV/0!			0% (0.37)
130	Transferrin receptor protein 1 OS=Mus musculus GN=Tfrc PE=1 SV=1	TFR1_MOUSE	86 kDa	0	0	0.7	1.21	#DIV/0!			0% (0.37)
131	U3 small nucleolar RNA-associated protein 18 homolog OS=Mus musculus GN=Utp18 PE=1 SV=1	UTP18_MOUSE	61 kDa	0	0	0.7	1.21	#DIV/0!			0% (0.37)
132	Exportin-T OS=Mus musculus GN=Xpot PE=2 SV=3	XPOT_MOUSE	110 kDa	0	0	0.7	1.21	#DIV/0!	Exportin-1 binds to TERT.	Seimiya et al., EMBO J (2000) 11, 2652-2661.	0% (0.37)
133	Zinc fingers and homeoboxes protein 2 OS=Mus musculus GN=Zhx2 PE=1 SV=1	ZHX2_MOUSE	92 kDa	0	0	0.7	1.21	#DIV/0!			0% (0.37)
134	General transcription factor 3C polypeptide 4 OS=Mus musculus GN=Gtf3c4 PE=1 SV=2	TF3C4_MOUSE	92 kDa	0	0	0.7	1.21	#DIV/0!			0% (0.37)
135	U3 small nucleolar ribonucleoprotein protein IMP4 OS=Mus musculus GN=Imp4 PE=2 SV=1	IMP4_MOUSE	34 kDa	0	0	0.7	1.21	#DIV/0!	Budding yeast Imp4p binds to telomere *in vivo*.	Hsieh, Biochem. J. (2007) 408, 387-393.	0% (0.37)
136	Ubiquitin carboxyl-terminal hydrolase 4 OS=Mus musculus GN=Usp4 PE=1 SV=3	UBP4_MOUSE	108 kDa	0	0	0.7	1.21	#DIV/0!			0% (0.37)
137	Coronin-1A OS=Mus musculus GN=Coro1a PE=1 SV=5	COR1A_MOUSE	51 kDa	0	0	0.6	1.04	#DIV/0!			0% (0.37)
138	General transcription factor 3C polypeptide 1 OS=Mus musculus GN=Gtf3c1 PE=2 SV=2	TF3C1_MOUSE	237 kDa	0	0	0.6	1.04	#DIV/0!			0% (0.37)
139	Pumilio homolog 1 OS=Mus musculus GN=Pum1 PE=1 SV=2	PUM1_MOUSE (+1)	127 kDa	0	0	0.6	1.04	#DIV/0!			0% (0.37)
140	Small subunit processome component 20 homolog OS=Mus musculus GN=Utp20 PE=2 SV=2	UTP20_MOUSE	318 kDa	0	0	0.6	1.04	#DIV/0!			0% (0.37)
141	Pre-mRNA branch site protein p14 OS=Mus musculus GN=Sf3b14 PE=2 SV=1	PM14_MOUSE	15 kDa	0	0	0.6	1.04	#DIV/0!			0% (0.37)

142	U5 small nuclear ribonucleoprotein 40 kDa protein OS=Mus musculus GN=Snrnp40 PE=2 SV=1	SNR40_MOUSE	39 kDa	0	0	0.6	1.04	#DIV/0!			0% (0.37)
143	TFIIH basal transcription factor complex helicase XPB subunit OS=Mus musculus GN=Ercc3 PE=2 SV=1	ERCC3_MOUSE	89 kDa	0	0	0.6	1.04	#DIV/0!			0% (0.37)
144	DNA polymerase delta subunit 3 OS=Mus musculus GN=Pold3 PE=1 SV=2	DPOD3_MOUSE	51 kDa	0	0	0.6	1.04	#DIV/0!			0% (0.37)
145	Proteasome-associated protein ECM29 homolog OS=Mus musculus GN=Ecm29 PE=1 SV=3	ECM29_MOUSE	204 kDa	0	0	0.6	1.04	#DIV/0!			0% (0.37)
146	Eyes absent homolog 3 OS=Mus musculus GN=Eya3 PE=1 SV=1	EYA3_MOUSE	56 kDa	0	0	0.6	1.04	#DIV/0!			0% (0.37)
147	Eukaryotic translation initiation factor 6 OS=Mus musculus GN=Eif6 PE=1 SV=2	IF6_MOUSE	27 kDa	0	0	0.6	1.04	#DIV/0!			0% (0.37)
148	Peroxiredoxin-2 OS=Mus musculus GN=Prdx2 PE=1 SV=3	PRDX2_MOUSE	22 kDa	0	0	0.6	1.04	#DIV/0!			0% (0.37)
149	Ribose-phosphate pyrophosphokinase 1 OS=Mus musculus GN=Prps1 PE=1 SV=4	PRPS1_MOUSE	35 kDa	0	0	0.6	1.04	#DIV/0!			0% (0.37)
150	Septin-2 OS=Mus musculus GN=Sept2 PE=1 SV=2	SEPT2_MOUSE	42 kDa	0	0	0.6	1.04	#DIV/0!			0% (0.37)
151	Nucleoprotein TPR OS=Homo sapiens GN=TPR PE=1 SV=3	TPR_HUMAN	267 kDa	0	0	0.6	1.04	#DIV/0!			0% (0.37)
152	Ubiquitin carboxyl-terminal hydrolase 14 OS=Mus musculus GN=Usp14 PE=1 SV=3	UBP14_MOUSE	56 kDa	0	0	0.6	1.04	#DIV/0!			0% (0.37)
153	Uridine 5'-monophosphate synthase OS=Mus musculus GN=Umps PE=2 SV=3	UMPS_MOUSE	52 kDa	0	0	0.6	1.04	#DIV/0!			0% (0.37)
154	WD repeat-containing protein 61 OS=Mus musculus GN=Wdr61 PE=2 SV=1	WDR61_MOUSE	34 kDa	0	0	0.6	1.04	#DIV/0!			0% (0.37)
155	Zinc finger protein 354C OS=Mus musculus GN=Znf354c PE=2 SV=2	Z354C_MOUSE (+6)	64 kDa	0	0	0.6	1.04	#DIV/0!			0% (0.37)
156	Ribonuclease inhibitor OS=Mus musculus GN=Rnh1 PE=1 SV=1	RINI_MOUSE	50 kDa	1.32	2.29	20.68	3.76	15.67			95% (0.0016)
157	Protein phosphatase 1G OS=Mus musculus GN=Ppm1g PE=2 SV=3	PPM1G_MOUSE	59 kDa	1.04	1.81	9.18	6.69	8.8			0% (0.11)
158	Pre-mRNA-splicing factor SYF1 OS=Mus musculus GN=Xab2 PE=2 SV=1	SYF1_MOUSE	100 kDa	0.69	1.2	5.32	0.96	7.68			95% (0.0065)
159	UDP-N-acetylglucosamine--peptide N-acetylglucosaminyltransferase 110 kDa subunit OS=Mus musculus GN=Ogt	OGT1_MOUSE	117 kDa	0.63	1.09	4.67	0.65	7.42			95% (0.0052)
160	DnaJ homolog subfamily A member 2 OS=Mus musculus GN=Dnaja2 PE=1 SV=1	DNJA2_MOUSE	46 kDa	0.69	1.2	5.07	1.07	7.32			95% (0.0093)
161	DNA-binding protein Ikaros OS=Mus musculus GN=Ikzf1 PE=1 SV=2	IKZF1_MOUSE	57 kDa	3.67	1.59	25.82	6.88	7.04			95% (0.0056)
162	Ubiquitin carboxyl-terminal hydrolase 5 OS=Mus musculus GN=Usp5 PE=1 SV=1	UBP5_MOUSE	96 kDa	0.69	1.2	4.71	5.35	6.79			0% (0.27)
163	Aldose reductase OS=Mus musculus GN=Akr1b1 PE=1 SV=3	ALDR_MOUSE	36 kDa	1.04	1.81	6.37	1.89	6.11			95% (0.024)
164	Nucleus accumbens-associated protein 1 OS=Mus musculus GN=Nacc1 PE=2 SV=1	NACC1_MOUSE	57 kDa	3.06	3.79	18.3	11.03	5.97			0% (0.086)
165	S-formylglutathione hydrolase OS=Mus musculus GN=Esd PE=2 SV=1	ESTD_MOUSE	31 kDa	0.69	1.2	4.08	1.25	5.89			95% (0.027)
166	Proteasome activator complex subunit 3 OS=Mus musculus GN=Psme3 PE=1 SV=1	PSME3_MOUSE	30 kDa	0.69	1.2	3.95	1.26	5.7			95% (0.032)
167	DNA polymerase epsilon catalytic subunit A OS=Mus musculus GN=Pole PE=2 SV=3	DPOE1_MOUSE	262 kDa	0.69	1.2	3.89	4.95	5.61	Budding yeast DPOE is required for maintenance of replicative se	Deshpande, Mol. Cell Biol. (2011) 31, 1637-1645.	0% (0.34)
168	WD repeat and HMG-box DNA-binding protein 1 OS=Mus musculus GN=Wdhd1 PE=1 SV=2	WDHD1_MOUSE	124 kDa	5.08	5.85	26.13	19.32	5.14			0% (0.15)
169	tRNA-splicing ligase RtcB homolog OS=Mus musculus GN=D10Wsu52e PE=2 SV=1	RTCB_MOUSE	55 kDa	0.63	1.09	3.2	3.16	5.08			0% (0.25)
170	Low molecular weight phosphotyrosine protein phosphatase OS=Mus musculus GN=Acp1 PE=1 SV=3	PPAC_MOUSE	18 kDa	0.69	1.2	3.29	1.16	4.74			0% (0.055)
171	Muscleblind-like protein 1 OS=Mus musculus GN=Mbnl1 PE=1 SV=1	MBNL1_MOUSE	37 kDa	1.65	2.85	7.79	0.88	4.73			95% (0.024)
172	Cell division cycle protein 16 homolog OS=Mus musculus GN=Cdc16 PE=2 SV=1	CDC16_MOUSE	71 kDa	0.63	1.09	2.97	2.57	4.71			0% (0.22)
173	Talin-1 OS=Mus musculus GN=Tln1 PE=1 SV=2	TLN1_MOUSE	270 kDa	8.8	4.79	37.77	9.97	4.29			95% (0.011)
174	Hemoglobin subunit beta-2 OS=Mus musculus GN=Hbb-b2 PE=1 SV=2	HBB2_MOUSE	16 kDa	0.63	1.09	2.6	2.33	4.13			0% (0.26)
175	Zinc finger MYM-type protein 4 OS=Mus musculus GN=Zmym4 PE=1 SV=1	ZMYM4_MOUSE	172 kDa	0.99	1.71	4	4.22	4.04			0% (0.32)
176	CUGBP Elav-like family member 2 OS=Mus musculus GN=Celf2 PE=1 SV=1	CELF2_MOUSE	54 kDa	2.68	1.29	10.54	3.63	3.93			95% (0.024)
177	Thioredoxin-like protein 1 OS=Mus musculus GN=Txnl1 PE=1 SV=3	TXNL1_MOUSE	32 kDa	0.99	1.71	3.8	4.08	3.84			0% (0.33)
178	Ribonucleoprotein PTB-binding 1 OS=Mus musculus GN=Raver1 PE=1 SV=2	RAVR1_MOUSE	79 kDa	5.71	5.74	21.5	7.97	3.76			95% (0.050)
179	Actin-like protein 6A OS=Mus musculus GN=Actl6a PE=1 SV=2	ACL6A_MOUSE	47 kDa	0.63	1.09	2.37	2.22	3.76			0% (0.29)
180	Galactokinase OS=Mus musculus GN=Galk1 PE=2 SV=1	GALK1_MOUSE	42 kDa	2.99	1.1	11.1	2.45	3.71			95% (0.0064)
181	E3 ubiquitin-protein ligase BRE1B OS=Mus musculus GN=Rnf40 PE=2 SV=2	BRE1B_MOUSE	114 kDa	2.08	3.61	7.73	3.61	3.71			0% (0.13)
182	ADP-ribosylation factor 1 OS=Mus musculus GN=Arf1 PE=1 SV=2	ARF1_MOUSE (+1)	21 kDa	1.26	2.18	4.67	4.1	3.71			0% (0.27)
183	Acidic leucine-rich nuclear phosphoprotein 32 family member B OS=Mus musculus GN=Anp32b PE=1 SV=1	AN32B_MOUSE	31 kDa	3.13	5.42	10.92	3.53	3.49			0% (0.10)
184	Galectin-9 OS=Mus musculus GN=Lgals9 PE=1 SV=1	LEG9_MOUSE	40 kDa	2.59	2.48	9.02	3.26	3.48			0% (0.053)
185	Transcriptional repressor p66 alpha OS=Mus musculus GN=Gatad2a PE=1 SV=2	P66A_MOUSE	67 kDa	2.62	2.4	9.09	3.49	3.47			0% (0.057)
186	E3 ubiquitin-protein ligase UBR5 OS=Mus musculus GN=Ubr5 PE=1 SV=2	UBR5_MOUSE	308 kDa	0.94	1.63	3.27	2.88	3.47			0% (0.29)
187	Malate dehydrogenase, cytoplasmic OS=Mus musculus GN=Mdh1 PE=1 SV=3	MDHC_MOUSE	37 kDa	0.99	1.71	3.4	4.21	3.44			0% (0.41)
188	Clathrin heavy chain 1 OS=Mus musculus GN=Cltc PE=1 SV=3	CLH1_MOUSE	0 kDa	2.58	2.24	8.73	7.9	3.39			0% (0.26)
189	Caspase-3 OS=Mus musculus GN=Casp3 PE=1 SV=1	CASP3_MOUSE	31 kDa	0.69	1.2	2.33	0.32	3.37			0% (0.085)
190	SWI/SNF complex subunit SMARCC1 OS=Mus musculus GN=Smarcc1 PE=1 SV=2	SMRC1_MOUSE	123 kDa	3	2.69	9.99	1.53	3.33	The SWI/SNF complex is required for telomeric silencing in buddir	Dror, Mol. Cell Biol. (2004) 24, 8227-8235.	95% (0.017)
191	Exportin-5 OS=Mus musculus GN=Xpo5 PE=2 SV=1	XPO5_MOUSE	137 kDa	4.58	1.85	14.98	3.66	3.27	Exportin-1 binds to TERT.	Seimiya et al., EMBO J. (2000) 11, 2652-2661.	95% (0.012)
192	Cleavage stimulation factor subunit 3 OS=Mus musculus GN=Cstf3 PE=1 SV=1	CSTF3_MOUSE	83 kDa	1.29	1.12	4.15	2.13	3.22			0% (0.11)
193	Chromodomain-helicase-DNA-binding protein 8 OS=Mus musculus GN=Chd8 PE=1 SV=1	CHD8_MOUSE	291 kDa	1.04	1.81	3.34	2.93	3.2			0% (0.31)
194	Aldo-keto reductase family 1 member C13 OS=Mus musculus GN=Akr1c13 PE=1 SV=2	AK1CD_MOUSE	37 kDa	8.17	2.76	25.19	13.68	3.08			0% (0.10)
195	Anaphase-promoting complex subunit 4 OS=Mus musculus GN=Anapc4 PE=2 SV=1	APC4_MOUSE	92 kDa	0.63	1.09	1.93	1.81	3.07			0% (0.35)
196	Myelin expression factor 2 OS=Mus musculus GN=Myef2 PE=1 SV=1	MYEF2_MOUSE	63 kDa	1.39	2.41	4.25	4.8	3.06			0% (0.41)
197	Lysine-specific demethylase 5C OS=Mus musculus GN=Kdm5c PE=2 SV=4	KDM5C_MOUSE	175 kDa	0.69	1.2	2.1	3.64	3.03	Yeast LSD1 homologue localizes in heterochromatin including subtelomeric regions.<br>Yeast LSD1 mutations cause spreading of telomeric heterochromatin.	Gordon, MCB (2007), 4058-4069.	0% (0.56)
198	MKI67 FHA domain-interacting nucleolar phosphoprotein OS=Mus musculus GN=Mki67ip PE=2 SV=1	MK67I_MOUSE	36 kDa	0.99	1.71	2.95	2.69	2.98			0% (0.35)
199	Nuclear autoantigenic sperm protein OS=Mus musculus GN=Nasp PE=1 SV=2	NASP_MOUSE	84 kDa	1.26	2.18	3.7	3.37	2.94			0% (0.35)
200	E3 ubiquitin-protein ligase BRE1A OS=Mus musculus GN=Rnf20 PE=1 SV=2	BRE1A_MOUSE	114 kDa	2.05	2.09	5.97	1.43	2.91			0% (0.055)
201	Nuclear pore complex protein Nup107 OS=Mus musculus GN=Nup107 PE=2 SV=1	NU107_MOUSE	107 kDa	0.63	1.09	1.83	3.18	2.91			0% (0.57)
202	Heterogeneous nuclear ribonucleoprotein H2 OS=Mus musculus GN=Hnrnph2 PE=1 SV=1	HNRH2_MOUSE	49 kDa	14.59	25.28	42.11	5.26	2.89			0% (0.14)
203	14-3-3 protein beta/alpha OS=Mus musculus GN=Ywhab PE=1 SV=3	1433B_MOUSE	28 kDa	2.83	4.91	8.04	7.69	2.84	14-3-3θ and ζ bind to TERT.	Seimiya et al., EMBO J (2000) 11, 2652-2661.	0% (0.38)
204	Targeting protein for Xklp2 OS=Mus musculus GN=Tpx2 PE=1 SV=1	TPX2_MOUSE	86 kDa	2.43	4.21	6.89	7.69	2.83			0% (0.43)
205	Nuclear pore complex protein Nup133 OS=Mus musculus GN=Nup133 PE=1 SV=2	NU133_MOUSE	129 kDa	1.93	1.68	5.37	2.92	2.78	Eroded telomeres relocalize to the nuclear pore complex (NPC).<br>Tethering of telomeres to the NPC in senescing cells.	Khadaroo, Nat. Cell Biol. (2009) 11, 980-987.	0% (0.15)
206	Glutaredoxin-3 OS=Mus musculus GN=Glrx3 PE=1 SV=1	GLRX3_MOUSE	38 kDa	3.03	3.13	8.39	3.04	2.77	Glutaredoxin, a substrate of Bud32p kinase, involved in telomere h	Peggion, FEBS J. (2008) 275, 5919-5933	0% (0.10)
207	Parafibromin OS=Mus musculus GN=Cdc73 PE=2 SV=1	CDC73_MOUSE	61 kDa	0.69	1.2	1.9	1.81	2.74			0% (0.39)
208	Acidic leucine-rich nuclear phosphoprotein 32 family member E OS=Mus musculus GN=Anp32e PE=1 SV=2	AN32E_MOUSE	30 kDa	3.9	1.8	10.44	5.7	2.67			0% (0.13)
209	CUGBP Elav-like family member 1 OS=Mus musculus GN=Celf1 PE=1 SV=2	CELF1_MOUSE	52 kDa	1.98	3.42	5.27	4.57	2.67			0% (0.37)
210	Histone-arginine methyltransferase CARM1 OS=Mus musculus GN=Carm1 PE=1 SV=2	CARM1_MOUSE	66 kDa	2.61	1.01	6.8	2.87	2.6	Yeast LSD1 homologue localizes in heterochromatin.	Gordon, MCB (2007), 4058-4069.	0% (0.076)
211	Farnesyl pyrophosphate synthase OS=Mus musculus GN=Fdps PE=2 SV=1	FPPS_MOUSE	41 kDa	1.26	2.18	3.2	3.16	2.54			0% (0.43)
212	Exportin-1 OS=Mus musculus GN=Xpo1 PE=1 SV=1	XPO1_MOUSE	123 kDa	4.52	3.48	11.46	5.7	2.54	Exportin-1 binds to TERT.	Seimiya et al., EMBO J. (2000) 11, 2652-2661.	0% (0.15)
213	Nuclear valosin-containing protein-like OS=Mus musculus GN=Nvl PE=1 SV=1	NVL_MOUSE	94 kDa	0.69	1.2	1.75	3.04	2.53			0% (0.60)
214	DnaJ homolog subfamily C member 9 OS=Mus musculus GN=Dnajc9 PE=2 SV=2	DNJC9_MOUSE	30 kDa	1.39	2.41	3.51	3.21	2.52			0% (0.41)
215	Profilin-1 OS=Mus musculus GN=Pfn1 PE=1 SV=2	PROF1_MOUSE	15 kDa	10.68	6.42	26.86	15.6	2.51			0% (0.17)
216	NHP2-like protein 1 OS=Mus musculus GN=Nhp2l1 PE=2 SV=4	NH2L1_MOUSE	14 kDa	0.69	1.2	1.7	1.65	2.45			0% (0.44)
217	Cleavage and polyadenylation specificity factor subunit 2 OS=Mus musculus GN=Cpsf2 PE=1 SV=1	CPSF2_MOUSE	88 kDa	2.96	2.58	7.07	0.71	2.39			0% (0.056)
218	Cyclin-dependent kinase 6 OS=Mus musculus GN=Cdk6 PE=1 SV=2	CDK6_MOUSE	37 kDa	4.81	4.36	11.44	10.06	2.38			0% (0.35)
219	Lysine-specific histone demethylase 1A OS=Mus musculus GN=Kdm1a PE=1 SV=2	KDM1A_MOUSE	93 kDa	3.28	2.94	7.79	0.88	2.38	Fission yeast LSD1 homologue localizes in heterochromatin.	Gordon, MCB (2007), 4058-4069.	0% (0.063)
220	Peptidyl-prolyl cis-trans isomerase FKBP4 OS=Mus musculus GN=Fkbp4 PE=1 SV=5	FKBP4_MOUSE	52 kDa	1.92	1.89	4.54	4.14	2.36			0% (0.38)
221	Leukotriene A-4 hydrolase OS=Mus musculus GN=Lta4h PE=1 SV=4	LKHA4_MOUSE	69 kDa	4.85	3.3	11.32	5.25	2.34			0% (0.14)
222	Vigilin OS=Mus musculus GN=Hdlbp PE=1 SV=1	VIGLN_MOUSE	142 kDa	2.58	2.24	6.01	1.59	2.33			0% (0.096)
223	Homeobox protein Rhox5 OS=Mus musculus GN=Rhox5 PE=2 SV=1	RHOX5_MOUSE	23 kDa	4.2	3.69	9.45	6.1	2.25			0% (0.27)
224	Transcription elongation factor A protein 1 OS=Mus musculus GN=Tcea1 PE=1 SV=2	TCEA1_MOUSE	34 kDa	2.69	2.5	6.04	3.5	2.24			0% (0.25)
225	Ubiquitin-conjugating enzyme E2 variant 1 OS=Mus musculus GN=Ube2v1 PE=1 SV=1	UB2V1_MOUSE (+1	16 kDa	1.89	3.27	4.24	3.77	2.24			0% (0.46)
226	14-3-3 protein epsilon OS=Mus musculus GN=Ywhae PE=1 SV=1	1433E_MOUSE	29 kDa	4.86	2.74	10.85	6.84	2.23	14-3-3θ and ζ bind to TERT.	Seimiya et al., EMBO J (2000) 11, 2652-2661.	0% (0.23)
227	C-1-tetrahydrofolate synthase, cytoplasmic OS=Mus musculus GN=Mthfd1 PE=1 SV=4	C1TC_MOUSE	101 kDa	3.59	1.36	7.82	3.68	2.18			0% (0.13)
228	Activated RNA polymerase II transcriptional coactivator p15 OS=Mus musculus GN=Sub1 PE=1 SV=3	TCP4_MOUSE	14 kDa	1.98	3.42	4.3	3.73	2.18			0% (0.47)
229	Stress-induced-phosphoprotein 1 OS=Mus musculus GN=Stip1 PE=1 SV=1	STIP1_MOUSE	63 kDa	3.22	2.79	7.01	0.97	2.18			0% (0.091)
230	40S ribosomal protein S27-like OS=Mus musculus GN=Rps27l PE=2 SV=3	RS27L_MOUSE	9 kDa	0.69	1.2	1.5	2.6	2.17			0% (0.65)
231	Nuclear cap-binding protein subunit 1 OS=Mus musculus GN=Ncbp1 PE=1 SV=2	NCBP1_MOUSE	92 kDa	1.57	2.73	3.34	2.93	2.12			0% (0.49)
232	Chromosome-associated kinesin KIF4 OS=Mus musculus GN=Kif4 PE=2 SV=3	KIF4_MOUSE	140 kDa	11.62	2.07	24.28	18.15	2.09			0% (0.30)
233	Symplekin OS=Mus musculus GN=Sympk PE=1 SV=1	SYMPK_MOUSE	142 kDa	5.17	3.22	10.79	5.13	2.09			0% (0.18)
234	Ribosome biogenesis protein WDR12 OS=Mus musculus GN=Wdr12 PE=2 SV=1	WDR12_MOUSE	47 kDa	8.28	1.64	17.18	0.39	2.07			95% (0.00079)
235	Glutamate--cysteine ligase regulatory subunit OS=Mus musculus GN=Gclm PE=2 SV=1	GSH0_MOUSE	31 kDa	2.25	1.99	4.57	2.56	2.03	Thiol redox state may modulate telomerase activity	Brown, Free Radic. Biol. Med (2007) 42, 228-235.	0% (0.28)
236	Cleavage and polyadenylation specificity factor subunit 7 OS=Mus musculus GN=Cpsf7 PE=1 SV=2	CPSF7_MOUSE	52 kDa	0.69	1.2	1.4	2.43	2.02			0% (0.67)
237	Ribosomal RNA-processing protein 7 homolog A OS=Mus musculus GN=Rrp7a PE=2 SV=1	RRP7A_MOUSE	32 kDa	0.66	1.14	1.33	1.17	2.02			0% (0.51)
238	U1 small nuclear ribonucleoprotein A OS=Mus musculus GN=Snrpa PE=2 SV=3	SNRPA_MOUSE	32 kDa	2.89	2.53	5.84	3.92	2.02			0% (0.34)
239	Peroxiredoxin-1 OS=Mus musculus GN=Prdx1 PE=1 SV=1	PRDX1_MOUSE	22 kDa	5.91	3.53	11.82	4.93	2	Budding yeast nuclear peroxiredoxin disrupts telomeric silencing.	SInger, Genetics (1998) 150, 613-632.	0% (0.17)
240	14-3-3 protein zeta/delta OS=Mus musculus GN=Ywhaz PE=1 SV=1	1433Z_MOUSE	28 kDa	4.89	1.74	9.67	9.08	1.98	14-3-3θ and ζ bind to TERT.	Seimiya et al., EMBO J (2000) 11, 2652-2661.	0% (0.42)
241	Periodic tryptophan protein 2 homolog OS=Mus musculus GN=Pwp2 PE=1 SV=1	PWP2_MOUSE	103 kDa	1.98	0.1	3.8	2.3	1.92			0% (0.24)
242	Ribosomal biogenesis protein LAS1L OS=Mus musculus GN=Las1l PE=2 SV=1	LAS1L_MOUSE	89 kDa	1.39	2.41	2.65	2.63	1.91			0% (0.57)
243	Transformation/transcription domain-associated protein OS=Mus musculus GN=Trrap PE=1 SV=2	TRRAP_MOUSE	292 kDa	0.63	1.09	1.2	2.08	1.9			0% (0.69)
244	Polypyrimidine tract-binding protein 3 OS=Mus musculus GN=Ptbp3 PE=2 SV=1	PTBP3_MOUSE	57 kDa	7.88	9.02	15	9.79	1.9			0% (0.41)
245	U4/U6 small nuclear ribonucleoprotein Prp4 OS=Mus musculus GN=Prpf4 PE=2 SV=1	PRP4_MOUSE	58 kDa	0.69	1.2	1.3	1.14	1.88			0% (0.56)
246	E3 ubiquitin-protein ligase TRIM33 OS=Mus musculus GN=Trim33 PE=2 SV=2	TRI33_MOUSE	124 kDa	3.07	3.13	5.76	7.09	1.87			0% (0.58)
247	U4/U6 small nuclear ribonucleoprotein Prp3 OS=Mus musculus GN=Prpf3 PE=1 SV=1	PRPF3_MOUSE	77 kDa	1.93	1.68	3.59	1.65	1.86			0% (0.29)
248	RuvB-like 2 OS=Mus musculus GN=Ruvbl2 PE=2 SV=3	RUVB2_MOUSE	51 kDa	12.36	7.39	22.89	6.52	1.85			0% (0.14)
249	Phosphoglycerate kinase 1 OS=Mus musculus GN=Pgk1 PE=1 SV=4	PGK1_MOUSE	45 kDa	14.17	0.83	26.18	6.35	1.85			95% (0.032)
250	DNA polymerase alpha catalytic subunit OS=Mus musculus GN=Pola1 PE=1 SV=2	DPOLA_MOUSE	167 kDa	1.57	2.73	2.91	2.52	1.85	DPOLA binds to telomere protein Cdc13p in budding yeast.<br>A ts-mutant mouse cell lines show telomere defects.	Qi, Genes Dev. (2000) 14, 1777-1788.<br>Hsu, Nucleic Acids Res. (2004) 32, 511-521.<br>Nakamura, EMBO J. (2005) 25, 11073-11088.	0% (0.57)
251	HEAT repeat-containing protein 3 OS=Mus musculus GN=Heatr3 PE=2 SV=1	HEAT3_MOUSE	74 kDa	0.66	1.14	1.2	2.08	1.82			0% (0.71)
252	Transcription intermediary factor 1-beta OS=Mus musculus GN=Trim28 PE=1 SV=3	TIF1B_MOUSE	89 kDa	51.19	24.27	92.02	29.51	1.8			0% (0.14)
253	General transcription factor II-I OS=Mus musculus GN=Gtf2i PE=1 SV=3	GTF2I_MOUSE	112 kDa	18.75	10	33.42	8.42	1.78			0% (0.12)
254	Notchless protein homolog 1 OS=Mus musculus GN=Nle1 PE=2 SV=4	NLE1_MOUSE	53 kDa	2.59	2.48	4.6	4	1.78			0% (0.50)
255	Chromodomain-helicase-DNA-binding protein 4 OS=Mus musculus GN=Chd4 PE=1 SV=1	CHD4_MOUSE	218 kDa	25.3	4.26	44.69	11.68	1.77			0% (0.054)
256	RNA-binding protein 5 OS=Mus musculus GN=Rbm5 PE=1 SV=1	RBM5_MOUSE	92 kDa	1.39	2.41	2.45	4.25	1.76			0% (0.73)
257	Polyadenylate-binding protein 2 OS=Mus musculus GN=Pabpn1 PE=2 SV=3	PABP2_MOUSE	32 kDa	3.47	6.02	6.11	7.67	1.76			0% (0.66)
258	40S ribosomal protein S16 OS=Mus musculus GN=Rps16 PE=2 SV=4	RS16_MOUSE	16 kDa	5.15	4.47	9.04	4.23	1.75			0% (0.34)
259	Exportin-2 OS=Mus musculus GN=Cse1l PE=2 SV=1	XPO2_MOUSE	110 kDa	7.26	2.01	12.72	2.83	1.75			0% (0.052)
260	CCAAT/enhancer-binding protein zeta OS=Mus musculus GN=Cebpz PE=2 SV=2	CEBPZ_MOUSE	120 kDa	4.76	4.29	8.29	6.55	1.74			0% (0.48)
261	PDZ and LIM domain protein 5 OS=Mus musculus GN=Pdlim5 PE=1 SV=4	PDLI5_MOUSE	63 kDa	0.94	1.63	1.63	1.44	1.73			0% (0.61)
262	Cyclin-dependent kinase 1 OS=Mus musculus GN=Cdk1 PE=1 SV=3	CDK1_MOUSE	34 kDa	7.44	4.8	12.82	6.95	1.72	CDK1 phosphorylates TRF1.	McKerlie, Nat. Commun. (2011) DOI:10.1038/ncomms1372, PICh table	0% (0.33)
263	Fermitin family homolog 3 OS=Mus musculus GN=Fermt3 PE=1 SV=1	URP2_MOUSE	76 kDa	5.47	4.75	9.39	3.63	1.72			0% (0.32)
264	ADP/ATP translocase 2 OS=Mus musculus GN=Slc25a5 PE=1 SV=3	ADT2_MOUSE	33 kDa	0.94	1.63	1.6	1.42	1.7			0% (0.63)
265	Heterogeneous nuclear ribonucleoprotein M OS=Mus musculus GN=Hnrnpm PE=1 SV=3	HNRPM_MOUSE	78 kDa	136.22	16.7	230.45	35.26	1.69			95% (0.014)
266	Deoxynucleotidyltransferase terminal-interacting protein 2 OS=Mus musculus GN=Dnttip2 PE=1 SV=1	TDIF2_MOUSE	84 kDa	1.04	1.81	1.75	3.04	1.68			0% (0.75)
267	SPB1_MOUSE	SPB1_MOUSE	0 kDa	1.04	1.81	1.75	3.04	1.68			0% (0.75)
268	Metastasis-associated protein MTA2 OS=Mus musculus GN=Mta2 PE=1 SV=1	MTA2_MOUSE	75 kDa	10.27	3.92	17.11	3.92	1.67			0% (0.099)
269	Ribosomal RNA small subunit methyltransferase NEP1 OS=Mus musculus GN=Emg1 PE=1 SV=1	NEP1_MOUSE	27 kDa	3.6	4.69	5.95	2.54	1.66			0% (0.49)
270	Interferon regulatory factor 2-binding protein 2 OS=Mus musculus GN=Irf2bp2 PE=1 SV=1	I2BP2_MOUSE	0 kDa	2.62	2.4	4.32	1.13	1.65			0% (0.33)
271	Poly [ADP-ribose] polymerase 1 OS=Mus musculus GN=Parp1 PE=1 SV=3	PARP1_MOUSE	113 kDa	14.02	5.96	23.02	11.46	1.64	PARP1 is a telomere-associated protein.	Gomez. Mol. Cell Biol. (2006) 17, 1686-1696.	0% (0.29)
272	U2 small nuclear ribonucleoprotein A' OS=Mus musculus GN=Snrpa1 PE=1 SV=2	RU2A_MOUSE	28 kDa	7.82	4.12	12.81	7.91	1.64			0% (0.39)
273	Protein PML OS=Mus musculus GN=Pml PE=1 SV=3	PML_MOUSE	98 kDa	12.92	2.29	20.85	5.06	1.61	PML is a telomere-associated protein.	Yeager, Cancer Res. (1999) 59, 4175-4179.	0% (0.069)
274	A-kinase anchor protein 8 OS=Mus musculus GN=Akap8 PE=1 SV=1	AKAP8_MOUSE	76 kDa	4.01	1.84	6.44	1.71	1.6			0% (0.17)
275	SNW domain-containing protein 1 OS=Mus musculus GN=Snw1 PE=1 SV=3	SNW1_MOUSE	61 kDa	3.04	2.66	4.87	2.56	1.6			0% (0.44)
276	Actin-related protein 2/3 complex subunit 1B OS=Mus musculus GN=Arpc1b PE=1 SV=4	ARC1B_MOUSE	41 kDa	1.29	1.12	2.07	2.21	1.6			0% (0.62)
277	Nuclear pore complex protein Nup160 OS=Mus musculus GN=Nup160 PE=1 SV=2	NU160_MOUSE	158 kDa	2.55	2.88	4.07	3.87	1.6			0% (0.61)
278	ATP-dependent RNA helicase DDX42 OS=Mus musculus GN=Ddx42 PE=1 SV=3	DDX42_MOUSE	102 kDa	4.01	3.7	6.36	3.08	1.59			0% (0.45)
279	TAR DNA-binding protein 43 OS=Mus musculus GN=Tardbp PE=1 SV=1	TADBP_MOUSE	45 kDa	28.84	3.95	45.73	11.86	1.59			0% (0.079)
280	Paraspeckle component 1 OS=Mus musculus GN=Pspc1 PE=1 SV=1	PSPC1_MOUSE	59 kDa	6.02	2.93	9.53	3.6	1.58			0% (0.26)
281	Ubiquitin-like modifier-activating enzyme 1 OS=Mus musculus GN=Uba1 PE=1 SV=1	UBA1_MOUSE	118 kDa	5.98	10.36	9.44	8.43	1.58			0% (0.68)
282	Exosome complex exonuclease RRP44 OS=Mus musculus GN=Dis3 PE=2 SV=4	RRP44_MOUSE	109 kDa	8.4	5.37	13.17	3.18	1.57			0% (0.26)
283	Cleavage and polyadenylation specificity factor subunit 3 OS=Mus musculus GN=Cpsf3 PE=1 SV=2	CPSF3_MOUSE	78 kDa	4.96	0.25	7.76	1.51	1.56			95% (0.034)
284	Mitotic checkpoint protein BUB3 OS=Mus musculus GN=Bub3 PE=2 SV=2	BUB3_MOUSE	37 kDa	9.78	5.02	15.24	7.8	1.56			0% (0.37)
285	Eukaryotic translation initiation factor 5A-1 OS=Mus musculus GN=Eif5a PE=1 SV=2	IF5A1_MOUSE	17 kDa	9.08	5	13.96	5.76	1.54			0% (0.33)
286	Chromobox protein homolog 3 OS=Homo sapiens GN=CBX3 PE=1 SV=4	CBX3_HUMAN (+1)	21 kDa	5.86	4.24	8.97	4.14	1.53			0% (0.41)
287	Coiled-coil domain-containing protein 86 OS=Mus musculus GN=Ccdc86 PE=1 SV=2	CCD86_MOUSE	46 kDa	0.69	1.2	1.05	1.82	1.51			0% (0.79)
288	DNA polymerase delta catalytic subunit OS=Mus musculus GN=Pold1 PE=1 SV=2	DPOD1_MOUSE	124 kDa	4.07	4.81	6.14	5.66	1.51			0% (0.65)

289	Glyceraldehyde-3-phosphate dehydrogenase OS=Mus musculus GN=Gapdh PE=1 SV=2	G3P_MOUSE	36 kDa	56.47	19.13	85.01	44.55	1.51			0% (0.37)
290	Putative RNA-binding protein 3 OS=Mus musculus GN=Rbm3 PE=1 SV=1	RBM3_MOUSE	17 kDa	2.91	2.58	4.37	3.95	1.5			0% (0.62)
291	Splicing factor 1 OS=Mus musculus GN=Sf1 PE=1 SV=6	SF01_MOUSE	70 kDa	6.62	2.11	9.91	2.71	1.5			0% (0.17)
292	Serine/threonine-protein phosphatase PP1-alpha catalytic subunit OS=Mus musculus GN=Ppp1ca PE=1 SV=1	PP1A_MOUSE	38 kDa	8.53	1.98	12.74	3.78	1.49			0% (0.16)
293	Matrin-3 OS=Mus musculus GN=Matr3 PE=1 SV=1	MATR3_MOUSE	95 kDa	52.24	24.49	77.9	33.23	1.49			0% (0.34)
294	Heterogeneous nuclear ribonucleoprotein A0 OS=Mus musculus GN=Hnrnpa0 PE=1 SV=1	ROA0_MOUSE	31 kDa	18.51	2.94	27.51	11.44	1.49			0% (0.26)
295	RuvB-like 1 OS=Mus musculus GN=Ruvbl1 PE=1 SV=1	RUVB1_MOUSE	50 kDa	19.38	12.93	28.75	13.56	1.48			0% (0.44)
296	6-phosphogluconate dehydrogenase, decarboxylating OS=Mus musculus GN=Pgd PE=2 SV=3	6PGD_MOUSE	53 kDa	8.27	1.25	12.19	2.99	1.47			0% (0.10)
297	ATP-dependent RNA helicase DDX3X OS=Mus musculus GN=Ddx3x PE=1 SV=3	DDX3X_MOUSE	73 kDa	14.67	5.4	21.57	11.47	1.47			0% (0.40)
298	Nucleolar GTP-binding protein 1 OS=Mus musculus GN=Gtpbp4 PE=2 SV=3	NOG1_MOUSE	74 kDa	9.91	13.29	14.55	13.01	1.47			0% (0.69)
299	Protein strawberry notch homolog 1 OS=Mus musculus GN=Sbno1 PE=1 SV=2	SBNO1_MOUSE	154 kDa	4.97	1.09	7.25	2.69	1.46			0% (0.25)
300	Alpha-enolase OS=Mus musculus GN=Eno1 PE=1 SV=3	ENOA_MOUSE	47 kDa	27.47	8.34	40.04	19.57	1.46			0% (0.36)
301	Eukaryotic translation initiation factor 2 subunit 1 OS=Mus musculus GN=Eif2s1 PE=1 SV=3	IF2A_MOUSE	36 kDa	1.26	2.18	1.83	3.18	1.46			0% (0.81)
302	U4/U6 small nuclear ribonucleoprotein Prp31 OS=Mus musculus GN=Prpf31 PE=2 SV=3	PRP31_MOUSE	55 kDa	6.15	3.74	8.9	3.74	1.45			0% (0.42)
303	Cleavage and polyadenylation specificity factor subunit 1 OS=Mus musculus GN=Cpsf1 PE=1 SV=1	CPSF1_MOUSE	161 kDa	6.91	0.65	9.94	4.32	1.44			0% (0.30)
304	Replication protein A 70 kDa DNA-binding subunit OS=Mus musculus GN=Rpa1 PE=2 SV=1	RFA1_MOUSE	69 kDa	11.22	6.29	16.08	6.35	1.43	RPA is a telomere-associated protein.	Verdun, Cell (2006) 127, 709-720.<br>Moser, EMBO J. (2009) 28, 810-820.<br>McGee, Nat. Struct. Mol. Biol. (2010) 17, 1438-1445.	0% (0.40)
305	Interleukin enhancer-binding factor 2 OS=Mus musculus GN=Ilf2 PE=1 SV=1	ILF2_MOUSE	43 kDa	4.33	1.71	6.2	2.43	1.43			0% (0.34)
306	Phospholipase A-2-activating protein OS=Mus musculus GN=Plaa PE=2 SV=4	PLAP_MOUSE	87 kDa	3.22	2.79	4.6	4	1.43			0% (0.65)
307	Polypyrimidine tract-binding protein 1 OS=Mus musculus GN=Ptbp1 PE=1 SV=2	PTBP1_MOUSE	56 kDa	39.99	11.84	56.66	1.5	1.42			0% (0.073)
308	RNA-binding protein 27 OS=Mus musculus GN=Rbm27 PE=2 SV=3	RBM27_MOUSE	119 kDa	5.33	1.75	7.5	2.07	1.41			0% (0.24)
309	U3 small nucleolar RNA-interacting protein 2 OS=Mus musculus GN=Rrp9 PE=1 SV=1	U3IP2_MOUSE	52 kDa	2.03	1.76	2.85	2.66	1.4			0% (0.68)
310	Cellular nucleic acid-binding protein OS=Mus musculus GN=Cnbp PE=2 SV=2	CNBP_MOUSE	20 kDa	1.62	1.5	2.24	2.26	1.38			0% (0.71)
311	DAZ-associated protein 1 OS=Mus musculus GN=Dazap1 PE=2 SV=2	DAZP1_MOUSE	43 kDa	3.88	3.54	5.34	4.69	1.38			0% (0.69)
312	Heterogeneous nuclear ribonucleoprotein F OS=Mus musculus GN=Hnrnpf PE=1 SV=3	HNRPF_MOUSE	46 kDa	51.3	14.42	70.22	18.83	1.37			0% (0.24)
313	U2 small nuclear ribonucleoprotein B'' OS=Mus musculus GN=Snrpb2 PE=2 SV=1	RU2B_MOUSE	25 kDa	3.51	3.8	4.77	4.97	1.36			0% (0.74)
314	Poly(rC)-binding protein 2 OS=Mus musculus GN=Pcbp2 PE=1 SV=1	PCBP2_MOUSE	38 kDa	21.87	8.92	29.69	4.85	1.36	PCBP1 binds to telomeric DNA *in vitro*.	Lacroix, Nucleic Acds Res. (2000) 28, 1564-1575.<br>Bandiera, Arch. Biochem. BIophys. (2003) 409, 350-314.	0% (0.25)
315	Cullin-associated NEDD8-dissociated protein 1 OS=Mus musculus GN=Cand1 PE=2 SV=2	CAND1_MOUSE	136 kDa	1.26	2.18	1.7	1.65	1.35			0% (0.79)
316	Fatty acid synthase OS=Mus musculus GN=Fasn PE=1 SV=2	FAS_MOUSE	272 kDa	19.38	3.69	26.02	3.39	1.34			0% (0.083)
317	Cleavage and polyadenylation specificity factor subunit 6 OS=Mus musculus GN=Cpsf6 PE=1 SV=1	CPSF6_MOUSE	59 kDa	7.64	1.52	10.23	2.98	1.34			0% (0.25)
318	Chloride intracellular channel protein 1 OS=Mus musculus GN=Clic1 PE=1 SV=3	CLIC1_MOUSE	27 kDa	7.82	3.25	10.44	7.65	1.33			0% (0.61)
319	Poly(rC)-binding protein 1 OS=Mus musculus GN=Pcbp1 PE=1 SV=1	PCBP1_MOUSE	37 kDa	25.78	5.2	34.28	6.98	1.33	PCBP1 binds to telomeric DNA *in vitro*.	Lacroix, Nucleic Acds Res. (2000) 28, 1564-1575.<br>Bandiera, Arch. Biochem. BIophys. (2003) 409, 350-314.	0% (0.17)
320	Splicing factor U2AF 35 kDa subunit OS=Mus musculus GN=U2af1 PE=1 SV=4	U2AF1_MOUSE	28 kDa	11.5	3.25	15.16	5.25	1.32			0% (0.36)
321	Nucleoprotein TPR OS=Mus musculus GN=Tpr PE=1 SV=1	TPR_MOUSE	0 kDa	17.47	0.69	22.98	1.29	1.32			95% (0.0028)
322	Heterogeneous nuclear ribonucleoprotein K OS=Mus musculus GN=Hnrnpk PE=1 SV=1	HNRPK_MOUSE	51 kDa	116.84	10.65	153.12	11.28	1.31			95% (0.015)
323	NEDD8-conjugating enzyme Ubc12 OS=Mus musculus GN=Ube2m PE=2 SV=1	UBC12_MOUSE	21 kDa	4.28	1.47	5.61	2.05	1.31			0% (0.41)
324	Calcium homeostasis endoplasmic reticulum protein OS=Mus musculus GN=Cherp PE=1 SV=1	CHERP_MOUSE	106 kDa	1.68	1.52	2.2	2.26	1.31			0% (0.76)
325	Proteasome subunit alpha type-7 OS=Mus musculus GN=Psma7 PE=1 SV=1	PSA7_MOUSE	28 kDa	0.69	1.2	0.9	1.56	1.3			0% (0.87)
326	Splicing factor, proline- and glutamine-rich OS=Mus musculus GN=Sfpq PE=1 SV=1	SFPQ_MOUSE	75 kDa	66.71	11.26	86.59	12.98	1.3			0% (0.12)
327	Heat shock protein HSP 90-beta OS=Mus musculus GN=Hsp90ab1 PE=1 SV=3	HS90B_MOUSE	83 kDa	57.29	10.15	74.21	8.03	1.3			0% (0.086)
328	RNA binding motif protein, X-linked-like-1 OS=Mus musculus GN=Rbmxl1 PE=1 SV=1	RMXL1_MOUSE	42 kDa	19.47	0.59	24.95	5.26	1.28			0% (0.15)
329	L-lactate dehydrogenase A chain OS=Mus musculus GN=Ldha PE=1 SV=3	LDHA_MOUSE	36 kDa	20.38	12.79	26.05	15.56	1.28			0% (0.65)
330	5'-3' exoribonuclease 2 OS=Mus musculus GN=Xrn2 PE=1 SV=1	XRN2_MOUSE	109 kDa	9	4.33	11.46	1.97	1.27			0% (0.42)
331	Polyadenylate-binding protein 1 OS=Mus musculus GN=Pabpc1 PE=1 SV=2	PABP1_MOUSE	71 kDa	12.41	6.5	15.8	16.39	1.27			0% (0.76)
332	Heterogeneous nuclear ribonucleoprotein D-like OS=Mus musculus GN=Hnrpdl PE=1 SV=1	HNRDL_MOUSE	34 kDa	7.99	13.84	10.16	17.6	1.27			0% (0.87)
333	Histone-binding protein RBBP4 OS=Mus musculus GN=Rbbp4 PE=1 SV=5	RBBP4_MOUSE	48 kDa	12.76	5.6	16.21	0.79	1.27			0% (0.35)
334	Heterogeneous nuclear ribonucleoprotein Q OS=Mus musculus GN=Syncrip PE=1 SV=2	HNRPQ_MOUSE	70 kDa	15.96	4.29	20.25	8.52	1.27			0% (0.48)
335	60S ribosomal protein L27 OS=Mus musculus GN=Rpl27 PE=2 SV=2	RL27_MOUSE	16 kDa	6.56	3.95	8.32	1.38	1.27			0% (0.51)
336	Intron-binding protein aquarius OS=Mus musculus GN=Aqr PE=2 SV=2	AQR_MOUSE	170 kDa	2.88	2.84	3.64	3.15	1.26			0% (0.77)
337	Nucleolysin TIAR OS=Mus musculus GN=Tial1 PE=2 SV=1	TIAR_MOUSE	43 kDa	2.34	2.48	2.95	2.69	1.26			0% (0.79)
338	tRNA (uracil-5-)-methyltransferase homolog A OS=Mus musculus GN=Trmt2a PE=2 SV=1	TRM2A_MOUSE	63 kDa	6.72	4.13	8.45	1.3	1.26			0% (0.52)
339	Peptidyl-prolyl cis-trans isomerase A OS=Mus musculus GN=Ppia PE=1 SV=2	PPIA_MOUSE	18 kDa	41.06	17.09	51.46	19.9	1.25			0% (0.53)
340	Protein KIAA1967 homolog OS=Mus musculus PE=1 SV=2	K1967_MOUSE	103 kDa	6.59	0.34	8.24	2.6	1.25			0% (0.34)
341	Host cell factor 1 OS=Mus musculus GN=Hcfc1 PE=1 SV=2	HCFC1_MOUSE	210 kDa	9.63	4.36	12.01	1.89	1.25			0% (0.43)
342	Heterogeneous nuclear ribonucleoproteins A2/B1 OS=Mus musculus GN=Hnrnpa2b1 PE=1 SV=2	ROA2_MOUSE	37 kDa	104.75	10.1	130.22	22.71	1.24			0% (0.15)
343	mRNA turnover protein 4 homolog OS=Mus musculus GN=Mrto4 PE=2 SV=1	MRT4_MOUSE	28 kDa	4.28	0.39	5.32	0.96	1.24			0% (0.16)
344	T-complex protein 1 subunit beta OS=Mus musculus GN=Cct2 PE=1 SV=4	TCPB_MOUSE	57 kDa	2.83	4.91	3.5	3.61	1.24			0% (0.86)
345	Protein DJ-1 OS=Mus musculus GN=Park7 PE=1 SV=1	PARK7_MOUSE	20 kDa	3.24	2.1	4.01	0.63	1.24			0% (0.58)
346	RNA-binding protein 14 OS=Mus musculus GN=Rbm14 PE=1 SV=1	RBM14_MOUSE	69 kDa	36.86	6.78	45.49	9.22	1.23			0% (0.26)
347	Serine/arginine-rich splicing factor 10 OS=Mus musculus GN=Srsf10 PE=1 SV=2	SRS10_MOUSE	31 kDa	6.1	4.89	7.53	5.32	1.23			0% (0.75)
348	Heterogeneous nuclear ribonucleoprotein H OS=Mus musculus GN=Hnrnph1 PE=1 SV=3	HNRH1_MOUSE	49 kDa	42.44	16.01	52.25	6.54	1.23			0% (0.38)
349	Tubulin alpha-1B chain OS=Mus musculus GN=Tuba1b PE=1 SV=2	TBA1B_MOUSE	50 kDa	21.45	4.88	26.37	2.03	1.23			0% (0.18)
350	Heterogeneous nuclear ribonucleoprotein U-like protein 1 OS=Mus musculus GN=Hnrnpul1 PE=1 SV=1	HNRL1_MOUSE	96 kDa	6.98	2.24	8.58	3.68	1.23			0% (0.56)
351	Heterogeneous nuclear ribonucleoprotein L OS=Mus musculus GN=Hnrnpl PE=1 SV=2	HNRPL_MOUSE	64 kDa	45.97	13.1	56.37	16.17	1.23			0% (0.44)
352	RNA-binding protein FUS OS=Mus musculus GN=Fus PE=2 SV=1	FUS_MOUSE	53 kDa	16.02	6.98	19.63	7.37	1.23			0% (0.57)
353	Heterogeneous nuclear ribonucleoproteins C1/C2 OS=Mus musculus GN=Hnrnpc PE=1 SV=1	HNRPC_MOUSE	34 kDa	43.73	6.53	53.27	6.64	1.22			0% (0.15)
354	RNA-binding protein EWS OS=Mus musculus GN=Ewsr1 PE=1 SV=2	EWS_MOUSE	68 kDa	5.62	1.16	6.84	1.76	1.22			0% (0.37)
355	RNA-binding protein Raly OS=Mus musculus GN=Raly PE=1 SV=3	RALY_MOUSE	33 kDa	23.32	7.14	28.37	3.66	1.22			0% (0.34)
356	DNA damage-binding protein 1 OS=Mus musculus GN=Ddb1 PE=1 SV=2	DDB1_MOUSE	127 kDa	13.7	5.55	16.66	9.52	1.22			0% (0.67)
357	40S ribosomal protein S13 OS=Mus musculus GN=Rps13 PE=1 SV=2	RS13_MOUSE	17 kDa	5.59	0.9	6.77	4.46	1.21			0% (0.68)
358	Heterogeneous nuclear ribonucleoprotein A1 OS=Mus musculus GN=Hnrnpa1 PE=1 SV=2	ROA1_MOUSE	34 kDa	94.1	23.26	112.34	13.01	1.19			0% (0.30)
359	Probable ATP-dependent RNA helicase DDX5 OS=Mus musculus GN=Ddx5 PE=1 SV=2	DDX5_MOUSE	69 kDa	75.19	31.5	89.35	41.05	1.19			0% (0.66)
360	Triosephosphate isomerase OS=Mus musculus GN=Tpi1 PE=1 SV=4	TPIS_MOUSE	32 kDa	9.15	4.84	10.87	7.66	1.19			0% (0.76)
361	Ribosome production factor 2 homolog OS=Mus musculus GN=Rpf2 PE=2 SV=2	RPF2_MOUSE	35 kDa	6.59	5.44	7.81	4.33	1.19			0% (0.78)
362	Interleukin enhancer-binding factor 3 OS=Mus musculus GN=Ilf3 PE=1 SV=2	ILF3_MOUSE	96 kDa	6.86	7.99	8.11	9.75	1.18			0% (0.87)
363	Heat shock protein HSP 90-alpha OS=Mus musculus GN=Hsp90aa1 PE=1 SV=4	HS90A_MOUSE	85 kDa	57.9	7.62	68.42	6.99	1.18			0% (0.15)
364	Probable ATP-dependent RNA helicase DDX17 OS=Mus musculus GN=Ddx17 PE=2 SV=1	DDX17_MOUSE	72 kDa	45.82	18.81	54.09	35.15	1.18			0% (0.74)
365	Putative pre-mRNA-splicing factor ATP-dependent RNA helicase DHX15 OS=Mus musculus GN=Dhx15 PE=2 SV=	DHX15_MOUSE	91 kDa	26.3	3.41	31.04	6.61	1.18			0% (0.33)
366	Small nuclear ribonucleoprotein Sm D1 OS=Mus musculus GN=Snrpd1 PE=2 SV=1	SMD1_MOUSE (+3)	13 kDa	7.89	1.36	9.29	5.37	1.18			0% (0.68)
367	ATP-dependent RNA helicase DDX1 OS=Mus musculus GN=Ddx1 PE=1 SV=1	DDX1_MOUSE	83 kDa	8.01	4.29	9.41	3.03	1.18			0% (0.67)
368	Pre-mRNA-processing factor 40 homolog A OS=Mus musculus GN=Prpf40a PE=1 SV=1	PR40A_MOUSE	108 kDa	3.87	3.35	4.54	4.14	1.17			0% (0.84)
369	Putative ribosomal RNA methyltransferase NOP2 OS=Mus musculus GN=Nop2 PE=2 SV=1	NOP2_MOUSE	87 kDa	14.53	10.45	17.02	16.38	1.17			0% (0.84)
370	Adenosylhomocysteinase OS=Mus musculus GN=Ahcy PE=1 SV=3	SAHH_MOUSE	48 kDa	43.81	13.18	51.16	19.18	1.17			0% (0.61)
371	Fructose-bisphosphate aldolase A OS=Mus musculus GN=Aldoa PE=1 SV=2	ALDOA_MOUSE	39 kDa	15.66	5.7	18.28	6.84	1.17			0% (0.64)
372	ATP-binding cassette sub-family E member 1 OS=Mus musculus GN=Abce1 PE=2 SV=1	ABCE1_MOUSE	67 kDa	0.94	1.63	1.1	1.91	1.17			0% (0.92)
373	Metastasis-associated protein MTA1 OS=Mus musculus GN=Mta1 PE=1 SV=1	MTA1_MOUSE	81 kDa	6.13	5.33	7.14	6.47	1.17			0% (0.84)
374	Multifunctional protein ADE2 OS=Mus musculus GN=Paics PE=1 SV=4	PUR6_MOUSE	47 kDa	0.63	1.09	0.73	1.27	1.16			0% (0.92)
375	CAD protein OS=Mesocricetus auratus GN=CAD PE=1 SV=4	PYR1_MESAU	243 kDa	0.63	1.09	0.73	1.27	1.16			0% (0.92)
376	Coatomer subunit beta' OS=Mus musculus GN=Copb2 PE=2 SV=2	COPB2_MOUSE	102 kDa	0.63	1.09	0.73	1.27	1.16			0% (0.92)
377	Integrator complex subunit 2 OS=Mus musculus GN=Ints2 PE=2 SV=2	INT2_MOUSE	134 kDa	0.63	1.09	0.73	1.27	1.16			0% (0.92)
378	Non-POU domain-containing octamer-binding protein OS=Mus musculus GN=Nono PE=1 SV=3	NONO_MOUSE	55 kDa	39.35	3.04	45.79	12.28	1.16			0% (0.43)
379	Transgelin-2 OS=Mus musculus GN=Tagln2 PE=1 SV=4	TAGL2_MOUSE	22 kDa	6.68	7.7	7.74	7.27	1.16			0% (0.87)
380	Nucleophosmin OS=Mus musculus GN=Npm1 PE=1 SV=1	NPM_MOUSE	33 kDa	34.73	3.52	40.21	7.9	1.16			0% (0.33)
381	DNA primase large subunit OS=Mus musculus GN=Prim2 PE=1 SV=1	PRI2_MOUSE	58 kDa	1.67	1.58	1.93	1.81	1.16			0% (0.86)
382	Nucleolar transcription factor 1 OS=Mus musculus GN=Ubtf PE=1 SV=1	UBF1_MOUSE	90 kDa	12.16	2.73	14.03	2.34	1.15			0% (0.42)
383	Serine/arginine-rich splicing factor 5 OS=Mus musculus GN=Srsf5 PE=1 SV=1	SRSF5_MOUSE	31 kDa	10.17	8.92	11.66	5.55	1.15			0% (0.82)
384	Splicing factor U2AF 65 kDa subunit OS=Mus musculus GN=U2af2 PE=1 SV=3	U2AF2_MOUSE	54 kDa	7.96	12.11	9.11	10.79	1.15			0% (0.91)
385	GMP synthase [glutamine-hydrolyzing] OS=Mus musculus GN=Gmps PE=1 SV=2	GUAA_MOUSE	77 kDa	14.81	6.18	16.94	1.69	1.14			0% (0.59)
386	Far upstream element-binding protein 2 OS=Mus musculus GN=Khsrp PE=1 SV=2	FUBP2_MOUSE	77 kDa	38.48	5.91	43.98	7.73	1.14			0% (0.38)
387	Heterogeneous nuclear ribonucleoprotein A3 OS=Mus musculus GN=Hnrnpa3 PE=1 SV=1	ROA3_MOUSE	40 kDa	80.02	35.22	91.43	41.95	1.14			0% (0.74)
388	Serine/arginine-rich splicing factor 2 OS=Mus musculus GN=Srsf2 PE=1 SV=4	SRSF2_MOUSE	25 kDa	11.54	1.23	13.12	5.24	1.14			0% (0.64)
389	RNA-binding protein 28 OS=Mus musculus GN=Rbm28 PE=1 SV=4	RBM28_MOUSE	84 kDa	8.41	4.9	9.57	12.86	1.14			0% (0.89)
390	Poly(U)-binding-splicing factor PUF60 OS=Mus musculus GN=Puf60 PE=2 SV=2	PUF60_MOUSE	60 kDa	2.65	0.6	3	0.79	1.13			0% (0.57)
391	Pleiotropic regulator 1 OS=Mus musculus GN=Plrg1 PE=2 SV=1	PLRG1_MOUSE	57 kDa	2.56	2.39	2.9	2.73	1.13			0% (0.88)
392	Elongation factor 2 OS=Mus musculus GN=Eef2 PE=1 SV=2	EF2_MOUSE	95 kDa	55.95	16.87	63.29	21.31	1.13			0% (0.66)
393	Sister chromatid cohesion protein PDS5 homolog A OS=Mus musculus GN=Pds5a PE=2 SV=3	PDS5A_MOUSE	150 kDa	5.64	1.67	6.36	3.08	1.13			0% (0.74)
394	D-3-phosphoglycerate dehydrogenase OS=Mus musculus GN=Phgdh PE=1 SV=3	SERA_MOUSE	57 kDa	7.55	3.94	8.49	5.19	1.12			0% (0.81)
395	RNA-binding protein 10 OS=Mus musculus GN=Rbm10 PE=1 SV=1	RBM10_MOUSE	103 kDa	1.74	3.01	1.95	1.7	1.12			0% (0.92)
396	Small ubiquitin-related modifier 1 OS=Mus musculus GN=Sumo1 PE=1 SV=1	SUMO1_MOUSE	12 kDa	3.97	0.2	4.45	1.88	1.12			0% (0.68)
397	S-methyl-5'-thioadenosine phosphorylase OS=Mus musculus GN=Mtap PE=2 SV=1	MTAP_MOUSE	31 kDa	8.78	4.9	9.84	1.97	1.12			0% (0.74)
398	Heterogeneous nuclear ribonucleoprotein D0 OS=Mus musculus GN=Hnrnpd PE=1 SV=2	HNRPD_MOUSE	38 kDa	23.58	4.94	26.39	8.15	1.12			0% (0.64)
399	Cofilin-1 OS=Mus musculus GN=Cfl1 PE=1 SV=3	COF1_MOUSE	19 kDa	18.19	9.01	20.26	7.68	1.11			0% (0.78)
400	WD repeat-containing protein 36 OS=Homo sapiens GN=WDR36 PE=1 SV=1	WDR36_HUMAN	105 kDa	0.66	1.14	0.73	1.27	1.11			0% (0.94)
401	Phosphoserine aminotransferase OS=Mus musculus GN=Psat1 PE=1 SV=1	SERC_MOUSE	40 kDa	0.66	1.14	0.73	1.27	1.11			0% (0.94)
402	Pescadillo homolog OS=Mus musculus GN=Pes1 PE=1 SV=1	PESC_MOUSE	68 kDa	13.75	3.29	15.28	5.24	1.11			0% (0.69)
403	Heterogeneous nuclear ribonucleoprotein A/B OS=Mus musculus GN=Hnrnpab PE=1 SV=1	ROAA_MOUSE	31 kDa	31.86	5.17	35.38	10.77	1.11			0% (0.64)
404	60S ribosomal protein L12 OS=Mus musculus GN=Rpl12 PE=1 SV=2	RL12_MOUSE	18 kDa	13.37	6.23	14.71	7.33	1.1			0% (0.82)
405	Serrate RNA effector molecule homolog OS=Mus musculus GN=Srrt PE=1 SV=1	SRRT_MOUSE	100 kDa	15.82	1.1	17.39	3.27	1.1			0% (0.47)
406	Probable rRNA-processing protein EBP2 OS=Mus musculus GN=Ebna1bp2 PE=2 SV=1	EBP2_MOUSE	35 kDa	2.69	2.5	2.95	2.69	1.1			0% (0.91)
407	Flap endonuclease 1 OS=Mus musculus GN=Fen1 PE=1 SV=1	FEN1_MOUSE	42 kDa	8.58	2	9.4	0.86	1.1			0% (0.54)
408	Thymidylate synthase OS=Mus musculus GN=Tyms PE=1 SV=1	TYSY_MOUSE	35 kDa	2.94	0.85	3.21	2.84	1.09			0% (0.89)
409	Acidic leucine-rich nuclear phosphoprotein 32 family member A OS=Mus musculus GN=Anp32a PE=1 SV=1	AN32A_MOUSE	29 kDa	16.99	6.66	18.4	5.91	1.08			0% (0.80)
410	60S ribosomal protein L34 OS=Mus musculus GN=Rpl34 PE=3 SV=2	RL34_MOUSE	13 kDa	5.48	4.8	5.94	5.14	1.08			0% (0.92)
411	KH domain-containing, RNA-binding, signal transduction-associated protein 1 OS=Mus musculus GN=Khdrbs1 PE=	KHDR1_MOUSE	48 kDa	11.52	0.98	12.41	1.65	1.08			0% (0.47)
412	Heterogeneous nuclear ribonucleoprotein U OS=Mus musculus GN=Hnrnpu PE=1 SV=1	HNRPU_MOUSE	88 kDa	105.12	39.84	113.1	36.72	1.08			0% (0.81)
413	Pyruvate kinase isozymes M1/M2 OS=Mus musculus GN=Pkm2 PE=1 SV=4	KPYM_MOUSE	58 kDa	42.47	14.38	45.65	23.87	1.07			0% (0.85)
414	Protein RRP5 homolog OS=Mus musculus GN=Pdcd11 PE=2 SV=2	RRP5_MOUSE	208 kDa	21.16	8.33	22.65	2.33	1.07			0% (0.78)
415	Transketolase OS=Mus musculus GN=Tkt PE=1 SV=1	TKT_MOUSE	68 kDa	39.65	3.66	42.42	13.07	1.07			0% (0.74)
416	U2 snRNP-associated SURP motif-containing protein OS=Mus musculus GN=U2surp PE=1 SV=3	SR140_MOUSE	118 kDa	10.03	4.86	10.73	2.99	1.07			0% (0.84)
417	Heat shock cognate 71 kDa protein OS=Mus musculus GN=Hspa8 PE=1 SV=1	HSP7C_MOUSE	71 kDa	77.4	22.76	82.59	6.81	1.07			0% (0.72)
418	U6 snRNA-associated Sm-like protein LSm6 OS=Mus musculus GN=Lsm6 PE=2 SV=1	LSM6_MOUSE	9 kDa	3.19	3.31	3.4	3.3	1.06			0% (0.94)
419	Pumilio domain-containing protein KIAA0020 OS=Mus musculus GN=Kiaa0020 PE=2 SV=2	K0020_MOUSE	73 kDa	10.61	1.84	11.28	6.63	1.06			0% (0.87)
420	Ribosomal RNA processing protein 1 homolog B OS=Mus musculus GN=Rrp1b PE=2 SV=2	RRP1B_MOUSE	81 kDa	0.99	1.71	1.05	1.82	1.06			0% (0.97)
421	FACT complex subunit SPT16 OS=Mus musculus GN=Supt16h PE=1 SV=2	SP16H_MOUSE	120 kDa	39.19	7.41	41.48	6.98	1.06			0% (0.72)
422	Putative methyltransferase NSUN5 OS=Mus musculus GN=Nsun5 PE=2 SV=2	NSUN5_MOUSE	51 kDa	0.69	1.2	0.73	1.27	1.06			0% (0.97)
423	ATP-dependent RNA helicase A OS=Mus musculus GN=Dhx9 PE=1 SV=2	DHX9_MOUSE	149 kDa	106.85	11.35	112.63	22.43	1.05			0% (0.71)
424	U4/U6.U5 tri-snRNP-associated protein 1 OS=Mus musculus GN=Sart1 PE=2 SV=1	SNUT1_MOUSE	91 kDa	1.62	1.5	1.7	1.65	1.05			0% (0.95)
425	Nucleolin OS=Mus musculus GN=Ncl PE=1 SV=2	NUCL_MOUSE	77 kDa	112.52	31.01	117.98	49.11	1.05			0% (0.88)
426	Pre-mRNA-processing factor 19 OS=Mus musculus GN=Prpf19 PE=2 SV=1	PRP19_MOUSE	55 kDa	13.91	2.91	14.58	2.65	1.05			0% (0.78)
427	Far upstream element-binding protein 1 OS=Mus musculus GN=Fubp1 PE=1 SV=1	FUBP1_MOUSE	69 kDa	31.71	12.25	33.09	16.35	1.04			0% (0.91)
428	Proliferating cell nuclear antigen OS=Mus musculus GN=Pcna PE=1 SV=2	PCNA_MOUSE	29 kDa	13.12	3.34	13.65	9.14	1.04			0% (0.93)
429	Protein FAM50A OS=Mus musculus GN=Fam50a PE=2 SV=1	FA50A_MOUSE	40 kDa	3.52	3.33	3.64	3.15	1.03			0% (0.97)
430	ELAV-like protein 1 OS=Mus musculus GN=Elavl1 PE=1 SV=2	ELAV1_MOUSE	36 kDa	24.21	5.91	24.85	4.83	1.03			0% (0.89)
431	Transducin beta-like protein 3 OS=Mus musculus GN=Tbl3 PE=2 SV=1	TBL3_MOUSE	88 kDa	5.75	4.32	5.89	3.4	1.02			0% (0.97)
432	WD repeat-containing protein 3 OS=Mus musculus GN=Wdr3 PE=2 SV=1	WDR3_MOUSE	106 kDa	6.65	1.47	6.79	2.31	1.02			0% (0.93)
433	RNA-binding protein 12 OS=Mus musculus GN=Rbm12 PE=1 SV=3	RBM12_MOUSE	103 kDa	2.33	2.13	2.38	0.69	1.02			0% (0.97)
434	Probable ATP-dependent RNA helicase DDX27 OS=Mus musculus GN=Ddx27 PE=1 SV=3	DDX27_MOUSE	86 kDa	7.07	4.55	7.21	10.24	1.02			0% (0.98)
435	Serine/arginine-rich splicing factor 4 OS=Mus musculus GN=Srsf4 PE=2 SV=1	SRSF4_MOUSE	56 kDa	15.06	5	15.31	3.91	1.02			0% (0.95)
436	Transcription activator BRG1 OS=Mus musculus GN=Smarca4 PE=1 SV=1	SMCA4_MOUSE	181 kDa	6.47	4.31	6.54	2.99	1.01			0% (0.98)
437	60S ribosomal protein L38 OS=Mus musculus GN=Rpl38 PE=2 SV=3	RL38_MOUSE	8 kDa	0.69	1.2	0.7	1.21	1.01			0% (1.00)

438	CWF19-like protein 1 OS=Mus musculus GN=Cwf19l1 PE=2 SV=2	C19L1_MOUSE	60 kDa	0.69	1.2	0.7	1.21	1.01			0% (1.00)
439	Ig kappa chain V-V region K2 (Fragment) OS=Mus musculus PE=1 SV=1	KV5A3_MOUSE	13 kDa	0.69	1.2	0.7	1.21	1.01			0% (1.00)
440	60S ribosomal protein L22 OS=Mus musculus GN=Rpl22 PE=2 SV=2	RL22_MOUSE	15 kDa	0.69	1.2	0.7	1.21	1.01			0% (1.00)
441	Small nuclear ribonucleoprotein E OS=Mus musculus GN=Snrpe PE=2 SV=1	RUXE_MOUSE (+4)	11 kDa	0.69	1.2	0.7	1.21	1.01			0% (1.00)
442	Spermidine synthase OS=Mus musculus GN=Srm PE=2 SV=1	SPEE_MOUSE	34 kDa	0.69	1.2	0.7	1.21	1.01			0% (1.00)
443	MORC family CW-type zinc finger protein 2A OS=Mus musculus GN=Morc2a PE=1 SV=2	MOR2A_MOUSE	117 kDa	1.74	3.01	1.75	3.04	1.01			0% (1.00)
444	Heterogeneous nuclear ribonucleoprotein R OS=Homo sapiens GN=HNRNPR PE=1 SV=1	HNRPR_HUMAN	71 kDa	1.74	3.01	1.75	3.04	1.01			0% (1.00)
445	40S ribosomal protein S25 OS=Mus musculus GN=Rps25 PE=2 SV=1	RS25_MOUSE	14 kDa	1.39	2.41	1.4	2.43	1.01			0% (1.00)
446	Ribosome biogenesis protein BRX1 homolog OS=Mus musculus GN=Brix1 PE=2 SV=3	BRX1_MOUSE	41 kDa	1.04	1.81	1.05	1.82	1.01			0% (1.00)
447	Transcription elongation factor SPT6 OS=Mus musculus GN=Supt6h PE=1 SV=2	SPT6H_MOUSE	199 kDa	1.04	1.81	1.05	1.82	1.01			0% (1.00)
448	Ribosome biogenesis protein NSA2 homolog OS=Mus musculus GN=Nsa2 PE=2 SV=1	NSA2_MOUSE	30 kDa	1.04	1.81	1.05	1.82	1.01			0% (1.00)
449	Kinesin-like protein KIF20A OS=Mus musculus GN=Kif20a PE=2 SV=1	KI20A_MOUSE	100 kDa	1.04	1.81	1.05	1.82	1.01			0% (1.00)
450	Protein arginine N-methyltransferase 1 OS=Mus musculus GN=Prmt1 PE=1 SV=1	ANM1_MOUSE	42 kDa	20.31	5.57	20.43	4	1.01			0% (0.98)
451	Elongation factor 1-alpha 1 OS=Mus musculus GN=Eef1a1 PE=1 SV=3	EF1A1_MOUSE	50 kDa	86.79	23.72	87.26	32.99	1.01			0% (0.99)
452	Crooked neck-like protein 1 OS=Mus musculus GN=Crnkl1 PE=2 SV=1	CRNL1_MOUSE	83 kDa	3.62	0.44	3.64	3.15	1			0% (0.99)
453	Bifunctional purine biosynthesis protein PURH OS=Mus musculus GN=Atic PE=1 SV=2	PUR9_MOUSE	64 kDa	2.83	4.91	2.84	3.2	1			0% (1.00)
454	Cell division cycle 5-like protein OS=Mus musculus GN=Cdc5l PE=1 SV=2	CDC5L_MOUSE	92 kDa	8.23	0.83	8.24	2.66	1			0% (1.00)
455	Nucleoside diphosphate kinase B OS=Mus musculus GN=Nme2 PE=1 SV=1	NDKB_MOUSE	17 kDa	8.66	7.8	8.67	7.62	1			0% (1.00)
456	Zinc finger RNA-binding protein OS=Mus musculus GN=Zfr PE=1 SV=2	ZFR_MOUSE	117 kDa	5.7	2.76	5.66	5.79	0.99			0% (0.99)
457	Guanine nucleotide-binding protein-like 3 OS=Mus musculus GN=Gnl3 PE=1 SV=2	GNL3_MOUSE	61 kDa	14.44	7.65	14.28	9.55	0.99			0% (0.98)
458	DNA replication licensing factor MCM6 OS=Mus musculus GN=Mcm6 PE=1 SV=1	MCM6_MOUSE	93 kDa	37.1	7.13	36.65	15.36	0.99			0% (0.97)
459	High mobility group protein B2 OS=Mus musculus GN=Hmgb2 PE=1 SV=3	HMGB2_MOUSE	24 kDa	26.29	3.68	25.8	5.04	0.98			0% (0.90)
460	DNA mismatch repair protein Msh6 OS=Mus musculus GN=Msh6 PE=1 SV=3	MSH6_MOUSE	151 kDa	10.32	2.81	10.11	0.36	0.98			0% (0.90)
461	Histone deacetylase 1 OS=Mus musculus GN=Hdac1 PE=1 SV=1	HDAC1_MOUSE	55 kDa	7.95	2.78	7.77	0.6	0.98			0% (0.92)
462	60S ribosomal protein L35a OS=Mus musculus GN=Rpl35a PE=2 SV=2	RL35A_MOUSE	13 kDa	7.54	2.02	7.36	0.76	0.98			0% (0.89)
463	Small nuclear ribonucleoprotein Sm D2 OS=Mus musculus GN=Snrpd2 PE=2 SV=1	SMD2_MOUSE	14 kDa	13.93	10.44	13.56	7.29	0.97			0% (0.96)
464	Cytosolic purine 5'-nucleotidase OS=Mus musculus GN=Nt5c2 PE=1 SV=2	5NTC_MOUSE	65 kDa	3.09	3.78	3	3.17	0.97			0% (0.98)
465	T-complex protein 1 subunit eta OS=Mus musculus GN=Cct7 PE=1 SV=1	TCPH_MOUSE	60 kDa	3.15	5.45	3.03	2.79	0.96			0% (0.98)
466	DNA replication licensing factor MCM3 OS=Mus musculus GN=Mcm3 PE=1 SV=2	MCM3_MOUSE	92 kDa	37.35	6.83	35.92	3.57	0.96			0% (0.76)
467	Structural maintenance of chromosomes protein 2 OS=Mus musculus GN=Smc2 PE=1 SV=2	SMC2_MOUSE	134 kDa	8	3.02	7.69	2.11	0.96			0% (0.89)
468	tRNA (cytosine(34)-C(5))-methyltransferase OS=Mus musculus GN=Nsun2 PE=1 SV=2	NSUN2_MOUSE	85 kDa	10.6	1.81	10.14	1.85	0.96			0% (0.77)
469	Myb-binding protein 1A OS=Mus musculus GN=Mybbp1a PE=1 SV=2	MBB1A_MOUSE	152 kDa	86.95	21.89	83.14	20.38	0.96			0% (0.84)
470	DNA primase small subunit OS=Mus musculus GN=Prim1 PE=1 SV=1	PRI1_MOUSE	49 kDa	0.94	1.63	0.9	1.56	0.95			0% (0.98)
471	Polycomb protein Suz12 OS=Mus musculus GN=Suz12 PE=1 SV=2	SUZ12_MOUSE	83 kDa	0.94	1.63	0.9	1.56	0.95			0% (0.98)
472	Peptidylprolyl isomerase domain and WD repeat-containing protein 1 OS=Mus musculus GN=Ppwd1 PE=2 SV=2	PPWD1_MOUSE	73 kDa	0.63	1.09	0.6	1.04	0.95			0% (0.98)
473	DnaJ homolog subfamily C member 8 OS=Mus musculus GN=Dnajc8 PE=2 SV=2	DNJC8_MOUSE	30 kDa	0.63	1.09	0.6	1.04	0.95			0% (0.98)
474	Protein ELYS OS=Mus musculus GN=Ahctf1 PE=1 SV=1	ELYS_MOUSE	248 kDa	0.63	1.09	0.6	1.04	0.95			0% (0.98)
475	Cleavage and polyadenylation specificity factor subunit 5 OS=Mus musculus GN=Nudt21 PE=2 SV=1	CPSF5_MOUSE	26 kDa	6.49	3.83	6.15	3.43	0.95			0% (0.91)
476	Aspartate--tRNA ligase, cytoplasmic OS=Mus musculus GN=Dars PE=2 SV=2	SYDC_MOUSE	57 kDa	2.92	2.97	2.73	2.75	0.94			0% (0.94)
477	Ig kappa chain C region OS=Mus musculus PE=1 SV=1	IGKC_MOUSE	12 kDa	79.43	13.76	74.21	8.32	0.93			0% (0.60)
478	Splicing factor 3B subunit 1 OS=Mus musculus GN=Sf3b1 PE=1 SV=1	SF3B1_MOUSE	146 kDa	16.23	10.55	15.14	9.66	0.93			0% (0.90)
479	Transcription elongation factor SPT5 OS=Mus musculus GN=Supt5h PE=1 SV=1	SPT5H_MOUSE	121 kDa	7.54	1.68	6.97	2.48	0.93			0% (0.76)
480	Eukaryotic initiation factor 4A-I OS=Mus musculus GN=Eif4a1 PE=2 SV=1	IF4A1_MOUSE	46 kDa	20.58	7.04	18.76	6.27	0.91			0% (0.76)
481	Proteasome subunit alpha type-6 OS=Mus musculus GN=Psma6 PE=1 SV=1	PSA6_MOUSE	27 kDa	1.32	2.29	1.2	2.08	0.91			0% (0.95)
482	Platelet-activating factor acetylhydrolase IB subunit alpha OS=Mus musculus GN=Pafah1b1 PE=1 SV=2	LIS1_MOUSE	47 kDa	3.22	2.79	2.9	2.73	0.9			0% (0.89)
483	DNA replication licensing factor MCM5 OS=Mus musculus GN=Mcm5 PE=2 SV=1	MCM5_MOUSE	82 kDa	34.88	4.22	31.27	6.31	0.9			0% (0.46)
484	Luc7-like protein 3 OS=Mus musculus GN=Luc7l3 PE=1 SV=1	LC7L3_MOUSE	51 kDa	3.69	1.68	3.3	3.17	0.9			0% (0.86)
485	Serine/arginine-rich splicing factor 1 OS=Mus musculus GN=Srsf1 PE=1 SV=3	SRSF1_MOUSE	28 kDa	35.1	8.08	31.4	3.37	0.89			0% (0.51)
486	40S ribosomal protein S17 OS=Mus musculus GN=Rps17 PE=1 SV=2	RS17_MOUSE	16 kDa	6.11	5.3	5.47	5.5	0.89			0% (0.89)
487	Protein mago nashi homolog OS=Mus musculus GN=Magoh PE=2 SV=1	MGN_MOUSE	17 kDa	3.66	4.66	3.27	2.88	0.89			0% (0.91)
488	Keratin, type II cytoskeletal 6A OS=Mus musculus GN=Krt6a PE=2 SV=3	K2C6A_MOUSE	59 kDa	5.91	10.23	5.26	9.1	0.89			0% (0.94)
489	Lymphocyte-specific helicase OS=Mus musculus GN=Hells PE=1 SV=2	HELLS_MOUSE	95 kDa	15.41	3.23	13.71	1.42	0.89			0% (0.45)
490	Regulator of chromosome condensation OS=Mus musculus GN=Rcc1 PE=1 SV=1	RCC1_MOUSE	45 kDa	4.16	3.83	3.7	3.37	0.89			0% (0.88)
491	Probable ATP-dependent RNA helicase DDX46 OS=Mus musculus GN=Ddx46 PE=1 SV=2	DDX46_MOUSE	117 kDa	21.99	6.06	19.49	3.29	0.89			0% (0.56)
492	Actin, cytoplasmic 1 OS=Mus musculus GN=Actb PE=1 SV=1	ACTB_MOUSE (+1)	42 kDa	69.44	5.59	61.35	14	0.88			0% (0.41)
493	60S ribosomal protein L14 OS=Mus musculus GN=Rpl14 PE=2 SV=3	RL14_MOUSE	24 kDa	7.1	6.27	6.27	3.67	0.88			0% (0.85)
494	High mobility group protein B1 OS=Mus musculus GN=Hmgb1 PE=1 SV=2	HMGB1_MOUSE	25 kDa	28.84	6.93	25.45	5.64	0.88			0% (0.55)
495	Putative RNA-binding protein Luc7-like 2 OS=Mus musculus GN=Luc7l2 PE=1 SV=1	LC7L2_MOUSE	47 kDa	8.19	4.38	7.22	3.85	0.88			0% (0.79)
496	60S ribosomal protein L30 OS=Mus musculus GN=Rpl30 PE=2 SV=2	RL30_MOUSE	13 kDa	2.96	0.95	2.6	2.28	0.88			0% (0.82)
497	Lupus La protein homolog OS=Mus musculus GN=Ssb PE=2 SV=1	LA_MOUSE	48 kDa	24.1	1.2	21.12	4.4	0.88			0% (0.32)
498	Histone-binding protein RBBP7 OS=Mus musculus GN=Rbbp7 PE=1 SV=1	RBBP7_MOUSE	48 kDa	10.19	3.16	8.9	7.71	0.87			0% (0.80)
499	Ig kappa chain V-II region 26-10 OS=Mus musculus PE=1 SV=1	KV2A7_MOUSE	12 kDa	49.43	13.26	43.17	10.12	0.87			0% (0.55)
500	60S ribosomal protein L21 OS=Mus musculus GN=Rpl21 PE=2 SV=3	RL21_MOUSE	19 kDa	3.84	3.78	3.34	1.03	0.87			0% (0.84)
501	Chromobox protein homolog 5 OS=Mus musculus GN=Cbx5 PE=1 SV=1	CBX5_MOUSE	22 kDa	6.15	3.74	5.34	4.69	0.87			0% (0.83)
502	Structural maintenance of chromosomes protein 4 OS=Mus musculus GN=Smc4 PE=1 SV=1	SMC4_MOUSE	147 kDa	19.67	4.42	17.08	5.7	0.87			0% (0.57)
503	Small nuclear ribonucleoprotein Sm D3 OS=Mus musculus GN=Snrpd3 PE=1 SV=1	SMD3_MOUSE	14 kDa	9.09	5.17	7.89	4.35	0.87			0% (0.77)
504	Small ubiquitin-related modifier 2 OS=Mus musculus GN=Sumo2 PE=2 SV=1	SUMO2_MOUSE	11 kDa	14.24	4.24	12.34	4.36	0.87			0% (0.62)
505	E3 ubiquitin-protein ligase UHRF1 OS=Mus musculus GN=Uhrf1 PE=1 SV=2	UHRF1_MOUSE	88 kDa	12.06	5.04	10.44	1.87	0.87			0% (0.63)
506	Serine/arginine-rich splicing factor 3 OS=Mus musculus GN=Srsf3 PE=1 SV=1	SRSF3_MOUSE	19 kDa	10.66	4.63	9.21	4.77	0.86			0% (0.73)
507	Ubiquitin carboxyl-terminal hydrolase 7 OS=Mus musculus GN=Usp7 PE=1 SV=1	UBP7_MOUSE	128 kDa	1.04	1.81	0.9	1.56	0.86			0% (0.92)
508	Heterogeneous nuclear ribonucleoprotein U-like protein 2 OS=Mus musculus GN=Hnrnpul2 PE=1 SV=1	HNRL2_MOUSE	85 kDa	19.81	1.74	16.98	2.32	0.86			0% (0.17)
509	FACT complex subunit SSRP1 OS=Mus musculus GN=Ssrp1 PE=1 SV=2	SSRP1_MOUSE	81 kDa	13.79	9.8	11.67	12.71	0.85			0% (0.83)
510	60S ribosomal protein L23 OS=Mus musculus GN=Rpl23 PE=1 SV=1	RL23_MOUSE	15 kDa	5.19	2.81	4.39	1.18	0.85			0% (0.67)
511	DNA replication licensing factor MCM7 OS=Mus musculus GN=Mcm7 PE=2 SV=1	MCM7_MOUSE	81 kDa	32.38	5.82	27.27	5.68	0.84			0% (0.34)
512	Ribosome biogenesis protein BOP1 OS=Mus musculus GN=Bop1 PE=1 SV=1	BOP1_MOUSE	83 kDa	4.17	7.22	3.51	6.07	0.84			0% (0.91)
513	60S ribosomal protein L15 OS=Mus musculus GN=Rpl15 PE=2 SV=4	RL15_MOUSE	24 kDa	10.49	5.87	8.74	0.3	0.83			0% (0.63)
514	WD40 repeat-containing protein SMU1 OS=Mus musculus GN=Smu1 PE=2 SV=2	SMU1_MOUSE	58 kDa	7.64	1.52	6.36	1	0.83			0% (0.29)
515	H/ACA ribonucleoprotein complex subunit 4 OS=Mus musculus GN=Dkc1 PE=1 SV=4	DKC1_MOUSE	57 kDa	5.47	4.75	4.54	4.14	0.83			0% (0.81)
516	Transformer-2 protein homolog alpha OS=Mus musculus GN=Tra2a PE=1 SV=1	TRA2A_MOUSE	32 kDa	3.67	1.59	3.04	1.05	0.83			0% (0.60)
517	Splicing factor 3B subunit 3 OS=Mus musculus GN=Sf3b3 PE=2 SV=1	SF3B3_MOUSE	136 kDa	18.88	4.05	15.61	4.71	0.83			0% (0.41)
518	Serine/arginine-rich splicing factor 7 OS=Mus musculus GN=Srsf7 PE=1 SV=1	SRSF7_MOUSE	31 kDa	17.41	3.37	14.28	2.87	0.82			0% (0.29)
519	Heat shock protein 105 kDa OS=Mus musculus GN=Hsph1 PE=1 SV=2	HS105_MOUSE	96 kDa	2.2	3.82	1.8	3.12	0.82			0% (0.89)
520	Tubulin beta-5 chain OS=Mus musculus GN=Tubb5 PE=1 SV=1	TBB5_MOUSE	50 kDa	27.34	17.41	22.32	11.43	0.82			0% (0.70)
521	p21-activated protein kinase-interacting protein 1 OS=Mus musculus GN=Pak1ip1 PE=2 SV=2	PK1IP_MOUSE	42 kDa	5.27	0.37	4.3	3.73	0.82			0% (0.68)
522	Nucleolar protein 56 OS=Mus musculus GN=Nop56 PE=1 SV=2	NOP56_MOUSE	64 kDa	25.83	3.65	20.98	5.51	0.81			0% (0.27)
523	ATP-dependent RNA helicase DDX24 OS=Mus musculus GN=Ddx24 PE=1 SV=2	DDX24_MOUSE	96 kDa	7.88	4.48	6.31	2.67	0.8			0% (0.63)
524	60S ribosomal protein L26 OS=Mus musculus GN=Rpl26 PE=2 SV=1	RL26_MOUSE	17 kDa	8.45	4.61	6.74	5.37	0.8			0% (0.70)
525	DNA replication licensing factor MCM2 OS=Mus musculus GN=Mcm2 PE=1 SV=3	MCM2_MOUSE	102 kDa	31.12	6.95	24.81	4.28	0.8			0% (0.25)
526	Probable U3 small nucleolar RNA-associated protein 11 OS=Mus musculus GN=Utp11l PE=2 SV=1	UTP11_MOUSE	31 kDa	1.32	2.29	1.05	1.82	0.8			0% (0.88)
527	Exosome component 10 OS=Mus musculus GN=Exosc10 PE=1 SV=2	EXOSX_MOUSE	101 kDa	1.32	1.15	1.05	1.82	0.79			0% (0.84)
528	Nucleolar RNA helicase 2 OS=Mus musculus GN=Ddx21 PE=1 SV=3	DDX21_MOUSE	94 kDa	84.72	16.66	67.16	25.89	0.79			0% (0.38)
529	Keratin, type I cytoskeletal 10 OS=Homo sapiens GN=KRT10 PE=1 SV=6	K1C10_HUMAN	59 kDa	43.32	45.88	34.29	14.74	0.79			0% (0.76)
530	Ig gamma-1 chain C region secreted form OS=Mus musculus GN=Ighg1 PE=1 SV=1	IGHG1_MOUSE	36 kDa	184.58	78.97	145.95	56.54	0.79			0% (0.53)
531	40S ribosomal protein S15a OS=Mus musculus GN=Rps15a PE=2 SV=2	RS15A_MOUSE	15 kDa	11.35	7.13	8.92	5.06	0.79			0% (0.66)
532	RNA-binding protein 39 OS=Mus musculus GN=Rbm39 PE=1 SV=2	RBM39_MOUSE	59 kDa	13.34	4.29	10.46	4.75	0.78			0% (0.48)
533	N-acetyltransferase 10 OS=Mus musculus GN=Nat10 PE=2 SV=1	NAT10_MOUSE	115 kDa	17.42	2.45	13.66	4.01	0.78			0% (0.24)
534	Keratin, type II cytoskeletal 2 epidermal OS=Homo sapiens GN=KRT2 PE=1 SV=2	K22E_HUMAN	65 kDa	40.7	23.56	31.88	15.54	0.78			0% (0.62)
535	60S ribosomal protein L18 OS=Mus musculus GN=Rpl18 PE=2 SV=3	RL18_MOUSE	22 kDa	20.43	13.19	15.99	9.31	0.78			0% (0.66)
536	DNA mismatch repair protein Msh2 OS=Mus musculus GN=Msh2 PE=2 SV=1	MSH2_MOUSE	104 kDa	3.34	3.15	2.6	2.28	0.78			0% (0.76)
537	Pre-mRNA-processing factor 6 OS=Mus musculus GN=Prpf6 PE=2 SV=1	PRP6_MOUSE	107 kDa	5.92	1.82	4.61	1.54	0.78			0% (0.39)
538	40S ribosomal protein S14 OS=Mus musculus GN=Rps14 PE=2 SV=3	RS14_MOUSE	16 kDa	9.8	3.16	7.59	3.97	0.77			0% (0.49)
539	Transformer-2 protein homolog beta OS=Mus musculus GN=Tra2b PE=1 SV=1	TRA2B_MOUSE	34 kDa	8.91	2.57	6.86	6.08	0.77			0% (0.62)
540	Cirhin OS=Mus musculus GN=Cirh1a PE=2 SV=3	CIR1A_MOUSE	77 kDa	2.34	2.48	1.8	1.67	0.77			0% (0.77)
541	DNA (cytosine-5)-methyltransferase 1 OS=Mus musculus GN=Dnmt1 PE=1 SV=5	DNMT1_MOUSE	183 kDa	53.6	6.78	41.17	4.42	0.77			0% (0.056)
542	GTP-binding nuclear protein Ran OS=Mus musculus GN=Ran PE=1 SV=3	RAN_MOUSE	24 kDa	22.45	2.83	17.23	3.61	0.77			0% (0.12)
543	Apoptosis inhibitor 5 OS=Mus musculus GN=Api5 PE=2 SV=2	API5_MOUSE	57 kDa	6.01	2.77	4.57	2.56	0.76			0% (0.54)
544	RNA-binding protein 26 OS=Mus musculus GN=Rbm26 PE=1 SV=2	RBM26_MOUSE	114 kDa	2.78	4.82	2.1	3.64	0.76			0% (0.86)
545	Keratin, type II cytoskeletal 1 OS=Homo sapiens GN=KRT1 PE=1 SV=6	K2C1_HUMAN	66 kDa	87.18	41.44	65.86	13.72	0.76			0% (0.45)
546	DNA topoisomerase 2-alpha OS=Mus musculus GN=Top2a PE=1 SV=2	TOP2A_MOUSE	173 kDa	75.11	21.5	56.66	10.57	0.75			0% (0.25)
547	Bcl-2-associated transcription factor 1 OS=Mus musculus GN=Bclaf1 PE=1 SV=2	BCLF1_MOUSE	106 kDa	15.11	3.27	11.39	1.69	0.75			0% (0.16)
548	SWI/SNF-related matrix-associated actin-dependent regulator of chromatin subfamily A member 5 OS=Mus muscu	SMCA5_MOUSE	122 kDa	23.43	4.88	17.5	5.23	0.75			0% (0.22)
549	60S ribosomal protein L17 OS=Mus musculus GN=Rpl17 PE=2 SV=3	RL17_MOUSE	21 kDa	7.11	4.68	5.3	2.79	0.75			0% (0.60)
550	U1 small nuclear ribonucleoprotein 70 kDa OS=Mus musculus GN=Snrnp70 PE=1 SV=2	RU17_MOUSE	52 kDa	5.86	3.47	4.37	0.15	0.75			0% (0.50)
551	DNA-(apurinic or apyrimidinic site) lyase OS=Mus musculus GN=Apex1 PE=1 SV=2	APEX1_MOUSE	35 kDa	4.35	1.74	3.2	3.16	0.74			0% (0.61)
552	Keratin, type II cytoskeletal 5 OS=Homo sapiens GN=KRT5 PE=1 SV=3	K2C5_HUMAN	62 kDa	10.29	14.68	7.56	10.84	0.73			0% (0.81)
553	Histone deacetylase complex subunit SAP18 OS=Mus musculus GN=Sap18 PE=1 SV=1	SAP18_MOUSE	18 kDa	5.87	7.15	4.3	3.73	0.73			0% (0.75)
554	60S ribosomal protein L10a OS=Mus musculus GN=Rpl10a PE=1 SV=3	RL10A_MOUSE	25 kDa	10.13	3.37	7.42	3.97	0.73			0% (0.42)
555	40S ribosomal protein S18 OS=Mus musculus GN=Rps18 PE=1 SV=3	RS18_MOUSE	18 kDa	10.38	5.62	7.54	4.93	0.73			0% (0.55)
556	Immunoglobulin G-binding protein G OS=Streptococcus sp. group G GN=spg PE=1 SV=1	SPG1_STRSG (+1)	48 kDa	9.66	8.38	6.97	6.06	0.72			0% (0.67)
557	WD repeat-containing protein 43 OS=Mus musculus GN=Wdr43 PE=1 SV=2	WDR43_MOUSE	75 kDa	7.91	2.58	5.67	1.1	0.72			0% (0.24)
558	Telomere-associated protein RIF1 OS=Mus musculus GN=Rif1 PE=1 SV=2	RIF1_MOUSE	266 kDa	1.26	2.18	0.9	1.56	0.71			0% (0.83)
559	Heterogeneous nuclear ribonucleoprotein L-like OS=Mus musculus GN=Hnrpll PE=1 SV=3	HNRLL_MOUSE	64 kDa	7.72	4.19	5.51	4.35	0.71			0% (0.56)
560	60S ribosomal protein L18a OS=Mus musculus GN=Rpl18a PE=1 SV=1	RL18A_MOUSE	21 kDa	9.2	3.36	6.54	2.99	0.71			0% (0.36)
561	40S ribosomal protein S23 OS=Mus musculus GN=Rps23 PE=2 SV=3	RS23_MOUSE	16 kDa	4.6	0.93	3.26	2.85	0.71			0% (0.48)
562	Ubiquitin carboxyl-terminal hydrolase isozyme L5 OS=Mus musculus GN=Uchl5 PE=2 SV=2	UCHL5_MOUSE	38 kDa	1.57	2.73	1.1	1.91	0.7			0% (0.82)
563	Keratin, type I cytoskeletal 9 OS=Homo sapiens GN=KRT9 PE=1 SV=3	K1C9_HUMAN	62 kDa	65.7	52.68	45.76	19.78	0.7			0% (0.57)
564	Protein RCC2 OS=Mus musculus GN=Rcc2 PE=2 SV=1	RCC2_MOUSE	56 kDa	49.28	8.38	33.87	4.76	0.69			0% (0.050)
565	ATP-dependent RNA helicase DDX18 OS=Mus musculus GN=Ddx18 PE=2 SV=1	DDX18_MOUSE	74 kDa	10.64	5.08	7.27	2.51	0.68			0% (0.36)
566	RRP12-like protein OS=Mus musculus GN=Rrp12 PE=1 SV=1	RRP12_MOUSE	143 kDa	1.32	2.29	0.9	1.56	0.68			0% (0.81)
567	U3 small nucleolar RNA-associated protein 14 homolog A OS=Mus musculus GN=Utp14a PE=2 SV=1	UT14A_MOUSE	87 kDa	3.09	3.78	2.1	3.64	0.68			0% (0.76)
568	Spliceosome RNA helicase Ddx39b OS=Mus musculus GN=Ddx39b PE=1 SV=1	DX39B_MOUSE	49 kDa	34.88	6.79	23.69	8.7	0.68			0% (0.15)
569	U3 small nucleolar ribonucleoprotein MPP10 OS=Mus musculus GN=Mphosph10 PE=1 SV=2	MPP10_MOUSE	79 kDa	1.04	1.81	0.7	1.21	0.67			0% (0.80)
570	Nuclease-sensitive element-binding protein 1 OS=Mus musculus GN=Ybx1 PE=1 SV=3	YBOX1_MOUSE	36 kDa	1.04	1.81	0.7	1.21	0.67			0% (0.80)
571	Something about silencing protein 10 OS=Mus musculus GN=Utp3 PE=1 SV=1	SAS10_MOUSE	53 kDa	1.04	1.81	0.7	1.21	0.67			0% (0.80)
572	Pre-mRNA-processing-splicing factor 8 OS=Mus musculus GN=Prpf8 PE=1 SV=2	PRP8_MOUSE	274 kDa	32	4.01	21.36	2.18	0.67			95% (0.016)
573	60S ribosomal protein L13a OS=Mus musculus GN=Rpl13a PE=1 SV=4	RL13A_MOUSE	23 kDa	10.13	4.44	6.73	6.16	0.66			0% (0.48)
574	40S ribosomal protein S3a OS=Mus musculus GN=Rps3a PE=1 SV=3	RS3A_MOUSE	30 kDa	12.68	6.49	8.42	5.67	0.66			0% (0.44)
575	PHD finger-like domain-containing protein 5A OS=Mus musculus GN=Phf5a PE=1 SV=1	PHF5A_MOUSE	12 kDa	2.26	2.04	1.5	2.6	0.66			0% (0.71)
576	ATP-citrate synthase OS=Mus musculus GN=Acly PE=1 SV=1	ACLY_MOUSE	120 kDa	3.57	1.87	2.37	2.22	0.66			0% (0.51)
577	T-complex protein 1 subunit zeta OS=Mus musculus GN=Cct6a PE=1 SV=3	TCPZ_MOUSE	58 kDa	5.75	5.67	3.8	4.08	0.66			0% (0.65)
578	Eukaryotic initiation factor 4A-III OS=Mus musculus GN=Eif4a3 PE=2 SV=3	IF4A3_MOUSE	47 kDa	36.08	11.26	23.67	5.93	0.66			0% (0.17)
579	T-complex protein 1 subunit alpha OS=Mus musculus GN=Tcp1 PE=1 SV=3	TCPA_MOUSE	60 kDa	6.25	1.31	4.08	1.25	0.65			0% (0.11)
580	60S ribosomal protein L11 OS=Mus musculus GN=Rpl11 PE=1 SV=4	RL11_MOUSE	20 kDa	9.17	2.54	5.95	2.54	0.65			0% (0.20)
581	T-complex protein 1 subunit theta OS=Mus musculus GN=Cct8 PE=1 SV=3	TCPQ_MOUSE	60 kDa	9.1	5.21	5.9	3.51	0.65			0% (0.43)
582	DNA replication licensing factor MCM4 OS=Mus musculus GN=Mcm4 PE=2 SV=1	MCM4_MOUSE	97 kDa	26.17	11.61	16.8	6.05	0.64			0% (0.28)
583	Nuclear pore complex protein Nup50 OS=Mus musculus GN=Nup50 PE=1 SV=3	NUP50_MOUSE	49 kDa	4.35	1.74	2.77	1.42	0.64			0% (0.29)
584	Squamous cell carcinoma antigen recognized by T-cells 3 OS=Mus musculus GN=Sart3 PE=2 SV=1	SART3_MOUSE	110 kDa	0.94	1.63	0.6	1.04	0.64			0% (0.77)
585	40S ribosomal protein S9 OS=Mus musculus GN=Rps9 PE=2 SV=3	RS9_MOUSE	23 kDa	8.71	6.13	5.52	3.09	0.63			0% (0.47)
586	DNA topoisomerase 2-beta OS=Mus musculus GN=Top2b PE=1 SV=2	TOP2B_MOUSE	182 kDa	17.2	12.21	10.8	10.21	0.63			0% (0.52)
587	Structural maintenance of chromosomes protein 1A OS=Mus musculus GN=Smc1a PE=1 SV=4	SMC1A_MOUSE	143 kDa	31.27	4.19	19.54	2.6	0.63			95% (0.015)
588	40S ribosomal protein S8 OS=Mus musculus GN=Rps8 PE=1 SV=2	RS8_MOUSE	24 kDa	8.52	2.44	5.32	0.96	0.63			0% (0.10)

589	Structural maintenance of chromosomes protein 5 OS=Mus musculus GN=Smc5 PE=2 SV=1	SMC5_MOUSE	129 kDa	4.49	3.97	2.8	3.41	0.62			0% (0.60)
590	40S ribosomal protein S6 OS=Mus musculus GN=Rps6 PE=1 SV=1	RS6_MOUSE	29 kDa	12.41	5.49	7.65	3.9	0.62			0% (0.29)
591	Suppressor of SWI4 1 homolog OS=Mus musculus GN=Ppan PE=2 SV=2	SSF1_MOUSE	53 kDa	1.7	1.58	1.05	1.82	0.62			0% (0.66)
592	60S ribosomal protein L9 OS=Mus musculus GN=Rpl9 PE=2 SV=2	RL9_MOUSE	22 kDa	4.92	1.9	2.98	0.71	0.61			0% (0.17)
593	60S ribosomal protein L19 OS=Mus musculus GN=Rpl19 PE=1 SV=1	RL19_MOUSE	23 kDa	6.51	4.01	3.94	3.43	0.61			0% (0.45)
594	Protein DEK OS=Mus musculus GN=Dek PE=1 SV=1	DEK_MOUSE	43 kDa	11.21	1.22	6.71	0.46	0.6			95% (0.0039)
595	Nucleolar GTP-binding protein 2 OS=Mus musculus GN=Gnl2 PE=2 SV=2	NOG2_MOUSE	83 kDa	4.94	0.9	2.95	2.69	0.6			0% (0.29)
596	Lamina-associated polypeptide 2, isoforms alpha/zeta OS=Mus musculus GN=Tmpo PE=1 SV=4	LAP2A_MOUSE	75 kDa	13.83	3.62	8.24	3.78	0.6			0% (0.14)
597	Protein VPRBP OS=Mus musculus GN=Vprbp PE=2 SV=4	VPRBP_MOUSE	169 kDa	2.25	1.99	1.33	1.17	0.59			0% (0.53)
598	40S ribosomal protein SA OS=Mus musculus GN=Rpsa PE=1 SV=4	RSSA_MOUSE	33 kDa	4.54	4.73	2.67	2.34	0.59			0% (0.57)
599	40S ribosomal protein S7 OS=Mus musculus GN=Rps7 PE=2 SV=1	RS7_MOUSE	22 kDa	11.37	5.57	6.67	2.14	0.59			0% (0.24)
600	U3 small nucleolar RNA-associated protein 15 homolog OS=Mus musculus GN=Utp15 PE=2 SV=1	UTP15_MOUSE	59 kDa	3.06	2.72	1.78	1.62	0.58			0% (0.52)
601	60S ribosomal protein L4 OS=Mus musculus GN=Rpl4 PE=1 SV=3	RL4_MOUSE	47 kDa	19.83	3.49	11.46	5.8	0.58			0% (0.099)
602	60S ribosomal protein L7 OS=Mus musculus GN=Rpl7 PE=2 SV=2	RL7_MOUSE	31 kDa	19.39	17.39	11.2	9.71	0.58			0% (0.52)
603	60S ribosomal protein L3 OS=Mus musculus GN=Rpl3 PE=2 SV=3	RL3_MOUSE	46 kDa	25.38	12.98	14.62	6.33	0.58			0% (0.27)
604	Myosin-9 OS=Mus musculus GN=Myh9 PE=1 SV=4	MYH9_MOUSE	226 kDa	19.59	3.44	11.25	2.88	0.57			95% (0.032)
605	40S ribosomal protein S26 OS=Mus musculus GN=Rps26 PE=2 SV=3	RS26_MOUSE	13 kDa	10.06	7.13	5.67	3.1	0.56			0% (0.38)
606	Trypsin OS=Sus scrofa PE=1 SV=1	TRYP_PIG	24 kDa	29.93	27.25	16.82	29.14	0.56			0% (0.60)
607	116 kDa U5 small nuclear ribonucleoprotein component OS=Mus musculus GN=Eftud2 PE=2 SV=1	U5S1_MOUSE	109 kDa	21.02	9.11	11.74	1.09	0.56			0% (0.15)
608	Superkiller viralicidic activity 2-like 2 OS=Mus musculus GN=Skiv2l2 PE=2 SV=1	SK2L2_MOUSE	118 kDa	3.77	4.23	2.1	3.64	0.56			0% (0.63)
609	Mortality factor 4-like protein 2 OS=Mus musculus GN=Morf4l2 PE=1 SV=1	MO4L2_MOUSE	32 kDa	1.89	3.27	1.05	1.82	0.56			0% (0.72)
610	Putative ATP-dependent RNA helicase DHX33 OS=Mus musculus GN=Dhx33 PE=1 SV=1	DHX33_MOUSE	78 kDa	1.32	2.29	0.73	1.27	0.56			0% (0.72)
611	Serum albumin OS=Mus musculus GN=Alb PE=1 SV=3	ALBU_MOUSE	69 kDa	3.82	6.62	2.1	3.64	0.55			0% (0.71)
612	Structural maintenance of chromosomes protein 3 OS=Mus musculus GN=Smc3 PE=1 SV=2	SMC3_MOUSE	142 kDa	31.8	6.93	17.46	3.31	0.55			95% (0.032)
613	40S ribosomal protein S11 OS=Mus musculus GN=Rps11 PE=2 SV=3	RS11_MOUSE	18 kDa	14.28	8.77	7.84	5.33	0.55			0% (0.34)
614	Nucleolar protein 58 OS=Mus musculus GN=Nop58 PE=1 SV=1	NOP58_MOUSE	60 kDa	17.67	4.62	9.69	3.84	0.55			0% (0.083)
615	60S ribosomal protein L13 OS=Mus musculus GN=Rpl13 PE=2 SV=3	RL13_MOUSE	24 kDa	16.47	5.49	9	2.38	0.55			0% (0.097)
616	60S ribosomal protein L6 OS=Mus musculus GN=Rpl6 PE=1 SV=3	RL6_MOUSE	34 kDa	19.69	6.42	10.71	0.92	0.54			0% (0.075)
617	60S ribosomal protein L5 OS=Mus musculus GN=Rpl5 PE=1 SV=3	RL5_MOUSE	34 kDa	12.39	5.33	6.69	3.08	0.54			0% (0.18)
618	Keratin, type II cytoskeletal 6A OS=Homo sapiens GN=KRT6A PE=1 SV=3	K2C6A_HUMAN	60 kDa	12.35	14.38	6.66	11.54	0.54			0% (0.62)
619	rRNA 2'-O-methyltransferase fibrillarin OS=Mus musculus GN=Fbl PE=1 SV=2	FBRL_MOUSE	34 kDa	20.13	2.26	10.79	1.32	0.54			95% (0.0035)
620	Plastin-2 OS=Mus musculus GN=Lcp1 PE=1 SV=4	PLSL_MOUSE	70 kDa	5.77	5.04	3.09	1.89	0.53			0% (0.44)
621	Keratin, type II cytoskeletal 5 OS=Mus musculus GN=Krt5 PE=1 SV=1	K2C5_MOUSE	62 kDa	12.92	11.74	6.86	9.64	0.53			0% (0.53)
622	U5 small nuclear ribonucleoprotein 200 kDa helicase OS=Homo sapiens GN=SNRNP200 PE=1 SV=2	U520_HUMAN	245 kDa	1.32	1.15	0.7	1.21	0.53			0% (0.55)
623	Scaffold attachment factor B2 OS=Mus musculus GN=Safb2 PE=1 SV=2	SAFB2_MOUSE	112 kDa	6.81	6.81	3.54	4.36	0.52			0% (0.52)
624	40S ribosomal protein S4, X isoform OS=Mus musculus GN=Rps4x PE=2 SV=2	RS4X_MOUSE	30 kDa	18.41	2.41	9.56	2.2	0.52			95% (0.0093)
625	Paired amphipathic helix protein Sin3a OS=Mus musculus GN=Sin3a PE=1 SV=3	SIN3A_MOUSE	145 kDa	1.35	1.17	0.7	1.21	0.52			0% (0.54)
626	SUMO-activating enzyme subunit 1 OS=Mus musculus GN=Sae1 PE=2 SV=1	SAE1_MOUSE	39 kDa	2.91	2.58	1.5	2.6	0.52			0% (0.54)
627	ATP-dependent RNA helicase DDX39A OS=Mus musculus GN=Ddx39a PE=2 SV=1	DX39A_MOUSE	49 kDa	32.05	10.99	16.57	16.31	0.52			0% (0.24)
628	40S ribosomal protein S10 OS=Mus musculus GN=Rps10 PE=1 SV=1	RS10_MOUSE	19 kDa	5.88	3.91	3.03	2.79	0.52			0% (0.36)
629	Transcription elongation regulator 1 OS=Mus musculus GN=Tcerg1 PE=1 SV=2	TCRG1_MOUSE	124 kDa	5.08	4.65	2.6	2.28	0.51			0% (0.45)
630	DNA-directed RNA polymerase II subunit RPB2 OS=Mus musculus GN=Polr2b PE=2 SV=2	RPB2_MOUSE	134 kDa	3.26	1.34	1.65	1.58	0.51			0% (0.25)
631	U5 small nuclear ribonucleoprotein 200 kDa helicase OS=Mus musculus GN=Snrnp200 PE=2 SV=1	U520_MOUSE	0 kDa	43.81	2.87	22.16	4.19	0.51			95% (0.0018)
632	Keratin, type I cytoskeletal 42 OS=Mus musculus GN=Krt42 PE=1 SV=1	K1C42_MOUSE	50 kDa	8.34	14.44	4.21	7.29	0.5			0% (0.68)
633	DNA ligase 1 OS=Mus musculus GN=Lig1 PE=1 SV=2	DNLI1_MOUSE	102 kDa	10.12	5.59	5.1	4.21	0.5			0% (0.28)
634	Zinc finger protein 638 OS=Mus musculus GN=Znf638 PE=1 SV=2	ZN638_MOUSE	218 kDa	2.08	3.61	1.05	1.82	0.5			0% (0.68)
635	40S ribosomal protein S2 OS=Mus musculus GN=Rps2 PE=1 SV=3	RS2_MOUSE	31 kDa	14.69	6.55	7.37	4.67	0.5			0% (0.19)
636	60S ribosomal protein L7a OS=Mus musculus GN=Rpl7a PE=2 SV=2	RL7A_MOUSE	30 kDa	28.32	17.07	14.12	6.68	0.5			0% (0.25)
637	40S ribosomal protein S3 OS=Mus musculus GN=Rps3 PE=1 SV=1	RS3_MOUSE	27 kDa	30.21	6.54	14.91	4.65	0.49			95% (0.030)
638	Cell growth-regulating nucleolar protein OS=Mus musculus GN=Lyar PE=1 SV=2	LYAR_MOUSE	44 kDa	4.3	0.59	2.1	3.64	0.49			0% (0.36)
639	Tyrosine-protein kinase BAZ1B OS=Mus musculus GN=Baz1b PE=1 SV=2	BAZ1B_MOUSE	171 kDa	9.84	2.13	4.75	2.26	0.48			95% (0.047)
640	DNA topoisomerase 1 OS=Mus musculus GN=Top1 PE=1 SV=2	TOP1_MOUSE	91 kDa	14.22	4.69	6.84	3.5	0.48			0% (0.094)
641	Pre-mRNA-splicing factor 38A OS=Mus musculus GN=Prpf38a PE=1 SV=1	PR38A_MOUSE	37 kDa	4	1.19	1.9	1.81	0.48			0% (0.17)
642	DNA-directed RNA polymerase II subunit RPB1 OS=Mus musculus GN=Polr2a PE=1 SV=3	RPB1_MOUSE	217 kDa	3.51	3.8	1.63	1.44	0.47			0% (0.47)
643	Probable ATP-dependent RNA helicase DDX56 OS=Mus musculus GN=Ddx56 PE=2 SV=1	DDX56_MOUSE	61 kDa	3.04	2.66	1.4	2.43	0.46			0% (0.48)
644	Histone H3.2 OS=Mus musculus GN=Hist1h3b PE=1 SV=2	H32_MOUSE	15 kDa	44.22	31.28	20.21	12.06	0.46			0% (0.28)
645	Serum albumin OS=Bos taurus GN=ALB PE=1 SV=4	ALBU_BOVIN	69 kDa	23.11	21.11	10.39	10.17	0.45			0% (0.40)
646	Ubiquitin-40S ribosomal protein S27a OS=Mus musculus GN=Rps27a PE=1 SV=2	RS27A_MOUSE	18 kDa	40.99	18.81	18.41	3.23	0.45			0% (0.11)
647	Ig heavy chain V region AC38 205.12 OS=Mus musculus PE=1 SV=1	HVM51_MOUSE	13 kDa	12.89	1.78	5.76	5.15	0.45			0% (0.086)
648	60S ribosomal protein L8 OS=Mus musculus GN=Rpl8 PE=2 SV=2	RL8_MOUSE	28 kDa	18.19	10.36	8.09	0.72	0.44			0% (0.17)
649	Ig gamma-2B chain C region OS=Mus musculus GN=Igh-3 PE=1 SV=3	IGG2B_MOUSE	44 kDa	10.3	12.34	4.56	7.89	0.44			0% (0.53)
650	Keratin, type I cytoskeletal 10 OS=Mus musculus GN=Krt10 PE=1 SV=3	K1C10_MOUSE	58 kDa	16.51	10.55	7.01	12.14	0.42			0% (0.36)
651	DNA repair protein RAD50 OS=Mus musculus GN=Rad50 PE=1 SV=1	RAD50_MOUSE	153 kDa	4.25	1.3	1.8	3.12	0.42			0% (0.28)
652	Keratin, type I cytoskeletal 16 OS=Homo sapiens GN=KRT16 PE=1 SV=4	K1C16_HUMAN	51 kDa	14.73	16.34	6.09	5.28	0.41			0% (0.43)
653	Keratin, type I cytoskeletal 17 OS=Mus musculus GN=Krt17 PE=1 SV=3	K1C17_MOUSE	48 kDa	10.26	11.01	4.21	7.29	0.41			0% (0.47)
654	Enhancer of rudimentary homolog OS=Mus musculus GN=Erh PE=1 SV=1	ERH_MOUSE	12 kDa	2.2	3.82	0.9	1.56	0.41			0% (0.61)
655	60S ribosomal protein L28 OS=Mus musculus GN=Rpl28 PE=1 SV=2	RL28_MOUSE	16 kDa	9.76	7.39	3.9	6.76	0.4			0% (0.37)
656	Guanine nucleotide-binding protein subunit beta-2-like 1 OS=Mus musculus GN=Gnb2l1 PE=1 SV=3	GBLP_MOUSE	35 kDa	10.3	2.39	4.11	4.06	0.4			0% (0.085)
657	40S ribosomal protein S24 OS=Mus musculus GN=Rps24 PE=1 SV=1	RS24_MOUSE	15 kDa	4.53	4.08	1.8	3.12	0.4			0% (0.41)
658	Activity-dependent neuroprotector homeobox protein OS=Mus musculus GN=Adnp PE=2 SV=1	ADNP_MOUSE	92 kDa	2.26	2.04	0.9	1.56	0.4			0% (0.41)
659	40S ribosomal protein S20 OS=Mus musculus GN=Rps20 PE=1 SV=1	RS20_MOUSE	13 kDa	2.69	2.5	1.05	1.82	0.39			0% (0.41)
660	60S ribosomal protein L24 OS=Mus musculus GN=Rpl24 PE=2 SV=2	RL24_MOUSE	18 kDa	4.89	1.74	1.9	1.81	0.39			0% (0.11)
661	T-complex protein 1 subunit delta OS=Mus musculus GN=Cct4 PE=1 SV=3	TCPD_MOUSE	58 kDa	1.57	2.73	0.6	1.04	0.38			0% (0.59)
662	Bromodomain adjacent to zinc finger domain protein 1A OS=Mus musculus GN=Baz1a PE=1 SV=3	BAZ1A_MOUSE	178 kDa	1.57	2.73	0.6	1.04	0.38			0% (0.59)
663	Replication factor C subunit 1 OS=Mus musculus GN=Rfc1 PE=1 SV=2	RFC1_MOUSE	126 kDa	1.62	1.5	0.6	1.04	0.37			0% (0.39)
664	Keratin, type I cytoskeletal 14 OS=Homo sapiens GN=KRT14 PE=1 SV=4	K1C14_HUMAN	52 kDa	17.14	19.63	6.31	10.93	0.37			0% (0.45)
665	Thyroid hormone receptor-associated protein 3 OS=Mus musculus GN=Thrap3 PE=1 SV=1	TR150_MOUSE	108 kDa	21.29	11.13	7.79	0.88	0.37			0% (0.10)
666	Serine/arginine repetitive matrix protein 2 OS=Mus musculus GN=Srrm2 PE=1 SV=3	SRRM2_MOUSE	295 kDa	12.93	7.88	4.71	4.97	0.36			0% (0.20)
667	60S ribosomal protein L23a OS=Mus musculus GN=Rpl23a PE=1 SV=1	RL23A_MOUSE	18 kDa	11.03	6.13	4.01	0.63	0.36			0% (0.12)
668	Nucleolar protein 11 OS=Mus musculus GN=Nol11 PE=2 SV=1	NOL11_MOUSE	81 kDa	1.98	0.1	0.7	1.21	0.35			0% (0.14)
669	SAP domain-containing ribonucleoprotein OS=Mus musculus GN=Sarnp PE=1 SV=3	SARNP_MOUSE	24 kDa	5.27	1.46	1.8	1.67	0.34			0% (0.053)
670	Histone H1.3 OS=Mus musculus GN=Hist1h1d PE=1 SV=2	H13_MOUSE	22 kDa	60.4	20.59	20.55	5.37	0.34			95% (0.032)
671	Nuclear RNA export factor 1 OS=Mus musculus GN=Nxf1 PE=1 SV=3	NXF1_MOUSE	70 kDa	3.09	3.78	1.05	1.82	0.34			0% (0.45)
672	Heat shock 70 kDa protein 4 OS=Mus musculus GN=Hspa4 PE=1 SV=1	HSP74_MOUSE	94 kDa	4.92	4.4	1.65	1.58	0.34			0% (0.29)
673	40S ribosomal protein S15 OS=Mus musculus GN=Rps15 PE=2 SV=2	RS15_MOUSE	17 kDa	1.89	3.27	0.6	1.04	0.32			0% (0.55)
674	60S ribosomal protein L37a OS=Mus musculus GN=Rpl37a PE=2 SV=2	RL37A_MOUSE	10 kDa	1.92	1.89	0.6	1.04	0.31			0% (0.35)
675	Vimentin OS=Mus musculus GN=Vim PE=1 SV=3	VIME_MOUSE	54 kDa	32.78	10.68	10.23	4.81	0.31			95% (0.029)
676	60S ribosomal protein L32 OS=Mus musculus GN=Rpl32 PE=2 SV=2	RL32_MOUSE	16 kDa	2.89	2.53	0.9	1.56	0.31			0% (0.31)
677	60S ribosomal protein L36 OS=Mus musculus GN=Rpl36 PE=2 SV=2	RL36_MOUSE	12 kDa	7.4	6.42	2.3	2	0.31			0% (0.26)
678	WD repeat-containing protein 46 OS=Mus musculus GN=Wdr46 PE=2 SV=1	WDR46_MOUSE	69 kDa	2.38	2.15	0.73	1.27	0.31			0% (0.32)
679	Elongation factor 1-gamma OS=Mus musculus GN=Eef1g PE=1 SV=3	EF1G_MOUSE	50 kDa	7.87	2.4	2.4	4.16	0.31			0% (0.12)
680	Transitional endoplasmic reticulum ATPase OS=Mus musculus GN=Vcp PE=1 SV=4	TERA_MOUSE	89 kDa	4.54	4.73	1.33	1.17	0.29			0% (0.32)
681	Histone H4 OS=Mus musculus GN=Hist1h4a PE=1 SV=2	H4_MOUSE	11 kDa	136.83	87.82	40.05	18.32	0.29			0% (0.14)
682	RNA-binding protein 25 OS=Mus musculus GN=Rbm25 PE=1 SV=1	RBM25_MOUSE	100 kDa	5.26	0.95	1.5	2.6	0.29			0% (0.079)
683	Histone H2B type 1-C/E/G OS=Mus musculus GN=Hist1h2bc PE=1 SV=3	H2B1C_MOUSE (+1	14 kDa	100.39	69.89	26.65	16.26	0.27			0% (0.15)
684	Histone H2A.x OS=Mus musculus GN=H2afx PE=1 SV=2	H2AX_MOUSE	15 kDa	52.74	32.32	13.91	12.81	0.26			0% (0.13)
685	60S ribosomal protein L36a OS=Mus musculus GN=Rpl36a PE=3 SV=2	RL36A_MOUSE	12 kDa	2.28	2.49	0.6	1.04	0.26			0% (0.34)
686	Proliferation-associated protein 2G4 OS=Mus musculus GN=Pa2g4 PE=1 SV=3	PA2G4_MOUSE	44 kDa	19.92	12.67	5.14	4.96	0.26			0% (0.13)
687	Lamin-B receptor OS=Mus musculus GN=Lbr PE=1 SV=2	LBR_MOUSE	71 kDa	13.11	3.23	3.29	1.16	0.25			95% (0.0077)
688	60S ribosomal protein L31 OS=Mus musculus GN=Rpl31 PE=2 SV=1	RL31_MOUSE	14 kDa	2.96	0.95	0.7	1.21	0.24			0% (0.064)
689	Pinin OS=Mus musculus GN=Pnn PE=1 SV=4	PININ_MOUSE	82 kDa	2.59	2.48	0.6	1.04	0.23			0% (0.27)
690	Histone H1.5 OS=Mus musculus GN=Hist1h1b PE=1 SV=2	H15_MOUSE	23 kDa	32.75	12.01	7.22	3.85	0.22			95% (0.025)
691	60S ribosomal protein L10-like OS=Mus musculus GN=Rpl10l PE=2 SV=1	RL10L_MOUSE (+1)	25 kDa	3.22	2.79	0.6	1.04	0.19			0% (0.20)
692	Histone H1.2 OS=Mus musculus GN=Hist1h1c PE=1 SV=2	H12_MOUSE	21 kDa	47.3	41.05	8.8	15.24	0.19			0% (0.20)
693	Lamin-B1 OS=Mus musculus GN=Lmnb1 PE=1 SV=3	LMNB1_MOUSE	67 kDa	34.58	4.18	6.34	3.69	0.18			95% (0.00093)
694	Core histone macro-H2A.1 OS=Mus musculus GN=H2afy PE=1 SV=3	H2AY_MOUSE	40 kDa	11.76	4.54	2.1	3.64	0.18			95% (0.045)
695	Serine/arginine repetitive matrix protein 1 OS=Mus musculus GN=Srrm1 PE=1 SV=2	SRRM1_MOUSE	107 kDa	3.61	2.04	0.6	1.04	0.17			0% (0.085)
696	Sister chromatid cohesion protein PDS5 homolog B OS=Mus musculus GN=Pds5b PE=1 SV=1	PDS5B_MOUSE	164 kDa	5.16	3.82	0.73	1.27	0.14			0% (0.13)
697	Histone H2A.V OS=Mus musculus GN=H2afv PE=1 SV=3	H2AV_MOUSE (+1)	14 kDa	34.5	15.16	4.81	8.33	0.14			95% (0.041)
698	Importin subunit alpha-1 OS=Mus musculus GN=Kpna1 PE=1 SV=2	IMA1_MOUSE	60 kDa	18.81	9.4	2.6	2.33	0.14			95% (0.044)
699	Histone H1.4 OS=Mus musculus GN=Hist1h1e PE=1 SV=2	H14_MOUSE	22 kDa	56.76	22.19	6.91	11.96	0.12			95% (0.027)
700	Importin subunit beta-1 OS=Mus musculus GN=Kpnb1 PE=1 SV=2	IMB1_MOUSE	97 kDa	7.59	1.44	0.9	1.56	0.12			95% (0.0055)
701	Histone H1.1 OS=Mus musculus GN=Hist1h1a PE=2 SV=2	H11_MOUSE	22 kDa	36.29	10.78	3.9	6.76	0.11			95% (0.012)
702	La-related protein 7 OS=Mus musculus GN=Larp7 PE=1 SV=2	LARP7_MOUSE	65 kDa	0.63	1.09	0	0	0			0% (0.37)
703	Myosin light polypeptide 6 OS=Mus musculus GN=Myl6 PE=1 SV=3	MYL6_MOUSE	17 kDa	0.63	1.09	0	0	0			0% (0.37)
704	Plasminogen activator inhibitor 1 RNA-binding protein OS=Mus musculus GN=Serbp1 PE=1 SV=2	PAIRB_MOUSE	45 kDa	0.63	1.09	0	0	0			0% (0.37)
705	Zinc finger CCCH domain-containing protein 18 OS=Mus musculus GN=Zc3h18 PE=1 SV=1	ZCH18_MOUSE	106 kDa	0.66	1.14	0	0	0			0% (0.37)
706	Annexin A4 OS=Mus musculus GN=Anxa4 PE=2 SV=4	ANXA4_MOUSE	36 kDa	0.66	1.14	0	0	0			0% (0.37)
707	Tropomodulin-3 OS=Mus musculus GN=Tmod3 PE=1 SV=1	TMOD3_MOUSE	40 kDa	0.66	1.14	0	0	0			0% (0.37)
708	ATP-dependent DNA helicase Q1 OS=Mus musculus GN=Recql PE=1 SV=2	RECQ1_MOUSE	72 kDa	0.66	1.14	0	0	0			0% (0.37)
709	Recombining binding protein suppressor of hairless OS=Mus musculus GN=Rbpj PE=1 SV=1	SUH_MOUSE	59 kDa	0.66	1.14	0	0	0			0% (0.37)
710	RNA polymerase I-specific transcription initiation factor RRN3 OS=Mus musculus GN=Rrn3 PE=1 SV=1	RRN3_MOUSE	75 kDa	0.66	1.14	0	0	0			0% (0.37)
711	Alpha-2-macroglobulin OS=Bos taurus GN=A2M PE=1 SV=2	A2MG_BOVIN	168 kDa	0.69	1.2	0	0	0			0% (0.37)
712	Pre-mRNA 3'-end-processing factor FIP1 OS=Mus musculus GN=Fip1l1 PE=1 SV=1	FIP1_MOUSE	65 kDa	0.69	1.2	0	0	0			0% (0.37)
713	Eukaryotic translation initiation factor 1A, X-chromosomal OS=Mus musculus GN=Eif1ay PE=2 SV=3	IF1AX_MOUSE (+7)	16 kDa	0.69	1.2	0	0	0			0% (0.37)
714	Kinesin-like protein KIF2C OS=Mus musculus GN=Kif2c PE=1 SV=1	KIF2C_MOUSE	81 kDa	0.69	1.2	0	0	0			0% (0.37)
715	Small nuclear ribonucleoprotein-associated protein B OS=Mus musculus GN=Snrpb PE=1 SV=1	RSMB_MOUSE (+1)	24 kDa	0.69	1.2	0	0	0			0% (0.37)
716	F-box-like/WD repeat-containing protein TBL1XR1 OS=Mus musculus GN=Tbl1xr1 PE=2 SV=1	TBL1R_MOUSE	56 kDa	0.69	1.2	0	0	0			0% (0.37)
717	THUMP domain-containing protein 1 OS=Mus musculus GN=Thumpd1 PE=1 SV=1	THUM1_MOUSE	39 kDa	0.69	1.2	0	0	0			0% (0.37)
718	Serine/threonine-protein kinase VRK1 OS=Mus musculus GN=Vrk1 PE=1 SV=2	VRK1_MOUSE	50 kDa	0.69	1.2	0	0	0			0% (0.37)
719	Dolichyl-diphosphooligosaccharide--protein glycosyltransferase subunit 1 OS=Mus musculus GN=Rpn1 PE=2 SV=1	RPN1_MOUSE	69 kDa	0.94	1.63	0	0	0			0% (0.37)
720	Cyclin-dependent kinase 11B OS=Mus musculus GN=Cdk11b PE=1 SV=2	CD11B_MOUSE	0 kDa	0.94	1.63	0	0	0			0% (0.37)
721	DDB1- and CUL4-associated factor 13 OS=Mus musculus GN=Dcaf13 PE=2 SV=2	DCA13_MOUSE	51 kDa	0.99	1.71	0	0	0			0% (0.37)
722	Digestive organ expansion factor homolog OS=Mus musculus GN=Diexf PE=2 SV=2	DIEXF_MOUSE	89 kDa	0.99	1.71	0	0	0			0% (0.37)
723	Ig kappa chain V19-17 OS=Mus musculus GN=Igk-V19-17 PE=1 SV=1	KV5A1_MOUSE	16 kDa	0.99	1.71	0	0	0			0% (0.37)
724	60S ribosomal protein L29 OS=Mus musculus GN=Rpl29 PE=2 SV=2	RL29_MOUSE	18 kDa	0.99	1.71	0	0	0			0% (0.37)
725	T-complex protein 1 subunit gamma OS=Mus musculus GN=Cct3 PE=1 SV=1	TCPG_MOUSE	61 kDa	1.04	1.81	0	0	0			0% (0.37)
726	Splicing factor 3A subunit 1 OS=Mus musculus GN=Sf3a1 PE=1 SV=1	SF3A1_MOUSE	89 kDa	1.04	1.81	0	0	0			0% (0.37)
727	RNA-binding protein with serine-rich domain 1 OS=Mus musculus GN=Rnps1 PE=2 SV=1	RNPS1_MOUSE	34 kDa	1.04	1.81	0	0	0			0% (0.37)
728	High-molecular weight cobalt-containing nitrile hydratase subunit alpha OS=Rhodococcus rhodochrous GN=nhhA P	NHA1_RHORH	23 kDa	1.04	1.81	0	0	0			0% (0.37)
729	RNA-binding protein 8A OS=Mus musculus GN=Rbm8a PE=1 SV=3	RBM8A_MOUSE	20 kDa	1.04	1.81	0	0	0			0% (0.37)
730	Protein Red OS=Mus musculus GN=Ik PE=2 SV=2	RED_MOUSE	66 kDa	1.04	1.81	0	0	0			0% (0.37)
731	Eukaryotic translation initiation factor 3 subunit L OS=Mus musculus GN=Eif3l PE=1 SV=1	EIF3L_MOUSE	67 kDa	1.26	2.18	0	0	0			0% (0.37)
732	Small nuclear ribonucleoprotein F OS=Mus musculus GN=Snrpf PE=2 SV=1	RUXF_MOUSE	10 kDa	1.26	2.18	0	0	0			0% (0.37)
733	THO complex subunit 4 OS=Mus musculus GN=Alyref PE=1 SV=3	THOC4_MOUSE	27 kDa	1.32	2.29	0	0	0			0% (0.37)
734	Hydroxymethylglutaryl-CoA synthase, cytoplasmic OS=Mus musculus GN=Hmgcs1 PE=1 SV=1	HMCS1_MOUSE	58 kDa	1.32	1.15	0	0	0			0% (0.12)
735	Arginine and glutamate-rich protein 1 OS=Mus musculus GN=Arglu1 PE=1 SV=2	ARGL1_MOUSE	33 kDa	1.35	1.17	0	0	0			0% (0.12)
736	Structural maintenance of chromosomes protein 6 OS=Mus musculus GN=Smc6 PE=2 SV=1	SMC6_MOUSE	127 kDa	1.35	1.17	0	0	0			0% (0.12)
737	Protein odr-4 homolog OS=Mus musculus GN=Odr4 PE=2 SV=2	ODR4_MOUSE	50 kDa	1.39	2.41	0	0	0			0% (0.37)
738	Eukaryotic translation initiation factor 3 subunit D OS=Mus musculus GN=Eif3d PE=1 SV=2	EIF3D_MOUSE	64 kDa	1.39	2.41	0	0	0			0% (0.37)
739	Eukaryotic translation initiation factor 3 subunit C OS=Mus musculus GN=Eif3c PE=1 SV=1	EIF3C_MOUSE	106 kDa	1.39	2.41	0	0	0			0% (0.37)

740	26S proteasome non-ATPase regulatory subunit 1 OS=Mus musculus GN=Psmd1 PE=1 SV=1	PSMD1_MOUSE	106 kDa	1.57	2.73	0	0	0			0% (0.37)
741	T-complex protein 1 subunit epsilon OS=Mus musculus GN=Cct5 PE=1 SV=1	TCPE_MOUSE	60 kDa	1.57	2.73	0	0	0			0% (0.37)
742	Staphylococcal nuclease domain-containing protein 1 OS=Mus musculus GN=Snd1 PE=1 SV=1	SND1_MOUSE	102 kDa	1.6	1.45	0	0	0			0% (0.13)
743	Protein SON OS=Mus musculus GN=Son PE=1 SV=2	SON_MOUSE	266 kDa	1.62	1.5	0	0	0			0% (0.14)
744	Transcriptional repressor CTCF OS=Mus musculus GN=Ctcf PE=1 SV=2	CTCF_MOUSE	84 kDa	1.64	1.47	0	0	0			0% (0.13)
745	Nucleolar protein 10 OS=Mus musculus GN=Nol10 PE=2 SV=1	NOL10_MOUSE	80 kDa	1.65	2.85	0	0	0			0% (0.37)
746	Nuclear receptor coactivator 5 OS=Mus musculus GN=Ncoa5 PE=1 SV=1	NCOA5_MOUSE	65 kDa	1.65	2.85	0	0	0			0% (0.37)
747	Nascent polypeptide-associated complex subunit alpha, muscle-specific form OS=Mus musculus GN=Naca PE=1 S	NACAM_MOUSE	220 kDa	1.65	2.85	0	0	0			0% (0.37)
748	Importin subunit alpha-2 OS=Mus musculus GN=Kpna2 PE=1 SV=2	IMA2_MOUSE	58 kDa	1.74	3.01	0	0	0			0% (0.37)
749	Keratin, type II cytoskeletal 4 OS=Homo sapiens GN=KRT4 PE=1 SV=4	K2C4_HUMAN	57 kDa	1.74	3.01	0	0	0			0% (0.37)
750	Putative RNA-binding protein Luc7-like 1 OS=Mus musculus GN=Luc7l PE=2 SV=2	LUC7L_MOUSE	44 kDa	1.98	3.42	0	0	0			0% (0.37)
751	PC4 and SFRS1-interacting protein OS=Mus musculus GN=Psip1 PE=1 SV=1	PSIP1_MOUSE	60 kDa	1.99	1.73	0	0	0			0% (0.12)
752	Keratin, type I cuticular Ha4 OS=Homo sapiens GN=KRT34 PE=2 SV=2	KRT34_HUMAN	49 kDa	2.08	3.61	0	0	0			0% (0.37)
753	tRNA (guanine(26)-N(2))-dimethyltransferase OS=Mus musculus GN=Trmt1 PE=1 SV=2	TRM1_MOUSE	72 kDa	2.2	3.82	0	0	0			0% (0.37)
754	Ribosome biogenesis regulatory protein homolog OS=Mus musculus GN=Rrs1 PE=2 SV=1	RRS1_MOUSE	42 kDa	2.28	2.49	0	0	0			0% (0.19)
755	Serine/threonine-protein kinase PRP4 homolog OS=Mus musculus GN=Prpf4b PE=1 SV=3	PRP4B_MOUSE	117 kDa	2.31	0.58	0	0	0			95% (0.0022)
756	Structural maintenance of chromosomes flexible hinge domain-containing protein 1 OS=Mus musculus GN=Smchd1	SMHD1_MOUSE	226 kDa	2.78	4.82	0	0	0			0% (0.37)
757	Keratin, type I cuticular Ha1 OS=Homo sapiens GN=KRT31 PE=2 SV=3	K1H1_HUMAN (+1)	47 kDa	2.78	4.82	0	0	0			0% (0.37)
758	Keratin, type II cuticular Hb1 OS=Homo sapiens GN=KRT81 PE=1 SV=3	KRT81_HUMAN	55 kDa	2.78	4.82	0	0	0			0% (0.37)
759	Probable ATP-dependent RNA helicase DDX47 OS=Mus musculus GN=Ddx47 PE=2 SV=2	DDX47_MOUSE	51 kDa	3	2.69	0	0	0			0% (0.13)
760	Phosphoribosylformylglycinamidine synthase OS=Mus musculus GN=Pfas PE=2 SV=1	PUR4_MOUSE	145 kDa	3	2.69	0	0	0			0% (0.13)
761	Eukaryotic translation initiation factor 3 subunit A OS=Mus musculus GN=Eif3a PE=1 SV=5	EIF3A_MOUSE	162 kDa	3.27	1.04	0	0	0			95% (0.0054)
762	Keratin, type II cuticular Hb6 OS=Homo sapiens GN=KRT86 PE=1 SV=1	KRT86_HUMAN	53 kDa	3.47	6.02	0	0	0			0% (0.37)
763	SAFB-like transcription modulator OS=Mus musculus GN=Sltm PE=1 SV=1	SLTM_MOUSE	117 kDa	4.1	4.17	0	0	0			0% (0.16)
764	Keratin, type I cuticular Ha1 OS=Mus musculus GN=Krt31 PE=2 SV=2	K1H1_MOUSE (+1)	47 kDa	4.17	7.22	0	0	0			0% (0.37)
765	Polymerase delta-interacting protein 3 OS=Mus musculus GN=Poldip3 PE=2 SV=1	PDIP3_MOUSE	46 kDa	4.41	3.88	0	0	0			0% (0.12)
766	Importin subunit alpha-3 OS=Mus musculus GN=Kpna3 PE=1 SV=1	IMA3_MOUSE	58 kDa	4.43	4.19	0	0	0			0% (0.14)
767	Lamina-associated polypeptide 2, isoforms beta/delta/epsilon/gamma OS=Mus musculus GN=Tmpo PE=1 SV=4	LAP2B_MOUSE	50 kDa	4.52	7.82	0	0	0			0% (0.37)
768	Apoptotic chromatin condensation inducer in the nucleus OS=Mus musculus GN=Acin1 PE=1 SV=3	ACINU_MOUSE	151 kDa	4.81	5.95	0	0	0			0% (0.23)
769	Keratin, type II cuticular Hb5 OS=Mus musculus GN=Krt85 PE=2 SV=2	KRT85_MOUSE	56 kDa	5.45	5.23	0	0	0			0% (0.15)
770	Heterochromatin protein 1-binding protein 3 OS=Mus musculus GN=Hp1bp3 PE=1 SV=1	HP1B3_MOUSE	61 kDa	6.82	4.86	0	0	0			0% (0.072)
771	Importin subunit alpha-4 OS=Mus musculus GN=Kpna4 PE=2 SV=1	IMA4_MOUSE	58 kDa	9.87	1.29	0	0	0			95% (0.00019)
772	Histone H3.1 OS=Mus musculus GN=Hist1h3a PE=1 SV=2	H31_MOUSE	15 kDa	15.42	26.71	0	0	0			0% (0.37)
773	Histone H2A type 1-F OS=Mus musculus GN=Hist1h2af PE=1 SV=3	H2A1F_MOUSE	14 kDa	42.98	38.84	0	0	0			0% (0.13)
774	LexA repressor OS=Escherichia coli O139:H28 (strain E24377A / ETEC) GN=lexA PE=3 SV=1	LEXA_ECO24 (+26)	22 kDa	85.59	33.97	0	0	0	3xFNLDD		95% (0.012)
